# Engineered systems of inducible anti-repressors for the next generation of biological programming

**DOI:** 10.1038/s41467-020-18302-1

**Published:** 2020-09-07

**Authors:** Thomas M. Groseclose, Ronald E. Rondon, Zachary D. Herde, Carlos A. Aldrete, Corey J. Wilson

**Affiliations:** Georgia Institute of Technology, School of Chemical & Biomolecular Engineering, Atlanta, GA USA

**Keywords:** Protein design, Synthetic biology, Genetic circuit engineering

## Abstract

Traditionally engineered genetic circuits have almost exclusively used naturally occurring transcriptional repressors. Recently, non-natural transcription factors (repressors) have been engineered and employed in synthetic biology with great success. However, transcriptional anti-repressors have largely been absent with regard to the regulation of genes in engineered genetic circuits. Here, we present a workflow for engineering systems of non-natural anti-repressors. In this study, we create 41 inducible anti-repressors. This collection of transcription factors respond to two distinct ligands, fructose (anti-FruR) or D-ribose (anti-RbsR); and were complemented by 14 additional engineered anti-repressors that respond to the ligand isopropyl β-d-1-thiogalactopyranoside (anti-LacI). In turn, we use this collection of anti-repressors and complementary genetic architectures to confer logical control over gene expression. Here, we achieved all NOT oriented logical controls (i.e., NOT, NOR, NAND, and XNOR). The engineered transcription factors and corresponding series, parallel, and series-parallel genetic architectures represent a nascent anti-repressor based transcriptional programming structure.

## Abstract (web summary)

Transcriptional anti-repressors have been largely absent in the design of regulated genetic circuits. Here, the authors present a workflow of the engineering of non-natural anti-reperssors that can be built into NOT oriented logic gates.

## Introduction

**Fig. 1:**
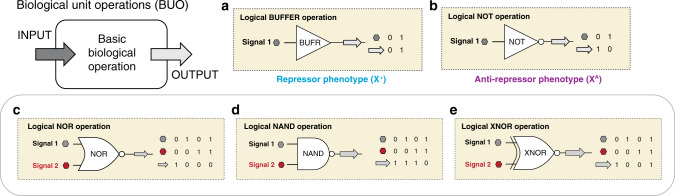
Defining biological unit operations (BUO). Similar to a unit operation in chemical engineering, a biological unit operation is a discrete process unit that converts an INPUT to a predictable OUTPUT, within a given performance boundary. A summary of biological Boolean logical operations developed (or employed) in this study is as follows: **a** the foundational logical BUFFER operation (i.e., BUFFER BUO), executed by the repressor (X^+^) phenotype. **b** The foundational logical NOT operation (i.e., NOT BUO), executed by the anti-repressor (X^A^) phenotype. BUFFER and NOT are the fundamental BUO used to construct combinatorial logic gates summarized in (**c**–**e**). Combinatorial logical operations, that leverage the NOT operations generated in this work: **c** The logical NOR operation; **d** the logical NAND operation; and **e** the logical XNOR operation.

In the burgeoning field of cellular programming, synthetic biologists seek to engineer systems that encode predictable and executable functions in response to environmental, cellular, and temporal cues (inputs)^1–3^. In principle, this is akin to engineering a computer program to carry out a desired task. Recent advances in synthetic biology have brought the fields of biomolecular, electrical, chemical, and computer engineering far closer^4–6^, for instance with the development of biological sensors^7–9^, memory^10–12^, switches and controllers^13–17^, transistors^18^, counters^19^, oscillators and clocks^20–24^, and equivalents of Boolean logic gates^25–27^. In many of these developments, transcriptional regulation is achieved via allosteric transcription factors (TFs)^28^. Classically, allosteric transcription factors are DNA-binding proteins that either facilitate (activate) or impede (block) gene transcription and subsequent expression by modulating the interaction between RNA polymerase (RNAP) and a promoter element in response to external stimuli. In this way, TFs can serve as fundamental units of gene circuits and networks by turning gene transcription off (0) or on (1). The most prevalent transcription factors include the lactose repressor (LacI), tetracycline repressor (TetR), and bacteriophage λ cI repressor—all with repressor phenotypes^24,27,29,30^. Repressors function by binding to a promoter and physically compromising binding by RNAP until acted upon by an exogenous signal^31^. In recent years, efforts have sought to increase cellular computing capacity and complexity through the development of additional tools for transcriptional regulation^32^. These TFs, though, have been widely confined to repressors functional with only a small number of operator (DNA) sequences, limiting their application in biological circuit engineering^33–37^. In one approach to expand biological circuits beyond simple repressors, a set of orthogonal TFs in the cI scaffold and promoters were engineered via directed evolution that exhibited dual regulatory activities, including activator-repressor activity^33^. While activators and repressors are mechanistically different, they share the same objective truth table—i.e., gene expression (output) is turned on (1) in the presence of the signal (input)—thus, can be regarded as biological BUFFER logic gates^26,38^, Fig. 1a.

An anti-repressor is mechanistically inverted relative to a repressor. Namely, an anti-repressor has an affinity for DNA that increases upon binding to a cognate ligand. Notably, anti-repressors function as objective biological NOT gates and, as such, they encompass a fundamental logical operation (antithetical to BUFFER logic), Fig. 1b. To the best of our knowledge, natural transcriptional anti-repressors—e.g., the purine repressor (PurR) and tryptophan repressor (TrpR)—are limited in number, and have seen minimal use in gene circuits. One recent study evolved systems of TFs in the TrpR scaffold to exhibit orthogonal, non-native ligand- and operator-binding activities, which were then intermolecularly tethered to create Boolean NAND gates^39^. The evolution of additional classes of anti-repressors has greatly expanded the relevance of these TFs in gene circuits. Engineered anti-repressors in the LacI scaffold (anti-lacs)^40^, for example, are emerging as robust tools in synthetic gene networks and have added significantly to a scarce set of regulatory tools. Similarly, an allosteric activity reversal in the TetR system has been accomplished with single residue mutations, generating revTetR variants which have been used in circuits for gene silencing and directed evolution of the quorum sensing (QS) master regulator LsrR, yielding activator aLsrR variants, and were used to construct a QS-mediated switch^41,42^.

Other work has sought to broaden the class of ligands to which TFs respond, facilitating the utilization of simultaneous and distinct input signals. Domain swapping of the regulatory core domains (RCD—responsible for ligand sensing and allosteric signal propagation) of several LacI/GalR transcription factor family members onto the LacI YQR DNA-binding domain (DBD—responsible for binding to the operator-DNA sequence and modulating transcription) gave rise to several chimeric TF variants which could regulate the *lac* promoter via the homologue’s natural effector ligand^43^. Further, the DNA operator element and complementary DBD have been simultaneously engineered to confer alternate DNA recognition (ADR), allowing the same RCD set to interact with disparate operators with minimal crosstalk. This collection of engineered allosteric transcriptional repressors presents an additional means for the expansion of biological computing capabilities. A similar modular design approach was used recently to engineer a collection of TFs not observed in nature with a range of DNA and ligand binding affinities. These TFs were then deployed in gene networks that developed the basic logical operations AND^25,43^, OR, NOT (inverted)^26,38^, and NOR (apparent)^25^, along with other combinatorial logical operations^25^. In addition, Chan et al. have used an analogous modular design strategy to engineer TF repressors for other biotechnological applications (e.g., Deadman and passcode kill switches)^38^. Recent work has verified engineered transcription factor applicability in not just the repressor phenotype, but also a library of anti-repressor TFs in the form of a collection of anti-lacs^40,44^.

Transcriptional anti-repressors are underutilized in synthetic biology, likely due to their rarity relative to repressors. Anti-inducible promoters can potentially offer significant advances in biological circuit complexity and scope. We posit that certain anti-repressor based biological unit operations (BUO) can simplify many aspects of genetic programming, Fig. 1. The proliferation of anti-repressor based BUO as regulatory tools promises to advance applications involving biosensing, gene silencing, and negative feedback loops. In this work, we present a workflow for engineering systems of non-natural transcriptional anti-repressors. Two classes of anti-repressors, responsive to fructose-1,6-phosphate (anti-FruR), and D-ribose (anti-RbsR), are presented here, each evolved via disparate protein engineering strategies. We then engineered these TFs with ADR, creating a library of 41 orthogonal anti-repressors that are compatible with robust well-characterized *lac*-mediated networks. In turn, these anti-repressors (along with previously engineered TFs) were coupled with complementary genetic architectures to confer higher-order control over gene expression. Namely, we achieved all NOT oriented Boolean logical operations (i.e., NOT (non-inverted logic), NAND, NOR, and XNOR), Fig. 1b–e. These engineered systems of TFs and genetic architectures represent a nascent anti-repressor based transcriptional programming language, complementing ongoing efforts to develop comprehensive transcriptional controls not observed in nature.

## Results

### Conferring super-repression in LacI homologues

**Fig. 2:**
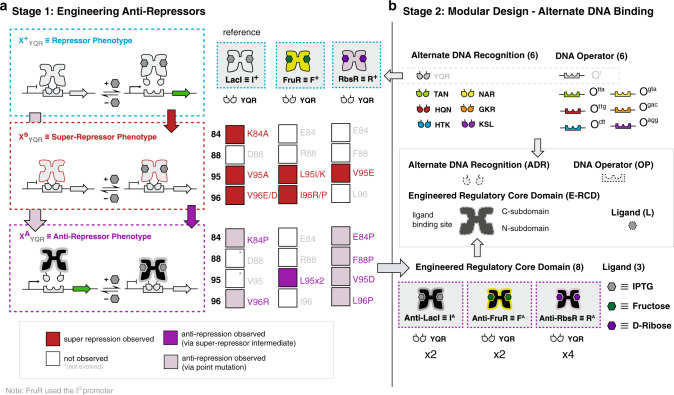
Workflow for engineering systems of transcriptional anti-repressors. **a** Left panel illustrates stage one of the workflow, where anti-repression was conferred to wild-type repressor scaffolds. We began with the repressor regulatory core domain (RCD) adapted with the wild-type LacI DNA-binding domain (DBD), YQR (X^+^_YQR_). The repressor phenotype (X^+^) allows gene transcription only in the presence of the inducer ligand, which causes the TF to dissociate from a cognate operator, permitting RNA polymerase function. A sequence alignment to the reference repressor, LacI ≡ I^+^, informed which residues in FruR ≡ F^+^ and RbsR ≡ R^+^ are target positions for rational engineering of super-repressors. The super-repressor phenotype (X^S^) inhibits gene transcription regardless of the presence of the inducer ligand by constitutively binding to the operator. Site-saturation at positions corresponding to LacI K84, D88, V95, and V96 yielded super-repressor phenotypes in homologues F^+^_YQR_ and R^+^_YQR_. This follows the route of the red arrow (X^+^ → X^S^). In addition, site-saturation of R^+^_YQR_ resulted in single-mutation R^A^_YQR_ anti-repressors, effectively bypassing the R^S^ intermediate (following the path of the light purple arrow - X^+^ → X^A^). The anti-repressor phenotype (X^A^) inhibits gene transcription in the presence of the ligand by binding to the operator. Anti-repression was also conferred via laboratory evolution, introducing compensatory mutations to the X^S^ intermediate (red, then dark purple arrow: X^+^ → X^S^ → X^A^) yielding F^A^_YQR_ TFs. **b** Right panel illustrates stage two of the workflow, where modular design was used to adapt engineered RCDs (E-RCDs) with alternate DNA recognition (ADR). E-RCDs from stage one, giving anti-repressor responses to ligands IPTG, fructose, and D-ribose were used as components of regulatory proteins. A regulatory protein consists of (i) an RCD, which is responsible for ligand binding and allosteric signal transmission and (ii) a DBD, which allows interaction with a DNA operator. We selected six ADR modules to adapt to our E-RCDs, which we anticipated would confer interaction with six orthogonal cognate operators, with cognate interactions matching in color. ADR is in addition to YQR | O^1^ interactions, with which the E-RCDs were originally screened.

Here we established a two part workflow to engineer non-natural anti-repressor transcription factors, combining two separate strategies that we established (in part) previously^25,40,44^ (Fig. 2). In this study our overarching design goal was to engineer a set of non-natural anti-repressors that complement and communally function with our current collection of non-natural repressor transcription factors^25,44^. In stage one of the workflow, we outlined the process for conferring anti-repression (Fig. 2a). We posited that anti-repression can be conferred in a given LacI/GalR repressor (X^+^_YQR_) scaffold: (i) via an initial block in allosteric communication resulting in a super-repressor phenotype (X^S^_YQR_), (ii) followed by evolution of the RCD to introduce compensatory mutation(s) conferring alternate allosteric communication (X^A^_YQR_) (Fig. 2a). The naming convention for the repressor (superscript, +) and super-repressor (superscript, S) phenotypes were adopted from Suckow et al.^45^, in which the original functional map of LacI was generated. Whereas, the anti-repressor phenotype descriptor (superscript, A) was introduced by Richards et al.^40^, as an extension of this foundational study. The X identifier denotes the regulatory core necessary for ligand binding (such that X ≡ I, R, or F in this study). Finally, the subscript YQR implies positions Y17, Q18, and R22 in the DNA-binding domain, and will also serve as the positions to confer alternate DNA binding (discussed in a later section)^46^. In the initial step of this workflow, we selected RbsR ≡ R and FruR ≡ F (in addition to the LacI ≡ I, reference system) repressor regulatory cores and adapted each RCD with a common DNA-binding domain (i.e., the native lactose repressor domain, YQR), which normalized the DNA-binding function (Supplementary Note 1). This resulted in R^+^_YQR_ and F^+^_YQR_, in addition to the reference repressor I^+^_YQR_ (Fig. 2a). The adapted transcription factors R^+^_YQR_ and F^+^_YQR_ have previously been reported as functional repressors^43^, and we confirmed these results in this study—thus establishing our parental input for the stage one workflow. To identify putative super-repressor positions in the R^+^_YQR_ and F^+^_YQR_ RCDs, we used a primary-structure sequence alignment relative to LacI. We hypothesized that reported super-repressor positions 84, 88, 95 and 96 in LacI are likely conserved in other LacI/GalR homologues (Supplementary Note 2). To identify putative super-repressor positions in RbsR and FruR, we performed a multiple sequence alignment of the primary structures relative to LacI (Supplementary Fig. 1). Sequence alignment revealed conservation at positions 84, 95, and 96—however, not position 88. In turn, we performed saturation mutagenesis at each amino acid position corresponding to 84, 88, 95, and 96 in all three scaffolds (R^+^_YQR_, F^+^_YQR_, and I^+^_YQR_), and screened for super-repression (Supplementary Fig. 2). Interestingly, we observed the super-repressor phenotype at all positions for the I_YQR_ scaffold, except position 88 (Supplementary Note 3). The F_YQR_ scaffold supported super-repression at source positions 95 and 96, while R_YQR_ only displayed the super-repression phenotype at source position 95 (Fig. 2a). In summary, we achieved allosteric blocks in all of the parental scaffolds, satisfying the initial step of the stage one workflow.

### Engineering transcriptional anti-repression

Our original workflow for conferring anti-repression in LacI was achieved by way of a super-repressor intermediate^40^. However, upon saturation mutagenesis at a given site, we observed conferred anti-repression in the R_YQR_ scaffold via a single mutation (i.e., without compensatory mutations) at all four source positions (Fig. 2a, Supplementary Fig. 3). All variants were evaluated via a proximal reporter architecture, in which the O^1^ operator was located downstream of the promoter element and upstream of a green fluorescent protein (GFP) reporter (Supplementary Fig. 4). This observation prompted us to re-assess the phenotype data for the reference system (I_YQR_) for similar results. In agreement, the I_YQR_ reference scaffold supported conferred anti-repression via a single mutation at positions 84 and 96 (Fig. 2a, Supplementary Fig. 3). However, no single mutant anti-repressors for the F_YQR_ parent scaffold were observed. Accordingly, we conducted error-prone PCR (EP-PCR) on each of the F^s^_YQR_ variants and screened via fluorescence-activated cell sorting (FACS), in the presence and absence of the effector ligand fructose. Putative F^A^_YQR_ anti-repressors were evaluated in a second round via a microwell plate assay, and confirmed variants were DNA sequenced. In turn, using the original engineering workflow (X^+^_YQR_ → X^S^_YQR_ → X^A^_YQR_), we discovered two F^A^_YQR_ anti-repressors (Fig. 2a, Supplementary Fig. 3). The objective of stage one of the workflow was to generate at least one anti-repressor for each parent scaffold. Accordingly, no additional anti-repressors were generated beyond the aforementioned.

### Alternate DNA binding via modular design

**Fig. 3:**
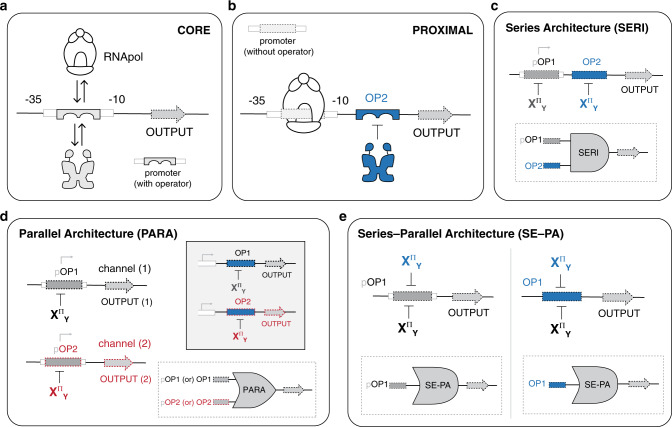
Mechanisms and architectures for directing TFs. **a**, **b** Illustrates the putative mechanisms for single transcription factor interactions at two distinct DNA operator locations, while **c–e** show architectures that facilitate combinatorial logical operations. **a** Core architecture, where the operator is positioned intercalated with (or between) the −35 and −10 hexamers of the bacterial promoter, both upstream of a gene OUTPUT. Mechanistically, the transcription factor and RNA polymerase compete for binding to the promoter-operator region. **b** Proximal architecture. The operator is located between the promoter (beyond the −10 hexamer) and the gene OUTPUT. Here, RNA polymerase and the transcription factor can independently interact with the promoter and operator elements, without competition. **c** Series (SERI) architecture, consisting of one operator each, core and proximal. Transcription factors interact with distinct operators, though the one directed to the core position must compete with RNA polymerase. Note: pOP indicates an operator within a promoter. **d** Parallel (PARA) architecture, with two channels. Each channel consists of a separate operator (core or proximal, inset) controlling a distinct OUTPUT, which could be coupled. **e** Series-parallel (SE-PA) architecture. A single operator, at the core (left) or proximal (right), is regulated by multiple transcription factors via shared DNA-binding domains. This is mechanistically distinct from both the series and parallel architectures, as TFs must work in tandem to regulate gene OUTPUT (i.e., interactions are independent and exchange on a single operator).

In the second stage of our workflow, we bestowed ADR to a fixed set of engineered RCD (E-RCD) (Fig. 2b, Supplementary Fig. 5). To accomplish this, we employed a modular design strategy that we developed in a previous study^25^. Here, the base design space was given by 3 distinct E-RCD and 6 ADR modules; however, each E-RCD had two or more allosteric solutions (Fig. 2b). Accordingly, the total design space is defined by 8 E-RCD and 6 ADR resulting in 48 putative anti-repressors. To test the performance of the putative transcription factors, we utilized two distinct DNA operator positions (core, Fig. 3a; and proximal, Fig. 3b) to direct the independent NOT logical operations (i.e., anti-repressor unit operations). The purpose of evaluating both operator positions separately is that each position achieves gene regulation by way of different mechanisms^47^. The core operator position is intercalated between the −35 and −10 hexamers (Fig. 3a)—thus, TF (repressor or induced anti-repressor) DNA binding competes with the binding of RNA polymerase. Whereas, when the operator is located at the proximal position (Fig. 3b) the architecture can support binding of the TF and RNA polymerase without direct competition. Looking forward, the combination of core and proximal DNA operators results in a series (SERI) architecture (Fig. 3c), which can be used to confer combinatorial logic in biological systems^25^.

**Fig. 4:**
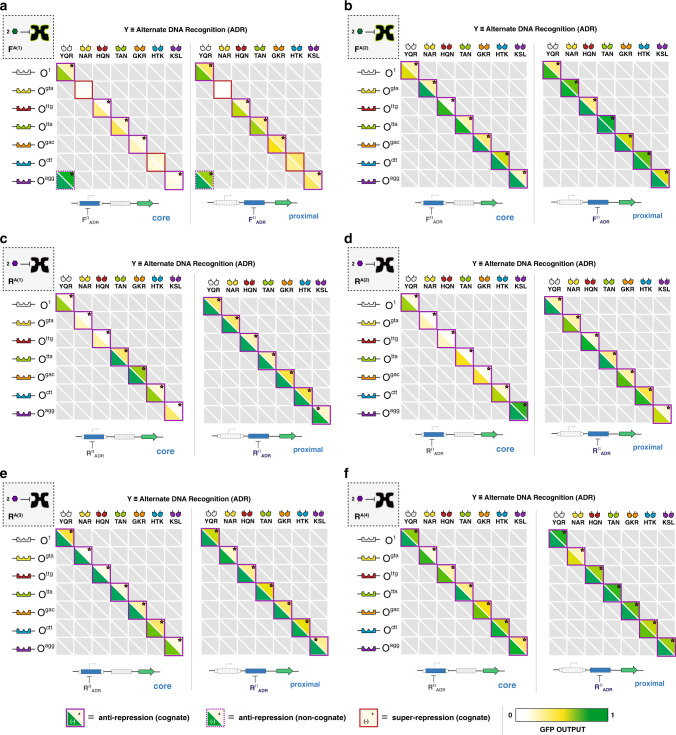
Anti-repression matrices for engineered transcription factors. **a** anti-FruR^(1)^; **b** anti-FruR^(2)^; **c** anti-RbsR^(1)^; **d** anti-RbsR^(2)^; **e** anti-RbsR^(3)^; and **f** anti-RbsR^(4)^. Each square represents an operator-DNA-binding domain pairing. DBDs are across the top of each matrix, with corresponding operators along the left side. The bottom-left sector of each square shows the mean fluorescence intensity (OD_600_ normalized, ex: 485 nm, em: 510 nm) in the absence of the inducer, whereas the top-right sector shows the normalized fluorescence intensity in the presence of the inducer. Shaded colors of sectors correspond to the GFP output scalebar, shown at bottom. All values are standardized to the observed maximum output (measured with a LacI_null_ control plasmid) for a given operator and operator position (core or proximal). Red stars denote a statistically-significant (α = 0.001) difference in fluorescence intensity between the two states using a Student’s two-tailed *t*-test. Solid outlines denote statistically-significant cognate interactions (along the matrix diagonal), whereas dashed outlines denote statistically-significant non-cognate interactions. Purple outlines denote anti-repressor (X^A^) phenotypes and red outlines denote super-repressor (X^S^) phenotypes. Values correspond to the mean of *n* = 6 biological replicates. Any DBD-operator pairing that did not show a super-repressor or anti-repressor phenotype (i.e., were nonfunctional, X^−^) is shown grayed out. See Supplementary Fig. 7 for inverse anti-repression matrices. The left matrix in each set corresponds to testing with the operator in the core position and the right matrix corresponds to testing with the operator in the proximal position.

We initially tested each of the putative transcription factors via the proximal GFP reporter architecture (Fig. 4 (right matrices), also see Supplementary Figs. 4, 5). At this position, we observed that 45 out of 48 of the engineered systems presented an anti-repressor phenotype and interacted with a cognate DNA operator (Fig. 4, Supplementary Figs. 6, 7). Only one anti-repressor unit operation (F^A^_YQR_ | O^agg^) had a non-cognate interaction at the proximal position (Fig. 4a). Unit operations evaluated at the core position resulted in the same fundamental phenotypes; however, larger dynamic ranges were observed in 38% of the NOT unit operations, relative to the proximal BUO (Fig. 4 and Supplementary Figs. 6–8). In 13% of the relative cases the performance at the proximal position was diminished. Namely, (for this subset) the repression strength (i.e., the ON-state, without ligand) had smaller amplitudes resulting in near super-repressive performance (Fig. 4 and Supplementary Fig. 8). Generically, the observed differences between the core and proximal operator positions can be attributed to the aforementioned mechanistic differences illustrated in Fig. 3a, b (Supplementary Note 4). Understanding these differences in performance (individually) will help guide the choice and sequence of BUO when constructing combinatorial logic.

### Biological unit operation metrology

Scientific metrology has been used throughout engineering to standardize the development and quantification of unit operations (or functional parts). Generically, a unit operation represents a fundamental functional object that operates with a high degree of fidelity regardless of the process or location of the given operation—provided the end-user remains within the performance boundary. In principle, BUO can be developed and defined to a similar level of objective performance—provided we have a metrology to define the performance boundary. In our previous study, we established a metrology for 35 (repressor X^+^_ADR_ based) BUFFER unit operations^25^. In brief, this metrology consists of three parts: (i) defining the conditional units of measurement for a given allosteric transcription factor, (ii) reproducible realization of units of measurement at steady-state and ligand saturation, and (iii) development of a traceability score via the comparison of performance metrics for a given transcription factor to a reference system (I^+^_YQR_ | O^1^). Here we seek to extend this metrology to the collection of anti-repressors (i.e., NOT unit operations) established in this study. In turn, we can use this metrology to guide the choice of unit operations that can be used to construct combinatorial logic (and related processes).

**Fig. 5:**
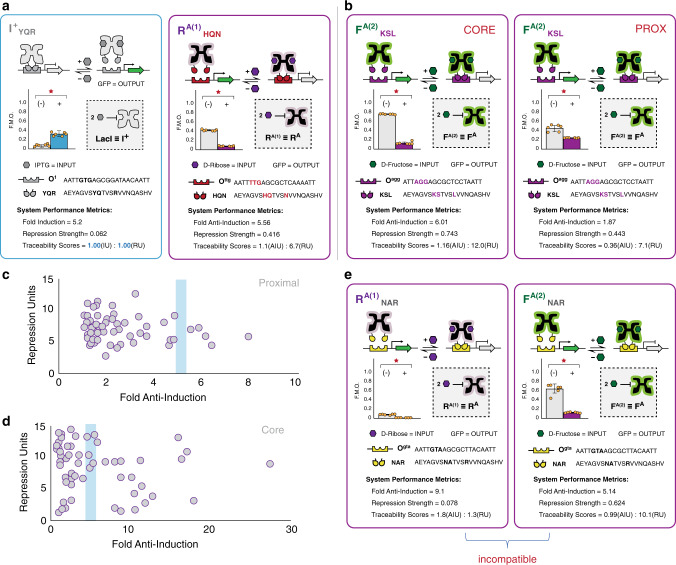
Metrology for engineered anti-repressors. Performance cards can be generated for biological unit operations (BUO), consisting of a regulatory core, DNA-binding domain paired with an operator, at a given position (core or proximal). Vertical-bars display normalized fluorescence intensity with the mean defining the measure of centre (±1 S.D.), standardized to the maximum fluorescence output observed (75,000 r.f.u.), giving fraction of maximum output, F.M.O. Left-bars show values without ligand, whereas the right is value in the presence of ligand. Red stars signify statistical significance (α = 0.001) in a Student’s two-tailed *t*-test for *n* = 6 independent biological replicates with similar results, dots shown for each replicate. Performance metrics can be calculated, see Supplementary Note 4. **a** Criteria for robust synthetic biology tools. Left shows the card for the I^+^_YQR_ | O^1^ (proximal) BUO, the reference for metrology (traceability scores of 1.0 for induction units, IU, and repression units, RU). Right shows the R^A(1)^_HQN_ | O^ttg^ (proximal) BUO, with performance metrics comparable to the reference, indicating the BUO is an acceptable tool for gene regulation in circuits. The I^+^_YQR_ | O^1^ (proximal) BUO is widely-regarded as a standard synthetic biology tool. **b** The effect of operator position on BUO performance. Performance cards for F^A(2)^_KSL_ | O^agg^ in the core (left) and proximal (right) positions. With the operator in the core position, the transcription factor performed better compared to the proximal in terms of dynamic range and repression strength (expression in the ON-state). **c**, **d** Correlation analyses of anti-repressors generated in this work: **c** proximal and **d** core operator positions. Left axis shows repression units and bottom axis, the fold anti-induction (fold change). Each dot represents a BUO engineered in this work. Blue rectangles indicate the fold change of the reference BUO. **e** Incompatible pairing of BUO. Left, R^A(1)^_NAR_ | O^gta(core)^, with anti-induction and repression units in proximity, and *right*, F^A(2)^_NAR_ | O^gta(core)^ performance cards. Disparities in performance metrics, namely fold anti-induction and repression strength, cause these BUO to be incompatible when paired in proposed combinatorial logical operations. R^A(1)^_NAR_ would nullify the performance of F^A(2)^_NAR_ when used together in a gene circuit.

To establish a metrology for the anti-repressor based unit operations we first established a reference system. We selected the LacI wild-type (I^+^_YQR_ | O^1^) unit operation as the reference system—positing that an antithetical (NOT) unit operation with similar (or better) but contrasting performance metrics could be regarded as a robust gene regulator in synthetic biology (Fig. 5a). In the example provided (Fig. 5a), the NOT unit operation (R^A(1)^ | O^ttg^) has similar dynamic range (fold anti-induction) relative to the reference system (I^+^_YQR_ | O^1^). Likewise, this is reflected in the traceability score (anti-induction units, AIU), which is approximately unity. A similar assessment can be conducted to quantify the relative performance for a given TF between operator positions (Fig. 5b)—a complete summary of all of the unit operations developed in this study is provided in Supplementary Fig. 8. In turn, we conducted a correlation analysis to identify all unit operations that performed approximately equal to (or greater than) the reference unit operation (Fig. 5c, d). At the proximal position nine unit operations had a fold anti-induction (dynamic range) greater than or approximately equal to the reference unit operation. Whereas, at the core position 25 unit operations had fold anti-induction values that were on par or greater than the reference system. In most cases unit operations at the core position resulted in larger dynamic ranges. However, two anti-repressors resulted in greater ranges at the proximal position (R^A(2)^_KSL_, and R^A(1)^_TAN_) over the core position, Supplementary Fig. 8.

Notably, the dynamic range has been regarded as the principal performance metric for a given unit operation. However, metrics that can determine compatibility between sets of unit operations should be considered equally important, see Fig. 5e. Toward this end, repression units (RU) provide information regarding the ON state of a NOT unit operation, and the OFF state of a BUFFER unit operation (i.e., the unit operation without the input ligand—in general). Case in point, paired NOT logical operations with extreme differences in RU (e.g., approximately 10-fold or greater) may not be compatible. This is especially true when pairing NOT unit operations where one system has AIU and RU in proximity (i.e., a difference AIU-RU less than ~1.5) with a dynamic range of 5 or greater, Fig. 5e (left card).

Performance metrics were determined via a microwell plate assay, which could result in data convolution due to inherent background signal, especially at low fluorescence levels. This technical limitation could potentially result in erroneous dynamic ranges, specifically in systems that have moderate output in either the repressed or anti-induced state. Accordingly, we conducted single-cell (flow cytometry) experiments on 24 transcription factors at two operator positions (i.e., 48 units in total), sampling a broad (and representative) collection of unit operations (Supplementary Fig. 9). Qualitatively, the NOT unit operation flow cytometry data was in good agreement with the data collected via microwell plate assay with a linear R^2^ value greater than 0.82 (with or without background fluorescence). Quantitatively, all of the unit operations that had the greatest observed difference between the two assays—(R^A1^_KSL_ | O^agg^), (R^A2^_HQN_ | O^ttg^), and (R^A3^_KSL_ | O^agg^)—occurred at the core operator position. This implied that the interference with RNA polymerase binding at the core position could have a greater impact in certain unit operations. However, in general the two-assays were in good agreement. We should note that the dynamic range of a given unit operation can be tuned at the level of the promoter to increase the dynamic range^48^, thus a given BUO with a range lower than 5 could be improved (if need be). Moreover, low-performance unit operations could be of potential use in feedback control systems where moderate to low signal attenuation is needed^49^. For the purpose of this study, we have a sufficient number of unit operations and input signal diversity to demonstrate the utility of this system of TFs as building blocks for combinatorial transcriptional programming.

### The impact of operator position on combinatorial logic

**Fig. 6:**
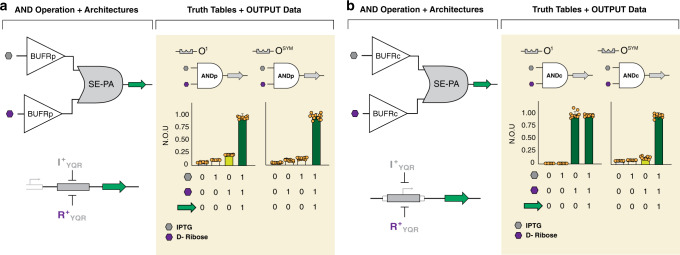
Transcriptional AND gates. AND gates may be constructed using combinations of BUFFER operations, here, highlighting the series-parallel (SE-PA) architecture. In plots, vertical-bars display normalized output units (N.O.U.), the standardized mean (defining the measure of centre) fluorescence intensity as a fraction of the maximum observed for that operation ±1 S.D., and truth tables are shown, below, corresponding to bars, above, for different ligand conditions. Results shown are from *n* = 12 independent biological replicates with similar results, dots shown for each replicate. **a** ANDp gates were constructed from two BUFRp operations. I^+^_YQR_ and R^+^_YQR_ both regulated GFP via a proximal O^1^ (left) or O^SYM^ (*right*) operator. Only in the presence of both ligands, IPTG and D-ribose, was repression from both TFs relieved, allowing transcription. **b** ANDc gates were constructed from two BUFRc operations. Here, I^+^_YQR_ and R^+^_YQR_ both regulated GFP via a core O^1^ (left) or O^SYM^ (right) operator. Results with the O^1^ operator did not show AND behavior, while O^SYM^ allowed realization of the AND operation. See Supplementary Fig 10 for realization of the AND operation using an O^1(core)^ operator.

In principle, we can combine anti-repressor NOT (or repressor, BUFFER) unit operations to build combinatorial logical operations or related complex processes. This workflow is similar to constructing combinatorial logic in electrical circuits via fundamental operations. Toward this end, to guide systems of fundamental biological unit operations (i.e., BUFFER or NOT gates) to form a multiple input logical operation we devised a set of genetic architectures—series, SERI (Fig. 3c); parallel, PARA (Fig. 3d); and series-parallel, SE-PA (Fig. 3e). We used series and parallel in our previous report to construct a broad range of repressor based BUFFER combinatorial operations and related biological programs^25^. Series-parallel was tangentially introduced in a previous report by Shis et al.^43^, and here we formalized this architecture as part of a larger transcriptional programming edifice. In this study, we demonstrated the series-parallel architecture in the context of our programming structure via the development of two AND gates, based on a set of BUFFER unit operations and cognate operator elements (Fig. 6). The series-parallel architecture enabled the interaction of one or more repressor(s) to a common operator, given that the BUFFER unit operations share a common DNA-binding domain. The combination of two BUFFER operations that respond to two disparate signals via series-parallel, resulted in an AND logic gate. Furthermore, this combinatorial logic operation can be directed toward the core or proximal operator element—resulting in different performance metrics (Fig. 6). For example, a series-parallel AND logic gate constructed at the proximal operator (ANDp) site using two signal decoupled BUFFER unit operations (I^+^_YQR_ | O^1^) and (R^+^_YQR_ | O^1^), resulted in the expected AND gated logic (Fig. 6a). However, when the same set of BUFFER unit operations were evaluated with the same O^1^ DNA element located at the core position, the BUFFER operation utilizing the I^+^_YQR_ repressor was nullified, thus reduced the system to a single input operation (Fig. 6b). Given that Shis et al. demonstrated that LacI cannot be induced via D-ribose^43^, we posited that the annulled performance of the BUFFER requiring isopropyl β-d-1-thiogalactopyranoside (IPTG) as the input signal (oriented toward the core DNA element), was the result of reduced binding affinity between the repressor (I^+^_YQR_) and the DNA operator (O^1^) with RNA polymerase acting as an antagonist, Fig. 3a. Accordingly, we surmised that (i) increased operator-binding affinity of repressor I^+^_YQR_ via an alternate BUFFER (I^+^_YQR_ | O^SYM^) unit operation (Fig. 6b), or (ii) increased production of I^+^_YQR_ via promoter tuning (Supplementary Fig. 10), would restore ANDc performance.

### Next-generation NOR logic gates

In contemporary genetic programing, biological NOT logic gating is achieved by way of an inverter, requiring the use of two promoters (i.e., an input promoter (P_IN_) and output promoter (P_OUT_)), in addition to a pair of non-synonymous DNA-binding proteins (see Supplementary Fig. 11a)^26,38^. Given that a variety of gene regulators can be used to regulate the input promoter (P_IN_)—though achieved via a broad range of topologies and functional mechanisms—different input signals can be employed. Accordingly, two-signal NOR gates can be created^26^, provided that a pair of compatible input promoters (P_IN_) can be constructed—thus, bringing the total number of promoters for this variety of NOR logic gate to three (see Supplementary Fig. 11b, c). The series-parallel architecture can also be used to construct NOR logic gates, analogous to the approach used to build AND logical gates (Fig. 6). However, instead of sets of BUFFER unit operations, a putative NOR gate would utilize sets of NOT unit operations, Supplementary Fig. 11e. Using this edifice and workflow would greatly simplify the construction of NOR logic gates, only requiring the use of a single promoter.

**Fig. 7:**
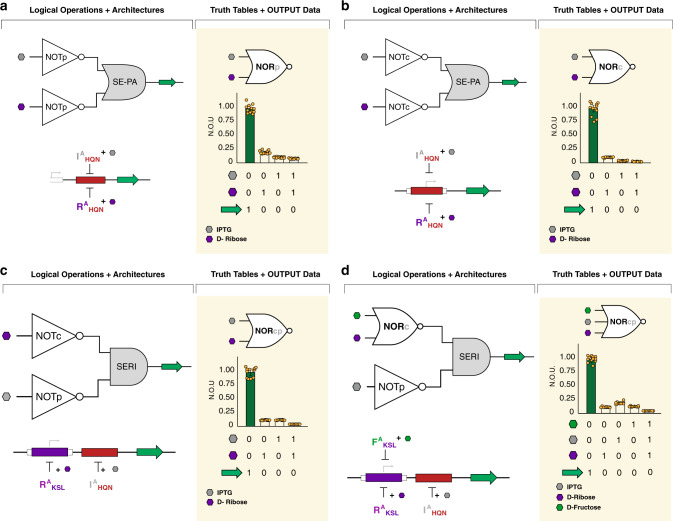
Transcriptional NOR logic gates. NOR gates may be constructed using combinations of NOT operations in either series-parallel (SE-PA) (**a**, **b**) or series (SERI) (**c**, **d**) genetic architectures. In plots, vertical-bars display normalized output units (N.O.U.), the standardized mean (defining the measure of centre) fluorescence intensity as a fraction of the maximum observed for that operation ±1 S.D., and truth tables are shown, below, corresponding to bars, above, for different ligand conditions. Results shown are from *n* = 12 independent biological replicates with similar results, dots shown for each replicate. **a** NORp gates were constructed from two NOTp operations in SE-PA. R^A(1)^_HQN_ and I^A(5)^_HQN_ (from Rondon and Wilson^44^) both regulated GFP via a proximal O^ttg^ operator. In the presence of either, or both, ligands, IPTG and D-ribose, transcription was anti-induced, causing diminished gene expression. **b** By moving the O^ttg^ operator to the core position, a NORc gate was constructed from two NOTc operations. **c** A NORcp gate was constructed from two NOT operations in series—NOTc and NOTp. Here, R^A(1)^_KSL_ regulated a core O^agg^ operator and I^A(5)^ independently regulated a proximal O^ttg^ operator, both upstream of the GFP reporter gene. Only in the presence of neither ligand was gene transcription unabated. **d** A three-input NORcp gate was constructed by the addition of the F^A(2)^_KSL_ transcription factor, which additionally regulated the core O^agg^ operator. Now, the presence of any of the three ligands, IPTG, D-ribose, and fructose, was sufficient to inhibit gene expression.

Toward this end, we demonstrated NOR construction via series-parallel pairing of two disparate NOT unit operations, that share a common DNA-binding domain (Fig. 7a, Supplementary Fig. 12). In this initial example, a two-signal NOR gate directed toward the proximal operator position (NORp) was constructed. Specifically, the DNA operator O^ttg^ was positioned in the proximal configuration, and we deployed two distinct non-natural anti-repressors (I^A^_HQN_ and R^A^_HQN_). Objectively, this two-signal NOR gate could not produce an output signal (GFP) when either (or both) input signal(s)—IPTG or (and) D-ribose—were present (Fig. 7a). In turn, we constructed a second iteration NOR gate via the core configuration (NORc) using the same pair of anti-repressors (Fig. 7b and Supplementary Fig. 12). The NOR logic gate located at the core position (NORc) performed via broader sets of dynamic ranges between the ON and OFF states, relative to the proximal (NORp) operation. We can reconcile the performance of the NORc gate by attributing the reduced leakiness of the OFF states (relative to the NORp) to greater interference with RNA polymerase binding, upon anti-repressor induction, see Fig. 3a.

In addition to series-parallel directed NOR gates, we also constructed an alternate two-input combinatorial logic operation via the series architecture (Fig. 7c and Supplementary Fig. 12). This series directed NOR used two disparate DNA operators, one at the core position (O^agg^) and the other located at the proximal position (O^ttg^). Discretely, each operator can be objectively described as independent NOT unit operations—i.e., core (NOTc) and proximal (NOTp). Using an architecture enabling discrete unit operations for information processing allows for independent tuning of each constituent NOT logic function. Moreover, the series architecture can also be used to construct more elaborate combinatorial logic. We illustrated this via the construction of a three signal NOR gate (Fig. 7d and Supplementary Fig. 12). Objectively, the three signal NOR gate was represented by a two signal series-parallel directed core operator position (forming a two-signal NORc gate—i.e., input ligands fructose and ribose), coupled in series with a NOT at the proximal position (NOTp) that was responsive to a third signal IPTG.

### Constructing NAND and XNOR logic gates

**Fig. 8:**
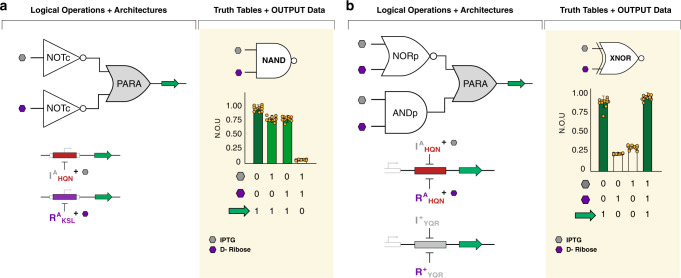
Transcriptional NAND and XNOR gates. Combinatorial logic in the parallel (PARA) architecture allows the construction of the NAND and XNOR operations. In plots, vertical-bars display normalized output units (N.O.U.), the standardized mean fluorescence intensity (defining the measure of centre) as a fraction of the maximum observed for that operation ±1 S.D., and truth tables are shown (below) corresponding to bars (above) for different ligand conditions. Results shown are from *n* = 12 independent biological replicates with similar results, dots shown for each replicate. **a** The NAND operation was achieved with two NOTc operations in PARA. In one channel, I^A(9)^_HQN_ (from Rondon and Wilson^44^) regulated GFP via a core O^ttg^ operator; in a second channel, R^A(1)^_KSL_ regulated GFP via a core O^agg^ operator. In the presence of neither ligand, transcription was fully-permitted in both channels (relative OUTPUT = 1), while in the presence of only one ligand, IPTG or D-ribose, one channel was anti-induced, but, in the other, transcription was fully-permitted (relative OUTPUT ~ 1). Only in the presence of both ligands were both channels anti-induced, leading to full inhibition of gene expression. **b** The XNOR operation was constructed with a NORp and ANDp operation in PARA. In one channel, I^A(9)^_HQN_ and R^A(1)^_HQN_ simultaneously regulated GFP via a proximal O^ttg^ operator in a SE-PA architecture; in the second channel, I^+^_YQR_ and R^+^_YQR_ regulated GFP via a proximal O^SYM^ operator (see Fig. 6b for this individual BUO). In the presence of either no ligand present, or both ligands present, one channel fully-permitted transcription, while the other was attenuated, leading to relative OUTPUT = 1. In the presence of only one ligand, however, both channels were attenuated, as simultaneously, either I^+^_YQR_ and R^A(1)^_HQN_ or R^+^_YQR_ and I^A(9)^_HQN_ were interacting with their respective operators, causing relative gene output to be diminished.

Finally, we used the parallel architecture (Fig. 3d) to construct combinatorial logic that used both anti-repressor (NOT) and repressor (BUFFER) unit operations. The first logical operation that we constructed via the parallel architecture was a NAND gate (Fig. 8a and Supplementary Fig. 13). In this system, we used two different NOT unit operations, each regulating a separate channel with the same output (GFP). When both input signals were present (IPTG + D-ribose) the production of GFP was attenuated on both channels. However, when no input signals were present the combinatorial operation produced GFP at a relative value of one, as neither channel was anti-induced. Likewise, when only one signal was present, only one channel was anti-induced, enabling the maintained expression of GFP at an approximate value of (1). In the second iteration, we used the parallel architecture to construct an XNOR gate (Fig. 8b and Supplementary Fig. 13). In this two-channel system, we used two signal paired unit operations—i.e., where signal-coupling was achieved via a BUFFER and the antithetical NOT. In the first channel we constructed a two-signal NOR gate at the proximal position (NORp), directing a set of anti-repressors via a series-parallel architecture, regulating the production of GFP. We complemented the NOR channel with an ANDp (series-parallel) gate constructed on a separate channel. The AND channel was two-signal coupled to the NOR channel, such that both channels produced the same GFP output. Accordingly, in the absence of input signals the NOR channel produced GFP at the maximum normalized output unit value (approximately one), whereas the AND channel was repressed. In contrast, when both signals were present the AND channel had an output of approximately one—while the NOR channel was maximally anti-induced. In cases where only one signal was introduced, both channels were attenuated (i.e., the normalized output units were approximately zero). Explicitly, when IPTG was the only signal present the NOR channel was anti-repressed via the I^A^_HQN_ transcription factor, while the AND channel was repressed via R^+^_YQR_.

## Discussion

In this study we introduced a workflow for engineering non-natural anti-repressors, and used this collection of inducible unit operations to expand our current transcriptional programming language. Engineering transcriptional anti-repressors required us to confer alternate allosteric communication in two distinct regulatory cores that are predicted to share the LacI structural topology. We posited that allosteric mechanisms in the FruR and RbsR RCD scaffolds were similar; thus, alternate communication could be conferred using analogous workflows. While the majority of studies of allostery have focused on understanding the requirements for communication (i.e., the medium), our study has highlighted the role of functional surfaces that facilitate DNA binding. Specifically, we observed that alternate DNA binding can influence allosteric outcomes (Fig. 4). For example, the E-RCD derived from FruR with variation in ADR resulted in cognate anti-repression, super-repression, or non-cognate anti-repression (Fig. 4a, b). Based on this observation, we re-evaluated the F^A^_YQR_ and R^A^_YQR_ E-RCD using the respective wild-type DNA-binding domains (Supplementary Fig. 14). Both FruR E-RCD adapted with the wild-type DNA-binding domain changed from an anti-repressor (F^A(1)^_YQR_ and F^A(2)^_YQR_) to the repressor (F^+(1)^_WT_ and F^+(2)^_WT_) phenotype. Likewise, 3 out of 4 RbsR E-RCD reverted to non-anti-repressor phenotypes. Accordingly, we hypothesized that the initial discovery of active alternate allosteric networks that confer anti-repression can be influenced by functional surface feedback. What is clear from the case studies presented here is that the full a priori design of a functional allosteric protein will require the simultaneous design of both functional surfaces along with the corresponding allosteric topology.

**Fig. 9:**
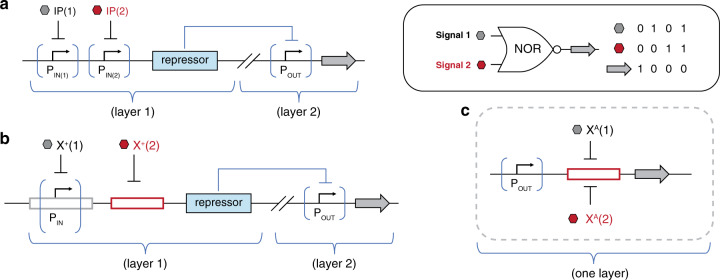
Simplification of a transcriptional NOR gate over three generations. **a** In the first generation, Tasmir et al.^26^, constructed a NOR operation using two layers with three promoters. P_IN(1)_ and P_IN(2)_ regulate expression of an uninduced repressor, which, in turn, regulates expression of OUTPUT from a third promoter, P_OUT_. In the presence of any, or both, ligands corresponding to P_IN(1)_ and P_IN(2)_, the repressor is expressed, which inhibits P_OUT_. **b** In the second generation, our previous report^25^, constructed a NOR operation using two layers with two promoters. P_IN_ drives expression of a repressor, which is additionally regulated by an operator (red box). Regulation is achieved with X^+^ repressors. A third, uninduced repressor then regulates expression of OUTPUT via P_OUT_. **c** In the third generation, we have reduced the functionally-complete NOR operation to a single promoter in one layer using engineered anti-repressors. OUTPUT, driven by P_OUT_, is directly regulated by two anti-repressors using one operator (here, shown in the proximal position). Only in the presence of neither ligand is the OUTPUT not anti-induced by the two X^A^ transcription factors.

In turn, we leveraged our system of non-natural transcription factors, to demonstrate the range of capabilities with regard to biological programming via inducible anti-repression. One advantage of using engineered anti-repressors over repressors was demonstrated in the development of NOR logic gates (Fig. 7). In our previous study^25^, we developed an apparent NOR gate using three disparate repressors via a two layer architecture (Fig. 9b). In this iteration, the NOR gate’s first layer utilized a set of BUFFER unit operations (forming an AND logic gate) that regulated an un-induced repressor (by way of an input promoter (P_IN_))—objectively serving as an inverter. In turn, the repressor fed into an output promoter (P_OUT_) controlling a single GFP output. Relative to the state-of-the-art^26^ (Fig. 9a), this combinatorial NOR operation reduced the number of promoters from three to two. In this study, we developed and employed engineered anti-repressors to achieve the next-generation of NOR logic, which allowed us to vastly simplify the design of this circuit further—i.e., to a single promoter system, and utilizing one fewer regulatory proteins (Fig. 9c). The ability to build simplified NOR logical operations of this sort will enable the development of more complex transcriptional programs, as these processes will require fewer unit operations. In addition, leveraging the construction of these programs at the core position can mitigate issues related to resource partitioning of endogenous RNA polymerase. Namely, anti-repression at the core position displaces RNA polymerase binding. Thus, anti-repressor mediated displacement of RNA polymerase increases the number of RNAP in the free state—thus reducing one aspect of metabolic burden. Moreover, unit operations constructed from inducible anti-repressors at the core position can assuage performance issues that stem from competitive binding when using repressors (Fig. 6). Collectively this study illustrates the utility and advantages of using inducible anti-repressors in transcriptional programming. Finally, an additional motivation for developing simplified NOR operations (Fig. 7), is that this particular operation is functionally complete—i.e., any computational operation can be implemented by layering NOR gates alone^26^.

This system of inducible anti-repressors represents a nascent NOT based transcriptional programming language, that is complementary to our repressor (BUFFER) based unit operations. Moreover, the workflow presented in this study has a putative design space that exceeds ~10^5^ non-synonymous engineered anti-repressors. Namely, more than 1000 regulatory core share the same topology as the RCD modules used in this study^50^, combined with ~200 non-natural alternate DNA-binding domains^51^ that can be paired with each RCD—defining the larger putative design space. In addition, engineering of a given RCD toward anti-repression can potentially generate a variety of dynamic ranges in the same scaffold^40^. Accordingly, the combination of existing and putative anti-repressors, repressors, and genetic architectures provides a powerful and scalable transcriptional programming foundation that can be utilized toward general programmed decision making in biology.

## Methods

### Strains, plasmids, media

All assay experiments were performed in the *Escherichia coli* strain 3.32 (*lacZ13(Oc) lacI22 λ*^*−*^ *el4- relA1 spoT thiE1;* Yale CGSC #5237), transformed chemically, while standard DNA cloning was performed in NEB 5-alpha Competent *E. coli* (*huA2 Δ(argF*^*−*^*lacZ)U169 phoA glnV44 φ80Δ(lacZ)M15 gyrA96 recA1 relA1 endA1 thi-1 hsdR17*; New England Biolabs) and library construction was performed in electrocompetent *E. coli* 3.32 cells. Cells were grown in LB Miller Medium (Fisher Scientific) or M9 Minimal Medium (6.8 g L^−1^ Na_2_HPO_4_, 3.0 g L^−1^ KH_2_PO_4_, 0.5 g L^−1^ NaCl, 1.0 g L^−1^ NH_4_Cl, 2 mM MgSO_4_, 100 μM CaCl_2_; Millipore Sigma) supplemented with 0.2% (w/v) casamino acids (VWR Life Sciences), 1 mM thiamine HCl (Alfa Aesar). LB Miller + Agar (Fisher Scientific) was used for selection when cloning. Antibiotics and ligands were used as appropriate. Antibiotics used were: chloramphenicol (25 μg mL^−1^; VWR Life Sciences), kanamycin (35 μg mL^−1^; VWR Life Sciences), and carbenicillin (100 μg mL^−1^; Teknova). Ligands used were: fructose (Arcos Organics), d-ribose (Arcos Organics), and isopropyl-β-d-thiogalactoside (IPTG; Millipore Sigma). All ligands in this study were used at a concentration of 10 mM.

### Cloning and transcription factor plasmid construction

For all cloning experiments, oligo synthesis and DNA sequencing were performed by Eurofins Genomics. All DNA constructs (genetic architectures and protein mutants) were sequenced to verify mutations, sequence identity, and correct assembly following transformation and plasmid isolation (Omega Bio-Tek). Sequence alignments and primer design were aided by ApE Plasmid Editor and SnapGene software. All polymerase chain reactions (PCR) were performed using Phusion High-Fidelity PCR Master Mix with GC Buffer (NEB), except for the directed evolution library, which used *Taq* DNA Polymerase (NEB). Plasmids with chimeric transcription factors in the pLacI (Novagen) backbone (featuring a low copy number p15A origin, chloramphenicol resistance marker, and the constitutive LacI promoter driving a transcription factor gene) were taken from Rondon, et al.^25^. Briefly, these were constructed by obtaining the respective open reading frames (ORFs), RbsR-L (Addgene #60773), and FruR-L (Addgene #60768), gifts from the Swint-Kruse and Bennett Labs, and inserting them into pLacI using circular polymerase extension cloning (CPEC)^52^ following amplification of the genes. Likewise, pLacI vectors with wild-type FruR (UniProt # P0ACP1) and RbsR (UniProt #P0ACQ0) ORFs were constructed by inserting a genomic copy (guided by Blattner et al.^53^) of the ORFs into pLacI using CPEC following amplification of the gene via colony PCR from *E. coli* 3.32 cells. As done by Rondon et al.^25^, FruR-L was driven by the LacI^q^ (rather than LacI) promoter^54^, which leads to approximately a ten-fold increase in protein production. This was performed with inverse PCR followed by treatment with KLD Enzyme Mix (NEB).

### Reporter system and series-parallel genetic architecture

The GFP reporter plasmid system developed by Rondon and Wilson^44^ was utilized for the single TF studies with a proximal operator and was also used as the series-parallel proximal architecture. This construction began with the pZS*22-sfGFP developed by Richards et al.^40^ (featuring a pSC101* origin, a kanamycin resistance marker, and *lac* O^1+^-regulated sfGFP reporter gene). The plasmid was linearized, excluding the promoter and operator elements, and a small fragment containing the constitutive *trc* promoter element^55^ (a hybrid of *trp* and *lac* UV5 promoters), a spacer sequence, and the operator sequence, was constructed via oligos, was inserted via CPEC. Each individual operator variant reporter was constructed via site-directed mutagenesis, beginning with the original proximal reporter plasmid (with the O^1^ operator). The wild-type *rbsDACBK* operator, identified by Shimada et al.^56^, was cloned into the engineered O^1^-regulated proximal reporter plasmid via site-directed mutagenesis. The core operator reporter system was adapted from the proximal. This was utilized for single TF studies with a core operator and was also used as the series-parallel core architecture. Using inverse PCR, the segment including the spacer sequence and operator was deleted, then site-directed mutagenesis was used to insert 17-bp of an operator sequence in the region between the −35 (TTGACA) and −10 (TATAAT) boxes of the *trc* promoter (similar to the original pLLac-O1 architecture). As with the proximal operator system, each individual operator variant reporter was constructed via site-directed mutagenesis, beginning with the original (which contained an O^1^ operator in the core position). The wild-type *fruBKA* operator, identified by Ramseier et al.^57^, was cloned into the engineered O^1^-regulated core reporter plasmid via site-directed mutagenesis. Attempts to assay with the *fruBKA* operator in the proximal position, as done with the *rbsDACBK* operator, did not show any repressibility or inducibility in assays with cells co-transformed with wild-type FruR.

### Transcription factor vectors

To construct plasmids with multiple TF ORFs for use in genetic logic gates, additional plasmids were developed, as introduced by Rondon et al.^25^. All plasmids featured orthogonal origins of replications and selection markers. The first utilized the pLacI architecture. The pLacI plasmid was first linearized, visualized, and gel extracted (Qiagen), along with a fragment containing an amplified, second TF ORF, including a second constitutive LacI promoter. The plasmid was then assembled using the NEBuilder HiFi Kit (NEB). The constructs utilizing this architecture included the R^A(1)^/I^A(5)^ and R^A(1)^/I^A(9)^ dual-TF plasmids. A second TF vehicle plasmid was developed from the pSO vector generated in Rondon and Wilson^44^. This plasmid contains the PBR322 origin, an ampicillin (or carbenicillin) resistance marker, and a TF ORF driven by the constitutive LacI promoter. The original pSO vector was constructed by amplifying the AmpR coding region from pLS1 (Addgene #31490), then visualizing the gel extracting the fragment. This was then combined with the LacI (I^+^) ORF, amplified from pLacI, via splicing by overlap extension (SOE). Finally, the PBR322 origin was PCR amplified from the pET-28b vector (a gift from the Kane Lab), visualized, and gel extracted, before being combined with the SOE fragment via CPEC. To insert a second TF ORF into pSO, the vector was first linearized and the coding region for a second TF ORF was amplified. Both fragments were gel extracted, then the plasmid was assembled using the NEBuilder HiFi Kit (NEB). The constructs using this architecture included the R^+^/I^+^ dual-TF plasmid, including the variant with I^+^ under the LacI^q^ promoter, and the F^A(2)^/I^A(5)^ dual-TF plasmid. Modification of the promoter was performed on pSO prior to the insertion of a second TF, so as not to mutate the identical LacI promoter elements.

### Genetic architectures

Two additional types of genetic architectures—series and parallel—were constructed for genetic logic gates. Briefly, series architectures consist of two sequential operators, one in the core position and one in the proximal, as defined by Cox et al.^47^, controlling one gene. Parallel architectures consist of two operators, in either the core or proximal positions, controlling two distinct ORFs. Both were adapted from Rondon et al.^25^, using the master architecture as parallel, with an orthogonal operator sequence in the unused position. For the series architecture, the starting point was the pZS*22-sfGFP plasmid. The plasmid, excluding the promoter and operator, was PCR amplified in two fragments, which were then visualized and gel extracted (Qiagen). The region upstream of the sfGFP gene containing the promoter element, operators, and the synthetic insulator RiboJ^58^ was synthesized via oligos. The *trc* promoter was used as a scaffold, but the region between the −35 and −10 boxes were replaced with 17-bp of an operator sequence, similar to the core architecture, above. The second operator was introduced in the proximal position 15-bp downstream of the end of the −10 box. This synthesized region was combined with the vector fragments by sequential SOE and CPEC reactions, resulting in a complete plasmid.

For the parallel architecture, the starting point was the series plasmid, described above. First, the plasmid was linearized, and this fragment was visualized and gel extracted. Next, the region upstream of the second copy of sfGFP was synthesized via oligos. In this case, we used the strong pL promoter element as the scaffold and replaced the region between the −35 (TTGACA) and −10 (GATACT) with 17-bp of an operator sequence. Again, the second operator was introduced in the proximal position, 15-bp downstream of the end of the −10 box. Another genetic insulator part was employed (this time, RiboJ10^18^, to introduce sequence diversity). The second copy of sfGFP (along with the strong terminator rrnb1) was amplified from the original pZS*22sfGFP, which was visualized and gel extracted, before being combined with the synthesized region via SOE. Finally, the linearized series architecture plasmid was combined with this newly synthesized fragment using the NEBuilder HiFi Kit. In both architectures, self-cleaving ribozyme genetic insulators were used to eliminate transfer function variability caused by differences in operator and 5′ UTR sequence^25^. When operator sequences needed to be altered to construct different logical operations, this was done using site-directed mutagenesis using inverse PCR followed by KLD enzyme treatment (NEB). The −10 box for the pL promoter element (GATACT) was mutated to the sequence GACTAT (from iGEM J23116) in the XNORp gate to account for difference in the OFFstates of repressors and anti-repressors. This was also done using inverse PCR, followed by KLD treatment.

### Sequence alignment

Pairwise alignment was performed using the EMBL-EBI EMBOSS Needle online tool, with protein sequences from the UniProt database. Default settings were selected, with the matrix EBLOSUM62 and the gap and extend penalties set to 10.0 and 0.5, respectively. Multiple sequence alignment was similarly performed using the EMBL-EBI Clustal Omega web server, with protein sequences from the UniProt database (or, in the case of RbsR-L, Addgene #60773, and FruR-L, Addgene #60768).

### Protein library creation

All site-saturation mutagenesis was performed using a protocol using inverse PCR with NNS degenerate codons. Following treatment with KLD Enzyme Mix (NEB), product was transformed into NEB 5-alpha Competent Cells. All transformants (i.e., the library) from selection plates were streaked and cultured for plasmid isolation and the DNA was sequenced to verify site-saturation coverage at the desired site. For mutagenesis to validate the transfer of allosteric networks between scaffolds, site-directed mutagenesis via inverse PCR was performed with primers to incorporate a single residue at a site (following screening to determine a desired residue) or, for incorporation of multiple mutations across the RCD, the pLacI vector and the fragment containing the mutant RCD were amplified, then combined via CPEC reaction.

The directed evolution library was constructed using a protocol adapted from Meyers et al.^59^. In one PCR, the pFruR-L vector was linearized excluding the region corresponding to the RCD (residue 63 through 334). While there is potential for mutation in the DBD or hinge region to contribute to allosteric inversion, we chose to constrain mutagenesis to the allosteric core, as done previously, in successful studies^40,59^. In another PCR, the FruR-L RCD region was subjected to error-prone PCR. A master library with 5- to 7-bp (3–5 residue) mutations, on average, over the 815-bp region (~0.7% error frequency), was constructed in a reaction with 1.25 U *Taq* DNA Polymerase (NEB), 1X *Taq* Mg-free buffer (NEB), 1.8 mM MgCl_2_ (NEB), 200 μM MnCl_2_ (Millipore Sigma), 0.4 μM dCTP (NEB), 0.4 μM dTTP (NEB), 0.08 μM dGTP (NEB), 0.08 μM dATP (NEB), 500 μM, each, forward and reverse primers, and 10 ng (4.2 fmol) of pFruR-L DNA, as template. The reaction was subjected to 95 °C for 3 min and 20 cycles of 95 °C for 30 s, followed by 68 °C for 5 min, and a final extension at 68 °C for 10 min. The vector and error-prone RCD insert were both visualized on gels, then extracted (Qiagen). The two fragments were combined via CPEC, then transformed into electrocompetent 3.32 cells. The library size was estimated to be on the order of 10^7^ colony forming units (cfu). As above, all transformants were streaked from plates and plasmids were isolated. Libraries for screening were developed by performing site-directed mutagenesis on the master library to introduce the F^S^ super-repressor mutations (namely, L95K, L95I, I96R, I96P).

### Transformation into reporter cell line

All screening experiments were done in the 3.32 cell line. Isolated DNA constructs bearing TFs (on pSO or pLacI scaffolds) were co-transformed with isolated reporter plasmids and selected for on LB Agar plates. For site-saturation libraries, 96 colonies were picked and inoculated into LB with appropriate antibiotics and grown overnight – 96 were picked for chimera site-saturations, giving a 95% probability of all 32 codons (NNS; 4x4x2) were represented in the library.All transformants were cultured in LB (with antibiotics) overnight, at 37 °C shaking at 300 rpm, in preparation for screening. All screening for discovery was done using the O^1^ operator in the proximal position and the wild-type LacI DBD (YQR) on the TF.

### Microplate assay

The microplate assay protocol was taken from Richards et al.^40^. Briefly, colonies were inoculated in LB with relevant antibiotics (chloramphenicol for pLacI, kanamycin for the GFP reporter, carbenicillin for pSO) and grown overnight at 37 °C, shaking at 300 rpm. Cultures were then diluted 1:100 in supplemented M9 Minimal Media containing relevant antibiotics and ligands, as called for, in the wells of a 96-well, sterile, conical-bottomed microwell plate (Nunc). For single TF assaying, trials (variant + condition) were inoculated in replicates of 6, while for logic gates, replicates of 12 were used. Microplates were sealed with Breathe-Easier membranes (Midwest Scientific) to prevent evaporation. Microplates were grown at 37 °C, shaking at 300 rpm, for 20 h. Cultures were then transferred to the wells of a 96-well black-sided, clear bottomed assay plate (Costar). Fluorescence (characteristic to sfGFP—excitation: 485 nm, emission: 510 nm) and optical density (OD_600_) were measured by plate reader (Molecular Devices SpectraMax M2e). Data was collected with SoftMax Pro Software (Molecular Devices). Fluorescence intensity was normalized to cell density. Maximum fluorescence for all operator systems was measured by assaying a *lac*_null_ control, which is a variant in the pLacI vector with STOP codons at positions 2 and 3 of the LacI reading frame^40^. Hence, this plasmid produces no TF while exerting similar metabolic burden. This value was used for standardization across variants with the same operator. For logic gate assaying, all values were normalized to the highest value across all conditions. An averaged value for blank optical density and fluorescence was subtracted from all measured samples. Data analysis was performed in Microsoft Excel.

### Conferring alternate DNA recognition

DBDs for each TF were mutated to ADR modules using inverse PCR followed by KLD enzyme treatment (NEB). Codons corresponding to positions Y17, Q18, and R22 on LacI were mutated to the respective 6 other DBD modules.

### Cell cytometry analysis and sorting

Cultures were prepared similarly to those prepared for the microplate assay, and as done by Richards et al.^40^. Briefly, colonies were inoculated in LB with relevant antibiotics (chloramphenicol for pLacI, kanamycin for the GFP reporter, carbenicillin for pSO) and grown overnight at 37 °C, shaking at 300 rpm. Cultures were then diluted 1:100 in supplemented M9 Minimal Media containing relevant antibiotics and ligands, as called for, in sterile culture tubes (Nunc) and grown at 37 °C, shaking at 300 rpm, for 20 h. Each culture was then aliquoted such that the optical density (OD_600_) would be approximately equal to 0.2 (WPA Biowave CO8000 Cell Density Meter) in the final solution volume (typically 1 mL). Cells were then pelleted at 17,500 *g* for 2 min (Beckman Coulter Microfuge 18). The supernatant was then discarded, and cells were resuspended in PBS supplemented with 25 mM HEPES (Fisher Scientific), 1 mM EDTA (Millipore Sigma), and 0.01% (v/v) Tween20 (VWR Life Sciences), and again pelleted at 17,500 × *g* for 3 min. This wash step was repeated once before cells were finally resuspended in PBS supplemented with 25 mM HEPES and 1 mM EDTA.

Cytometry experiments were performed using a BD FACSAria Fusion flow cytometer (BD Biosciences) equipped with a 100 mW 488 nm laser for excitation, a 510/30 nm bandpass emission filter, and an 85 µm nozzle and data was collected using BD FACSDiva (BD Biosciences) software. Cells were interrogated measuring FITC-Area at flow rates between 10 and 30 and µL min^−1^. Events were gated on forward- and side-scatter and a threshold were set by side-scatter (5000), with doublets discriminated against using standard FSC-Area vs. -Height and SSC-Area vs. -Height plots. At least 25,000 events were recorded for cytometry analysis, while directed evolution libraries screened 20,000–80,000 events per sort. For directed evolution screening, cells were sorted directly into LB (with antibiotic), then cultured overnight at 37 °C, shaking at 300 rpm, in culture tubes (Nunc). This culture was then used to inoculate for another day of sorting after being prepared, as above. Sorts for F^A^ phenotype consisted of two sequential sort steps: the first with the ligand, collecting the low-fluorescence mutants (F^S^ and F^A^, screening out residual wild-type F^+^ and non-functional F^−^), the second without ligand, collecting the high-fluorescence mutants (discriminating between F^S^ and F^A^). Gates for low and high fluorescence were determined from controls with known cytometry performance—i.e., *lac*_null_, parent F^S^, and I^A^ and I^S^ variants from Richards et al.^40^. For the settings utilized, the high-fluorescence mutants were binned ≥10^4^ FITC-A, while low-fluorescence mutants binned <10^4^ FITC-A. Each individual sort step was repeated once to enrich the desired population and filter out false positives. Data was analyzed using the BD FlowJo (BD Biosciences) software package. See Supplementary Fig. 15 for gating and sorting strategies. Following the final sorts, cells were grown overnight at 37 °C, shaking at 300 rpm in LB with antibiotics, then plated on selection plates. Once grown overnight at 37 °C, individual colonies were screened via the microplate assay to verify phenotype and determine performance characteristics.

### Statistical analyses

Phenotypes were evaluated by performing a two-tailed Student’s *t*-test, allowing for unequal variances, between the induced and uninduced states. The significance level was set to α = 0.001. Variants that saw no statistically-significant difference between the induced and uninduced states were X^S^ and X^−^ variants. Classification depended upon the magnitude of fluorescence output. If the fluorescence intensity was greater than 50% of the maximum for that operator variant, it was classified as X^−^; if less than 50% of the maximum, it was classified as an X^S^. For logic gates, a one-way ANOVA followed by a post-hoc Tukey HSD test was performed on the fluorescence outputs under the different ligand treatments to determine the statistical difference between groups. The significance level for logic gates was set to α = 0.01. For both phenotype evaluation and logic gates, Cohen’s *d* tests were performed to determine effects of sample size. For all cases, plots represent averages of biological replicates, with error bars representing ±1 standard deviation. See Source data File for *p*-values, effect size test parameters, and Tukey HSD test results.

### Reporting summary

Further information on research design is available in the Nature Research Reporting Summary linked to this article.

## Supplementary information

Supplementary Information

Reporting Summary

## Data Availability

The authors declare that all data supporting the findings of this study are available within the paper and its Supplementary information. The analyzed data and Source data files will be made available in Supplementary data Files and Source data. Any other information can be made available from the corresponding author upon reasonable request. The sequences of the following plasmids are provided in GenBank: Proximal Reporter Plasmids (MN207964–MN207971), Core Reporter Plasmids (MT127263–MT127272), anti-FruR Plasmids (MT127340–MT127357), anti-RbsR Plasmids (MT127280–MT127308), wild-type FruR Plasmids (MT127333–MT127339), wild-type RbsR Plasmids (MT127309–MT127314), F^+^_YQR_ Plasmid (MN207916), R^+^_YQR_ Plasmid (MN207958), anti-LacI Plasmids (MT127315 - MT127332), dual-TF Plasmids (MT127275–MT127279), pLacI (MT127274), pLacNULL Plasmid (MT127273), pXNOR (MT127262), pNORcp (MT127261), and pNAND (MT127260). Protein sequences for wild-type LacI, RbsR, and FruR proteins can be found in the UnitProt database with accession numbers: FruR (#P0ACP1), RbsR (#P0ACQ0), and LacI (#P03023). Source data are provided with this paper.

## Source data

Source Data
