# Supplementary material for: Engineered systems of inducible anti-repressors for the next generation of biological programming
Source: Nat Commun. 2020 Sep 7;11:4440. doi: 10.1038/s41467-020-18302-1 (PMC7477573; doi:10.1038/s41467-020-18302-1)
Supplement: Supplementary file 1 — Supplementary Information [file 41467_2020_18302_MOESM1_ESM.pdf]

SUPPLEMENTARY INFORMATION:

Engineered systems of inducible anti-repressors for the next generation of biological programming

Groseclose *et al*.

Supplementary Information Table of Contents

Supplementary Note 1

Supplementary Note 2

Supplementary Note 3

Supplementary Figure 1: Multiple-sequence alignment of LacI and homologues.

Supplementary Figure 2: Workflow for site-saturation of LacI and homologues.

Supplementary Figure 3: Table of mutations for LacI and wild-type and engineered homologues.

Supplementary Figure 4: Genetic architectures of reporters and transcription factor vehicles.

Supplementary Figure 5: Regulatory protein template and plasmid maps.

Supplementary Figure 6: Anti-repression and inverse anti-repression matrices for engineered transcription factors in the lactose repressor (LacI) scaffold.

Supplementary Figure 7: Inverse anti-repression matrices for engineered transcription factors.

Supplementary Figure 8: Performance cards for engineered transcriptional repressors and anti-repressors.

Supplementary Note 4

Supplementary Figure 9: Flow cytometry analysis of biological unit operations (BUO).

Supplementary Figure 10: Transcriptional AND gates.

Supplementary Figure 11: Development of the transcriptional NOR operation.

Supplementary Figure 12: Transcriptional NOR logic gates.

Supplementary Figure 13: Transcriptional NAND and XNOR gates.

Supplementary Figure 14: Phenotypes of engineered and natural transcription factors.

Supplementary Figure 15: Gating and sorting strategy for flow cytometry (analysis and cell sorting).

Supplementary Note 1: We have demonstrated in a previous report that variation in the allosteric end-point (*i.e.*, DNA binding domain) can influence the functional outcome[1, 2]. Given that native LacI, FruR, and RbsR have disparate DNA binding functions, we posited that this could influence our ability to discover positions that confer an allosteric block (*i.e.*, super-repression). Accordingly, the rationale for normalizing the DNA binding domain to YQR is that properties of the lactose repressor native allosteric network are coupled to the native (YQR) DNA binding domain. Thus we hypothesized that properties of putative super-repressor positions in FruR, and RbsR (identified *via* sequence alignment to LacI) require the same allosteric end-point (YQR).

Supplementary Note 2: Super-repression (I^{S}_{YQR}) has been observed in the LacI scaffold at positions 84 [3, 4], 88[4, 5], 95[4, 5] and purportedly at 96[4]. In addition, anti-lacs have been engineered *via* K84A[3], D88A[5], and V95A / V95F[3]. We introduced position 96 as a control, as positions 95 and 96 confer I^{S}_{YQR} phenotypes and 95 facilitates I^{A}_{YQR}. Whereas, position 96 has been reported as I^{S}_{YQR}; however, conferred I^{A}_{YQR} has not been reported. In addition, positions 95-96 can be regarded as a hotspot for super-repression in LacI, given that all three positions support conferred I^{S}_{YQR} as a phenotype.

Supplementary Note 3: Super-repression (I^{S}_{YQR}) at position 88 was previously identified by way of a blue–white screen[5], and in a separate study *via* a similar black-white screen[4]. Using this assay, Suckow *et al*. [4] and Meyer *et al*.[5] measured protein activity of β-galactosidase – which cleaves S-gal and X-gal production (respectively) as a proxy for LacI regulated output, producing a blue or black pigment. For the Suckow *et al*. assay colonies were grown on indicator plates then permeabilized in SDS and chloroform solution. Absorbance of the solution was then measured at 420 nm and phenotypes subsequently assigned. Whereas, Meyer *et al.* measured the optical density (+/- IPTG) directly at the colony forming unit.

In this study and in our recent work[1, 3] we have transitioned to an in solution system in which green fluorescent protein (GFP) is regulated *via* a DNA operator located upstream of the reading frame, which has been broadly adopted by the synthetic biology community and related fields. Results in which this system were used, have been reproduced in both microwell assays and Fluorescence-activated cell sorting (FACS). The differences between the GFP based screen and the blue / black- white screen can be explained as follows: (i) the GFP screen does not involve a coupled reaction as is the case for the blue / black- white screen which is facilitated *via* β-galactosidase; (ii) solid media is used to develop the blue / black- white screens. Accordingly, differences in transport phenomena have to be considered in addition to catalytic limitation. In general the GFP screen in solution is regarded as the more accurate system in terms of evaluating true performance metrics.

Supplementary Figure 1

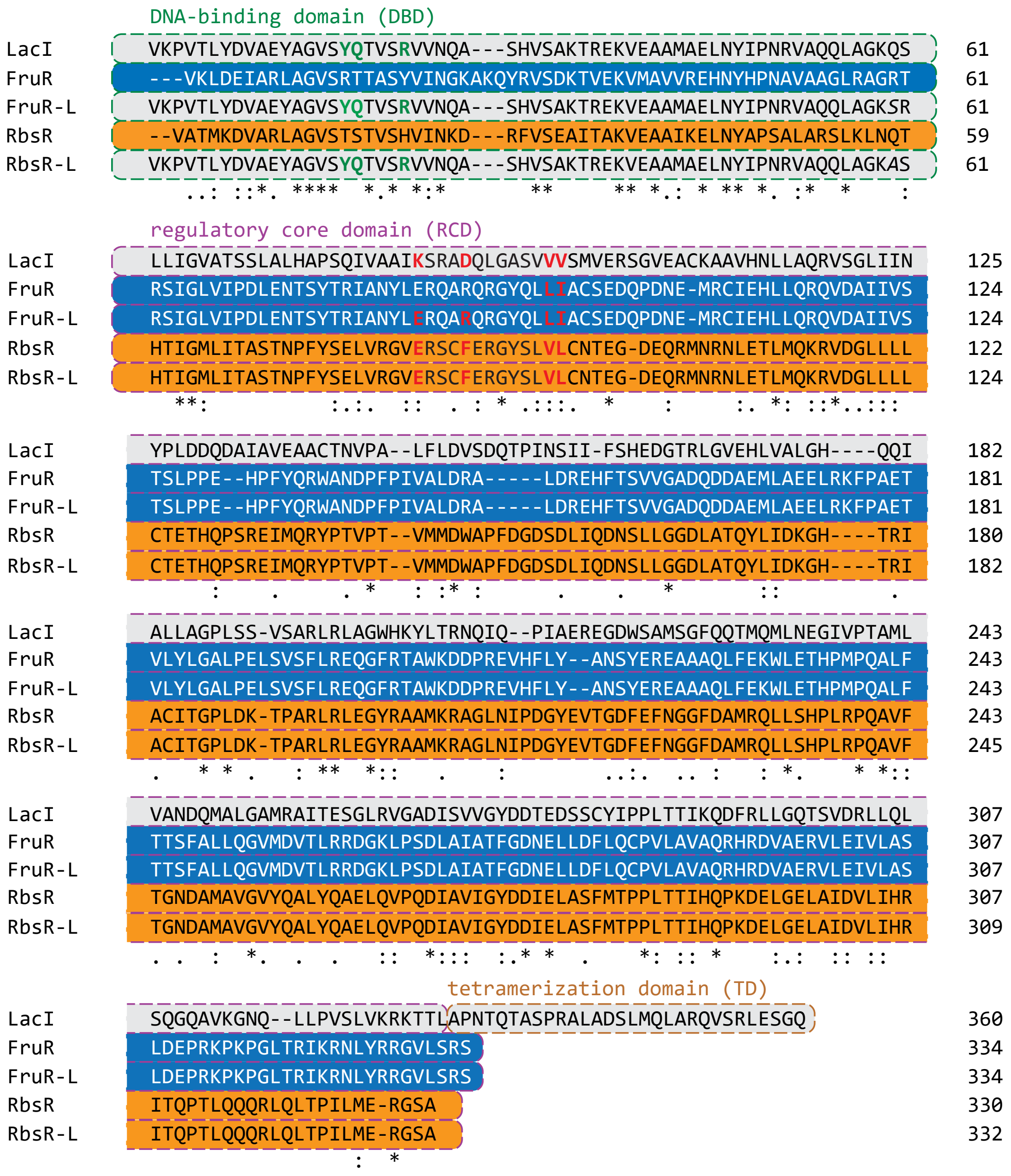

Supplementary Figure 1: Multiple-sequence alignment of LacI and homologues. Protein sequences of wild-type LacI, FruR, RbsR, and chimeric RbsR (RbsR-L) and FruR (RbsR-L) (as classified by Shis, *et al.* [6]). Multiple-sequence alignment performed by EMBL-EBI Clustal Omega (EMBL-EBI). Dashed rectangles of different colors denote different protein domains: DNA-binding domain (DBD) (green), regulatory core domain (RCD) (purple), tetramerization domain (TD, for LacI only) (brown). Green text corresponds to residues Y17, Q18, and R22 in LacI and the chimeric homologues, the residues mutated to confer alternate DNA recognition (ADR). Red text corresponds to residues site-saturated in LacI and homologues to discover super-repressor (X^S) and anti-repressor (X^A) mutants. Numbers at right correspond to residue position number. Symbols at bottom denote primary sequence conservation: (.) denotes weak similarity, (:) denotes high conservation, and (*) denotes sequence identity. Italicized residues at position 60 in FruR-L and RbsR-L denote mutations to the LacI hinge helix in the variants used by Shis *et al.*[6]. Lastly, color shading of boxes denotes origin of a protein domain: grey denotes LacI, orange denotes RbsR, and blue denotes FruR.

Supplementary Figure 2

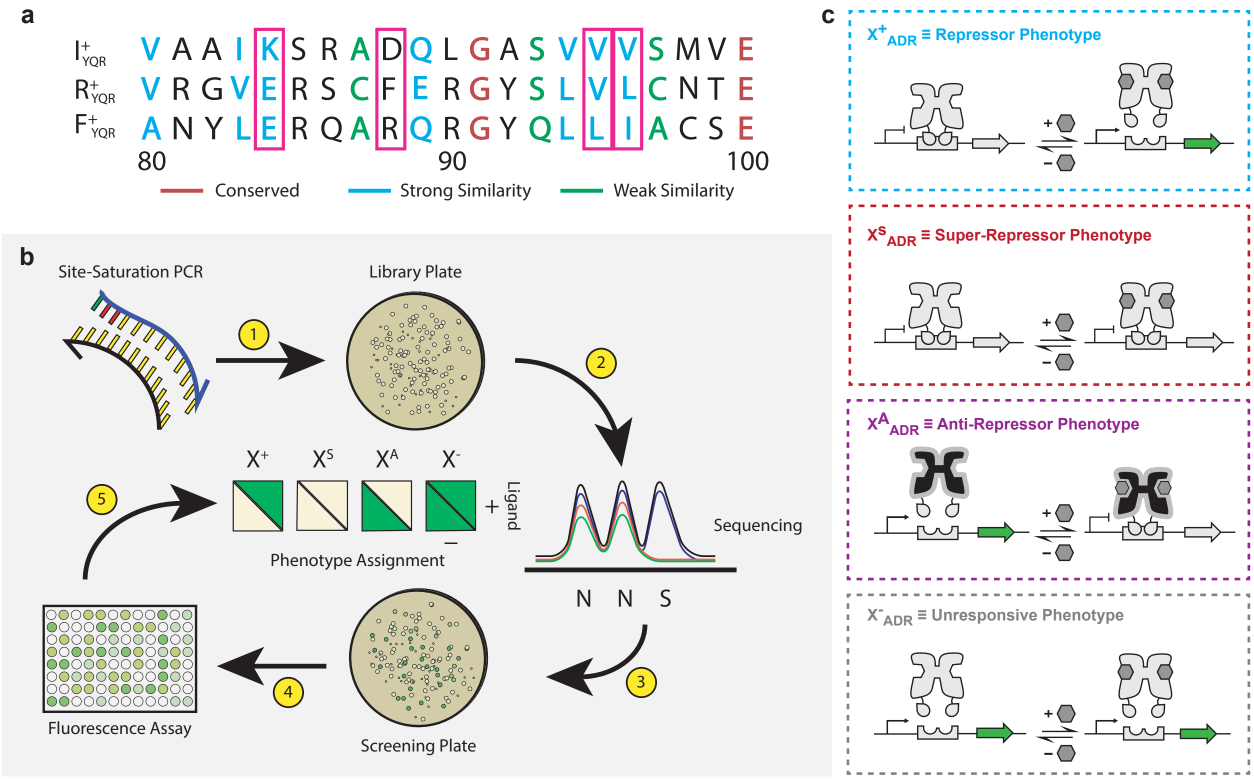

Supplementary Figure 2: Workflow for site-saturation of LacI and homologues. **(a)** Multiple sequence alignment of wild-type LacI and chimeric RbsR and FruR, from position 80 to 100. The primary sequence is listed for each homologue, with black text indicating no similarity, red text indicating conservation, blue text indicating strong similarity, and green text indicating weak similarity. Magenta boxes indicate positions and residues subjected to site-saturation: 84, 88, 95, and 96. **(b)** Workflow for site-saturation and screening for X^S and X^A phenotypes. Informed by the sequence alignment, site-saturation PCR using degenerate (NNS) codons was performed at each position. PCR product was transformed into cells and plated on selection plates. These were streaked, grown, and plasmids were isolated for sequencing. Sequencing confirmed site-saturation at the position in question. The library was then transformed into the reporter cell line (3.32 *E. coli* co-transformed with a *lac* O^1 proximal-driven sfGFP gene). Colonies were picked, grown, and assayed in the absence and presence of the inducer, which allowed phenotype to be assigned. Phenotypes correspond to diagrams shown in **(c)**, phenotypes of transcription factor variants. (i) The repressor, or X^+, phenotype is induced in the presence of the ligand, permitting gene transcription. (ii) The super-repressor, or X^S phenotype, cannot be induced by the ligand, constitutively repressing the gene. This may be due to a disrupted allosteric network. (iii) The anti-repressor, or X^A, phenotype, prevents gene transcription in the presence of the ligand. (iv) The unresponsive, or X^-, phenotype cannot repress the gene, in the presence or absence of the ligand. This may be due to a disruption in interaction between the DNA binding domain (DBD) and the operator, or simply a non-functional protein.

Supplementary Figure 3

	77	79	95	96	105	114	121	154	263	264	275	295	324
F^{+}_{YQR}(WT)	T	I	L	I	N	L	I	E	K	L	D	D	N
F^{S}_{YQR}(L95K)	-	-	K	-	-	-	-	-	-	-	-	-	-
F^{S}_{YQR}(L95I)	-	-	I	-	-	-	-	-	-	-	-	-	-
F^{S}_{YQR}(I96R)	-	-	-	R	-	-	-	-	-	-	-	-	-
F^{S}_{YQR}(I96P)	-	-	-	P	-	-	-	-	-	-	-	-	-
$F^{A(1)}_{YQR}$	S	F	K	-	-	-	-	-	-	-	Q	-	-
$F^{A(2)}_{YQR}$	S	F	I	-	I	F	N	V	N	Q	-	V	I

	84	88	95	96
R^{+}_{YQR}(WT)	E	F	V	L
R^{S}_{YQR}(V95E)	-	-	E	-
$R^{A(1)}_{YQR}$	-	-	D	-
$R^{A(2)}_{YQR}$	-	-	-	P
$R^{A(3)}_{YQR}$	P	-	-	-
$R^{A(4)}_{YQR}$	-	P	-	-

	84	88	95	96
I^{+}_{YQR}(WT)	K	D	V	V
I^{S}_{YQR}(K84A)	A	-	-	-
I^{S}_{YQR}(D88A)	-	A	-	-
I^{S}_{YQR}(V95A)	-	-	A	-
I^{S}_{YQR}(V95F)	-	-	F	-
I^{S}_{YQR}(V96E)	-	-	-	E
I^{S}_{YQR}(V96D)	-	-	-	D
$I^{A(10)}_{YQR}$	P	-	-	-
$I^{A(11)}_{YQR}$	-	-	-	R

Supplementary Figure 3: Table of mutations for LacI and wild-type and engineered homologues. (*top*) Fructose repressor (FruR) chimera (F^{+}_{YQR}), and engineered super-repressors (F^{S}) and anti-repressors (F^{A}), (middle) ribose repressor (RbsR) chimera (R^{+}_{YQR}), and engineered super-repressors (R^{S}) and anti-repressors (R^{A}), (*bottom*) lactose repressor (LacI, I^{+}_{YQR}) and engineered super-repressors (I^{S}) and anti-repressors (I^{A}). Numbers along the top denotes residue position number in protein and along the left-hand side is protein variant. The top variant is wild-type, with the wild-type residue listed. Residues in columns correspond to mutations in that variant, whereas dashes (-) indicate no change from wild-type. Red text emphasizes mutations in variants displaying a super-repressor phenotype (X^{S}) that were used as parents to evolve anti-repressors (X^{A}) using the directed-evolution route. Refer to **Supplementary Note 3** for details on grayed-out text.

Supplementary Figure 4

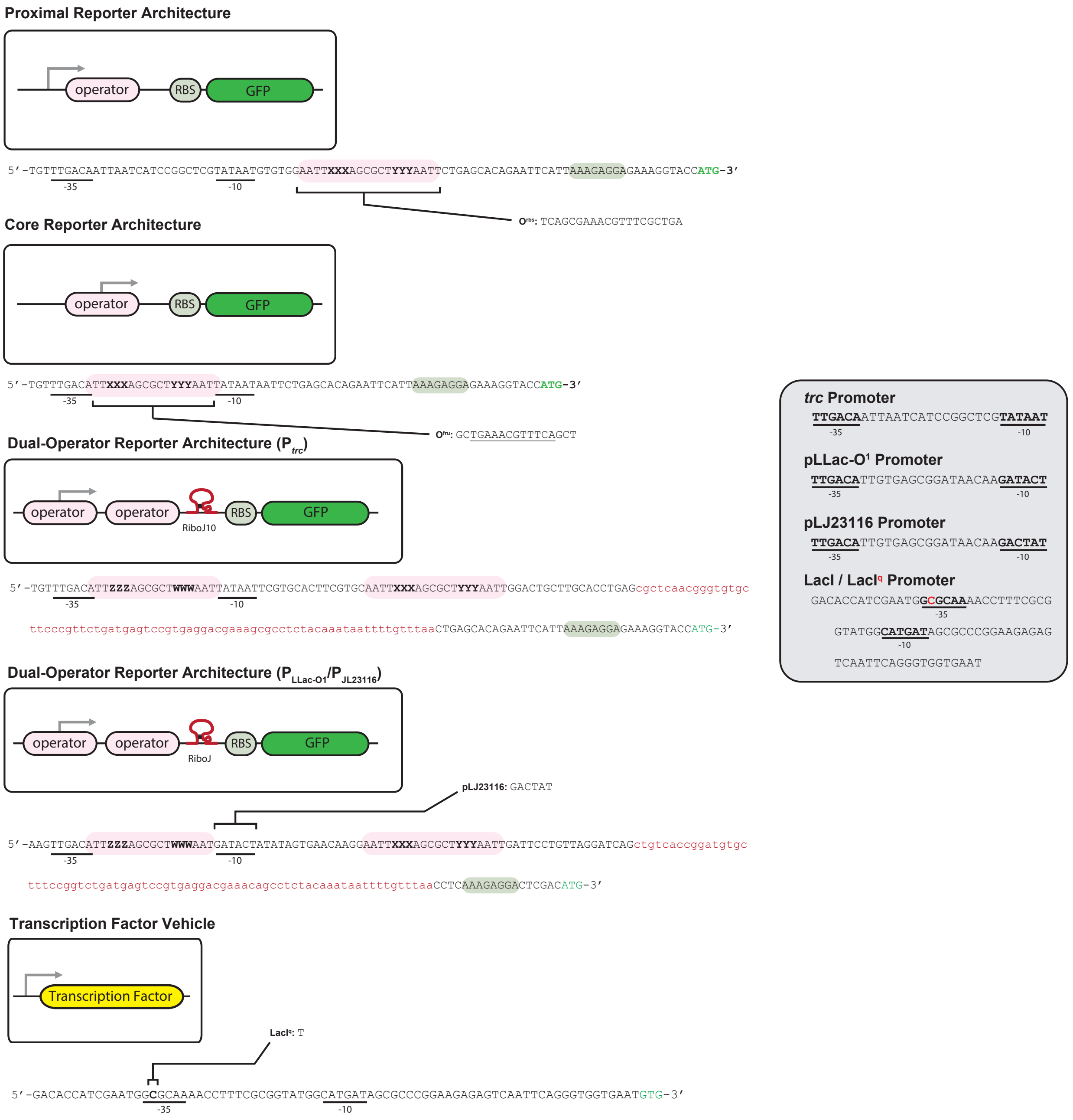

Supplementary Figure 4: Genetic architectures of reporters and transcription factor vehicles. Each architecture displayed includes a gene map depiction, *top* (boxed) and annotated sequences, *bottom*. **(a)** Proximal reporter architecture, **(b)** core reporter architecture, **(c)** dual-operator reporter architecture (*trc* promoter variant), **(d)** dual-operator reporter architecture (LLac-O1/JL23116 promoter variant), and **(e)** transcription factor vehicle. In gene maps and sequences, promoters are depicted by gray arrows, operators are shown as pink ovals, ribosome binding sites (RBS) are shown as light green ovals, reporter genes (GFP) are shown as dark green ovals, genetic insulators as red hairpins and text, and transcription factors as yellow ovals. Green text indicates start codons of reporter genes and locations of promoter hexamers are underlined. Callouts correspond to sequence variations for certain architectures. The underlined portion of O^{fru} is the region cited as the *fruBKA* operator, identified by Ramseier *et al.* [7]; the non-underlined portions are nucleotides added to ensure correct hexamer spacing. Gray box to right displays annotated wild-type sequences of promoters used.

Supplementary Figure 5

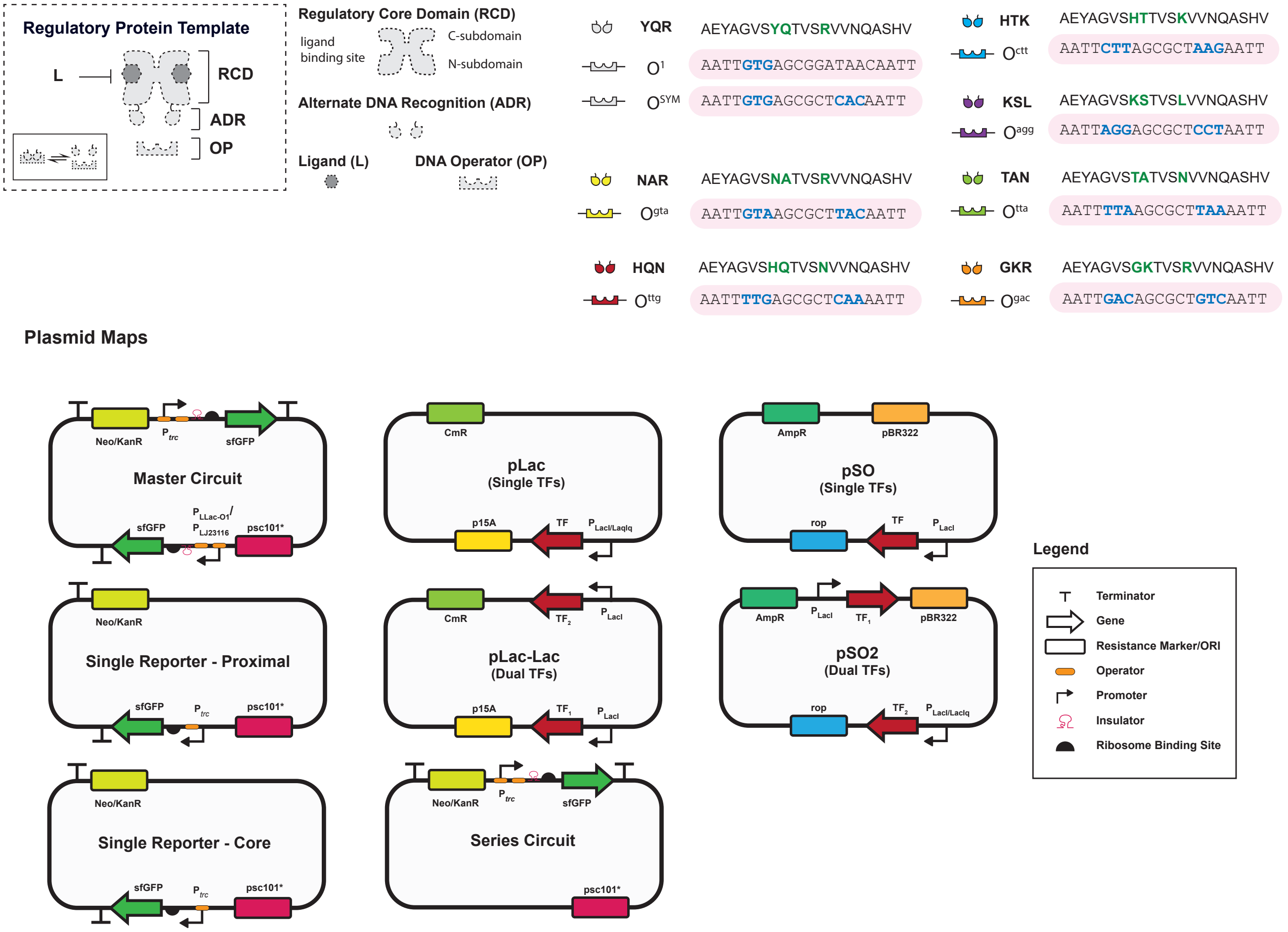

Supplementary Figure 5: Regulatory protein template and plasmid maps. *Top (left),* The regulatory protein template. A regulatory protein (commonly, transcription factors, TFs) consist of several distinct regions, as depicted by the cartoon. The regulatory core domain (RCD), which can be sub-divided into the N- and C-subdomains, between which a cleft is located containing the ligand binding site, allows response to the ligand (L). The RCD is responsible for transmitting the allosteric signal. The DNA-binding domain is responsible for interacting with operator DNA (OP), preventing transcription in the DNA-bound state. Here, it is shown as the alternate DNA recognition (ADR) module. Ligand binding modulates the equilibrium of TF-DNA interactions, allowing transcriptional regulation. *Top (right)*, cognate pairs of ADR modules and operators. Identical colors denote cognate (or designed) interactions between ADR modules and operator DNA sequences. ADR are labeled by a three-letter notation corresponding to residues at positions 17, 18, and 22 (respective to the LacI wild-type DBD), shown in green text. Operators are generally labeled by a three-letter notation corresponding to their nucleotides (and their palindrome), shown in blue text. O^1 is the wild-type *lac* operon O^1 operator, while O^{SYM} is the symmetric (palindromic) version of the O^1 operator. *Bottom,* maps of plasmid architectures used. Starting *top left*, descending columns: “Master circuit,” adapted from Rondon *et al.*[1]; single-copy reporter plasmid, with operator in the proximal position; single-copy reporter plasmid, with operator in the core position; pLac; pLac-Lac (bearing two TFs); series reporter architecture; pSO; and pSO2 (bearing two TFs). Text in plasmid maps emphasizes variation in features.

Supplementary Figure 6

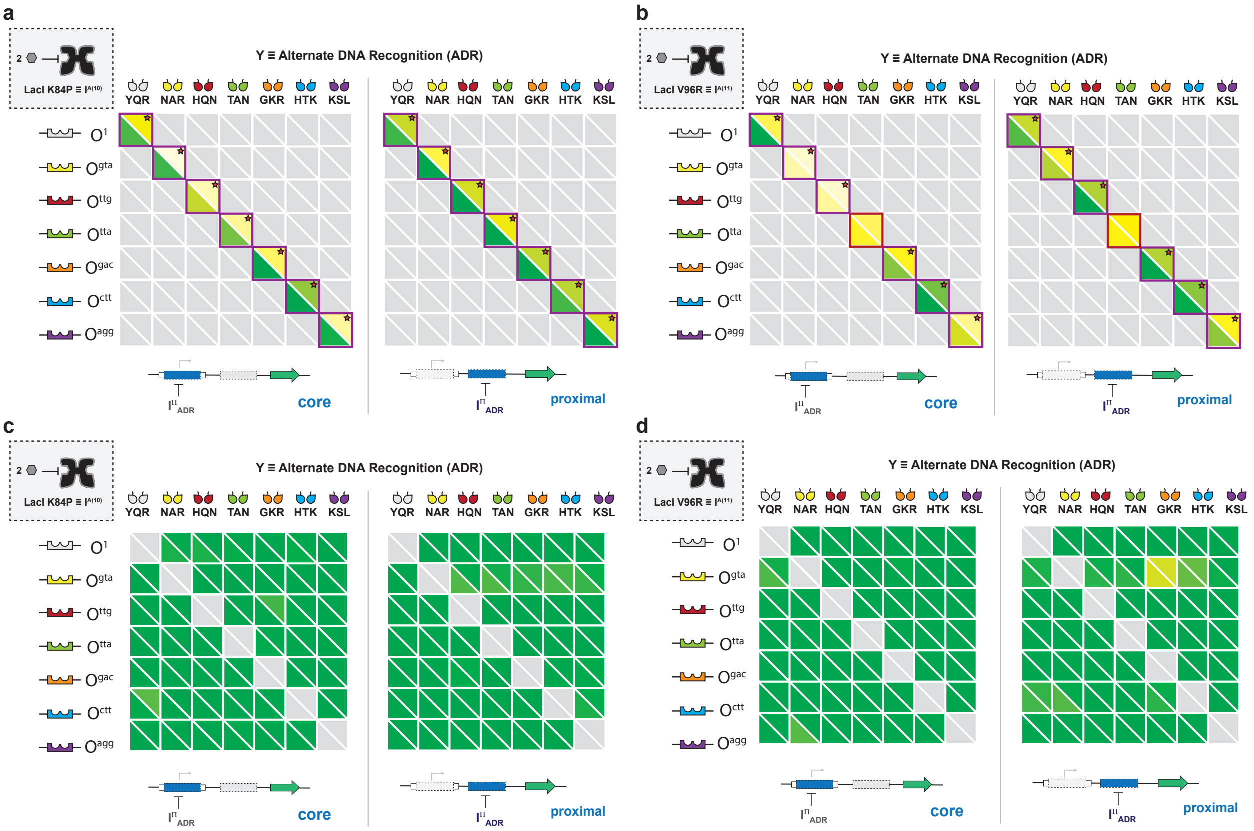

Supplementary Figure 6: Anti-repression and inverse anti-repression matrices for engineered transcription factors in the lactose repressor (LacI) scaffold. **(a)** Anti-repression matrix for anti-LacI$^{(10)}$; **(b)** anti-repression matrix for anti-LacI$^{(11)}$; **(c)** inverse anti-repression matrix for anti-LacI$^{(10)}$; **(d)** inverse anti-repression matrix for anti-LacI$^{(11)}$. Numbering continues from schema outlined by Rondon and Wilson[2]. Each square represents an operator-DNA-binding domain pairing. DBDs are across the top of each matrix, with corresponding operators along the left side. The bottom-left sector of each square shows the mean fluorescence intensity (OD_{600} normalized, ex: 485 nm, em: 510 nm) in the absence of the inducer, whereas the top-right sector shows the normalized fluorescence intensity in the presence of the inducer. Shaded colors of sectors correspond to the GFP output.. All values are standardized to the observed maximum output (measured with a $LacI_{null}$ control plasmid) for a given operator and operator position (core or proximal) and values correspond to the mean of n = 6 biological replicates. The left matrix in each set corresponds to testing with the operator in the core position and the right matrix corresponds to testing with the operator in the proximal position. In **(a)** and **(b)**, anti-repression matrices show pairings not exhibiting the unresponsive (X^-) phenotype (grayed out). Red stars denote pairings that exhibited a statistically-significant (α = 0.001) difference in fluorescence intensity value between the two states using a Student's two-tailed t-test. Purple outlined boxes signify an anti-repressor (X^A) phenotype, whereas red outlined boxes signify a super-repressor (X^S) phenotype. In **(c)** and **(d)**, inverse anti-repression matrices, pairings did not show a statistically-significant difference between states and expression was over 50% of maximum, indicating an unresponsive (X^- phenotype).

Supplementary Figure 7

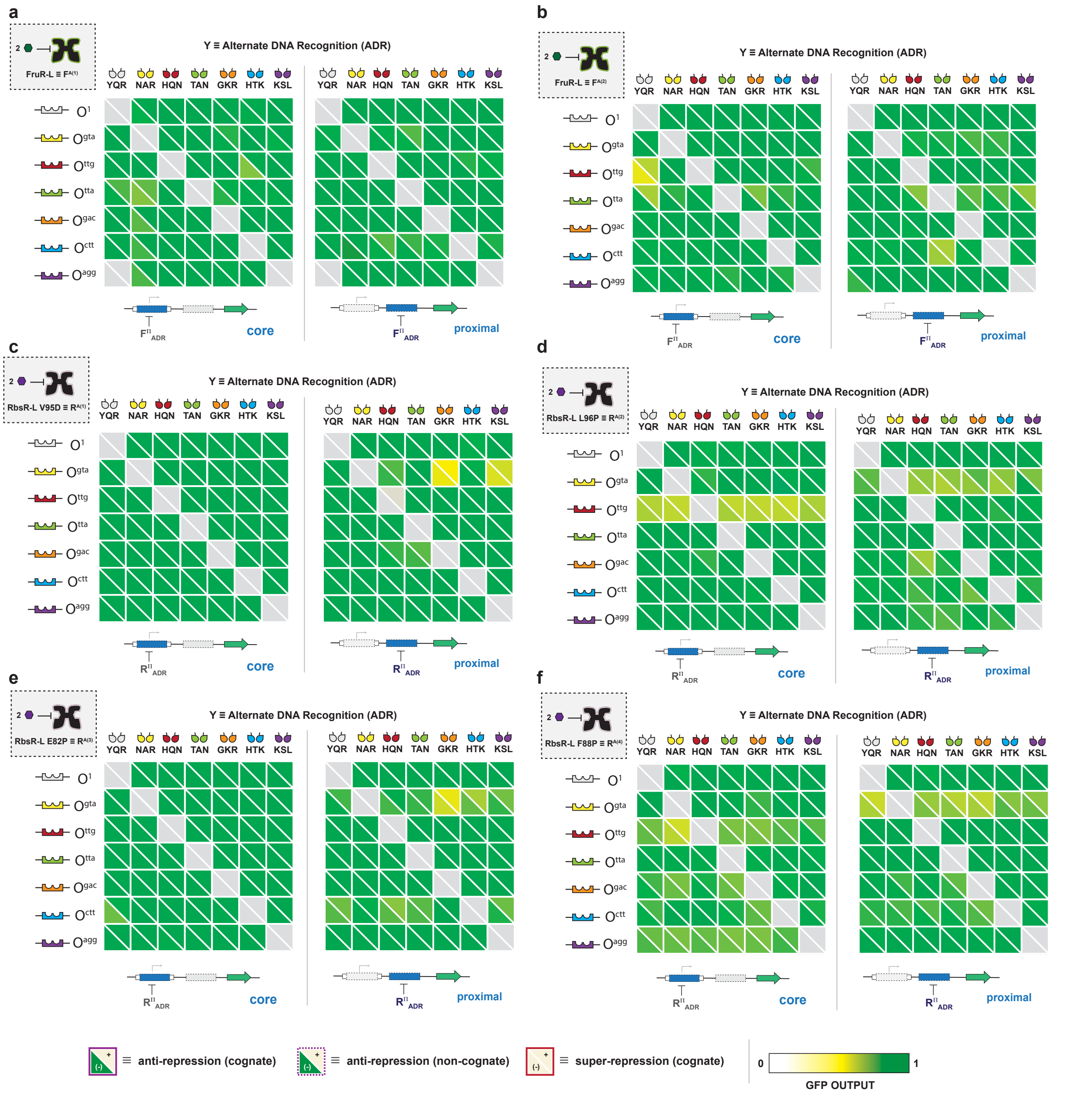

Supplementary Figure 7: Inverse anti-repression matrices for engineered transcription factors. **(a)** anti-FruR$^{(1)}$; **(b)** anti-FruR$^{(2)}$; **(c)** anti-RbsR$^{(1)}$; **(d)** anti-RbsR$^{(2)}$; **(e)** anti-RbsR$^{(3)}$; and **(f)** anti-RbsR$^{(4)}$. Each square represents an operator-DNA-binding domain pairing. DBDs are across the top of each matrix, with corresponding operators along the left side. The bottom-left sector of each square shows the mean fluorescence intensity (OD_{600} normalized, ex: 485 nm, em: 510 nm) in the absence of the inducer, whereas the top-right sector shows the normalized fluorescence intensity in the presence of the inducer. Shaded colors of sectors correspond to the relative GFP output, scalebar shown at bottom. All values are standardized to the observed maximum output (measured with a $LacI_{null}$ control plasmid) for a given operator and operator position (core or proximal). All pairings shown did not exhibit a statistically-significant (α = 0.001) difference in fluorescence intensity value between the two states using a Student's two-tailed t-test and are classified as X^- phenotypes. See **Fig. 4** for anti-repression matrices. Values correspond to the mean of n = 6 biological replicates. The left matrix in each set corresponds to testing with the operator in the core position and the right matrix corresponds to testing with the operator in the proximal position.

Supplementary Figure 8

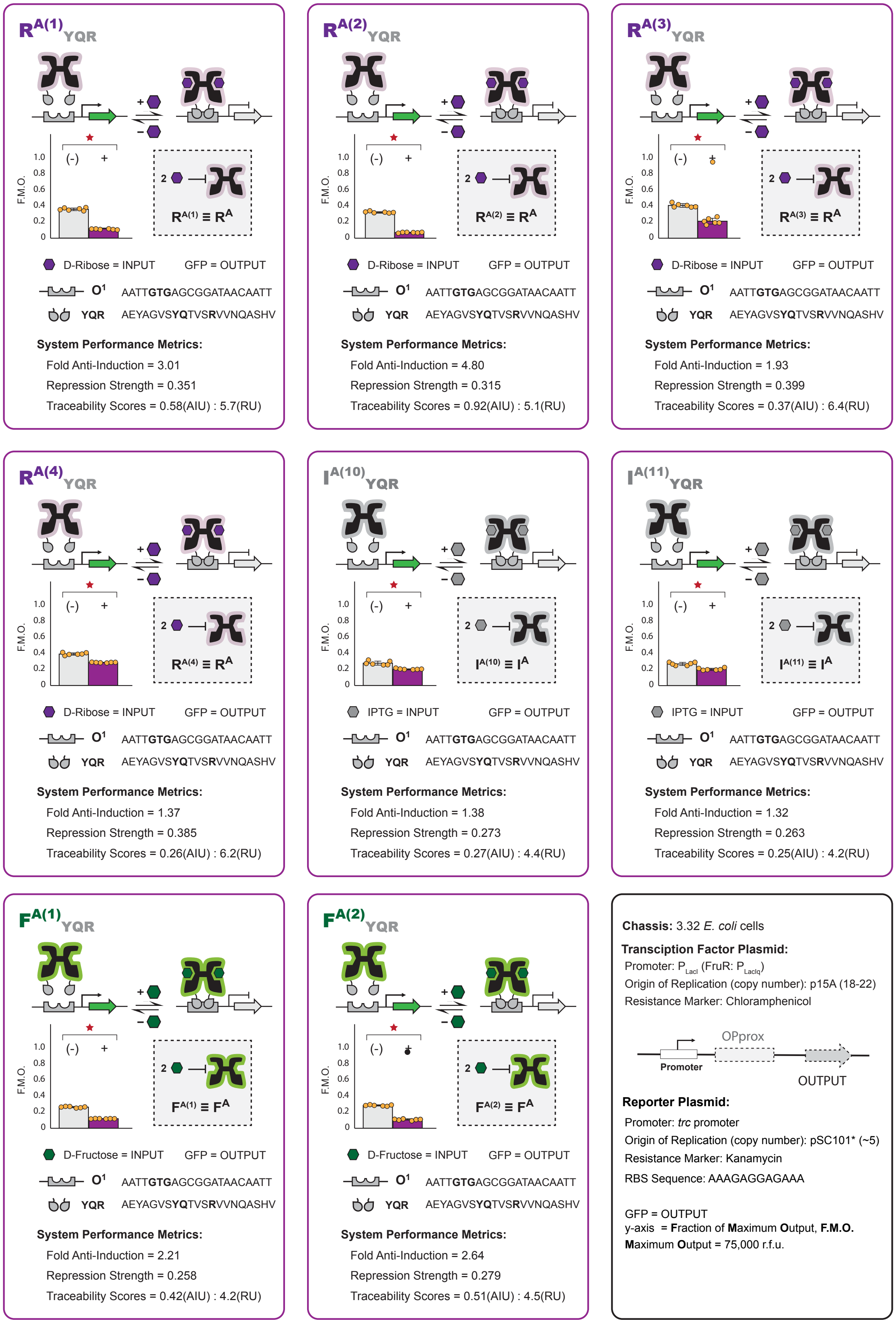

AIU = anti-induction units, relative to I^{+}_{YQR} | O^{1} unit operation

RU = repression units, relative to I^{+}_{YQR} | O^{1} unit operation

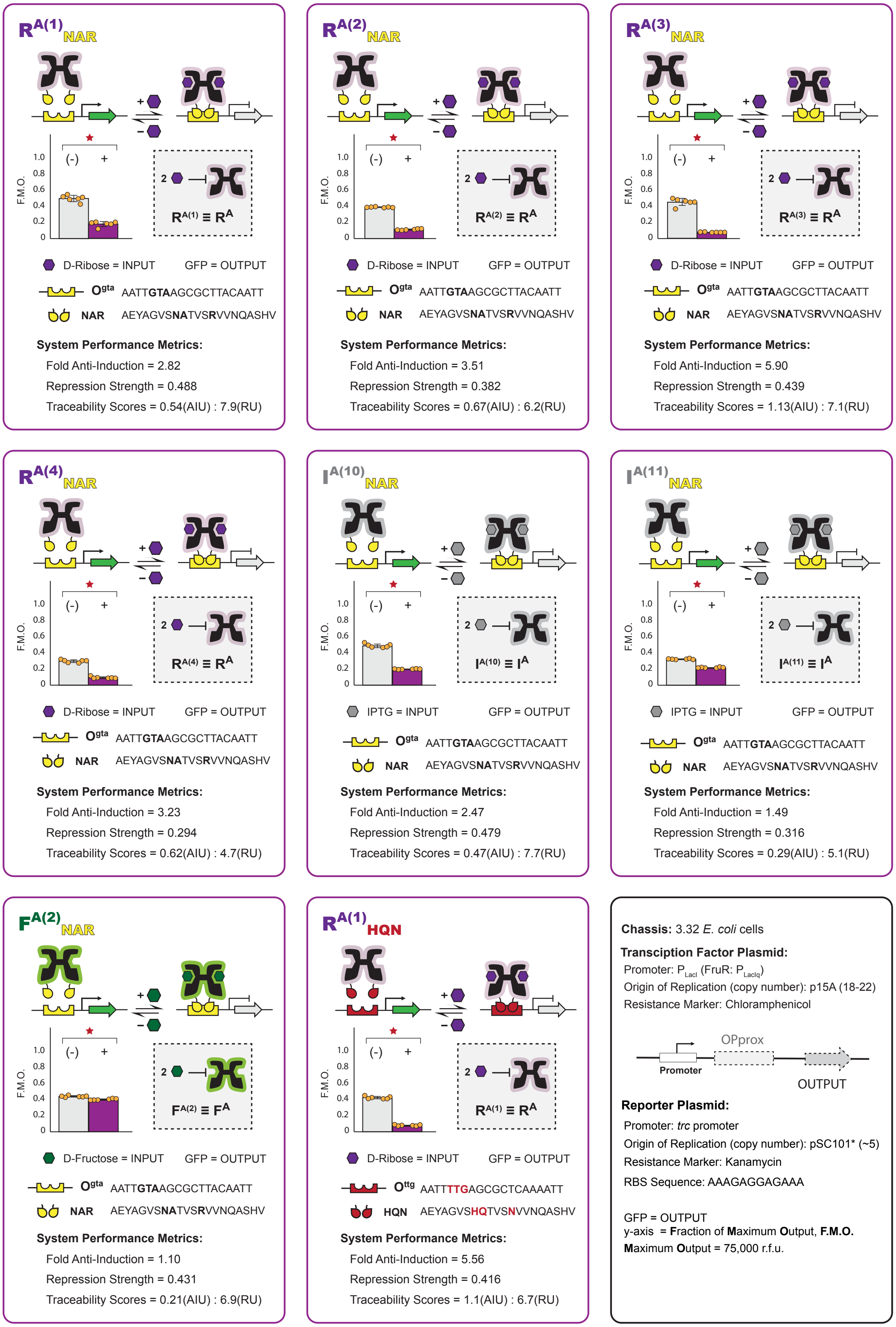

AIU = anti-induction units, relative to I^{+}_{YQR} | O^1 unit operation

RU = repression units, relative to I^{+}_{YQR} | O^1 unit operation

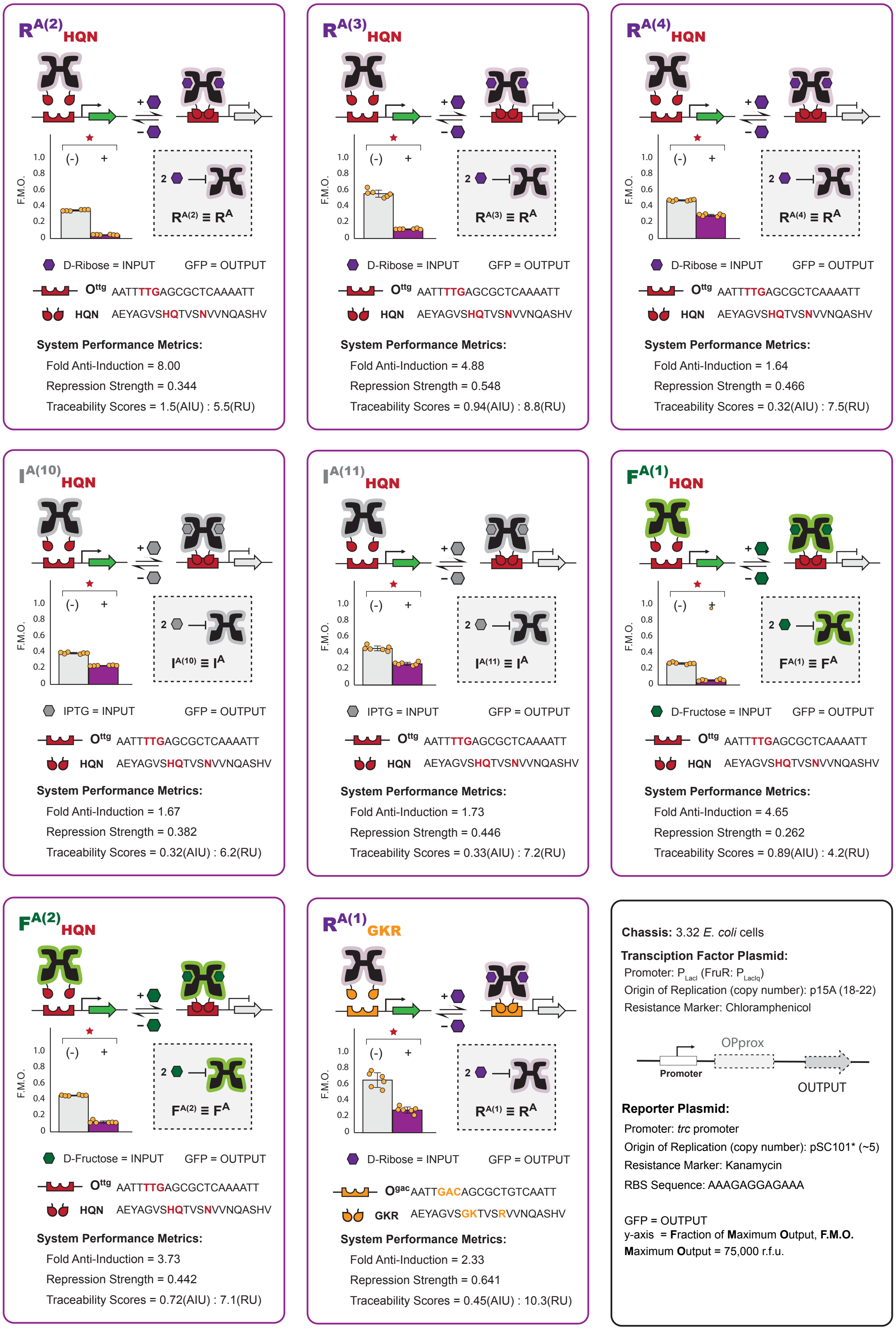

AIU = anti-induction units, relative to I^{+}_{YQR} | O^{1} unit operation

RU = repression units, relative to I^{+}_{YQR} | O^{1} unit operation

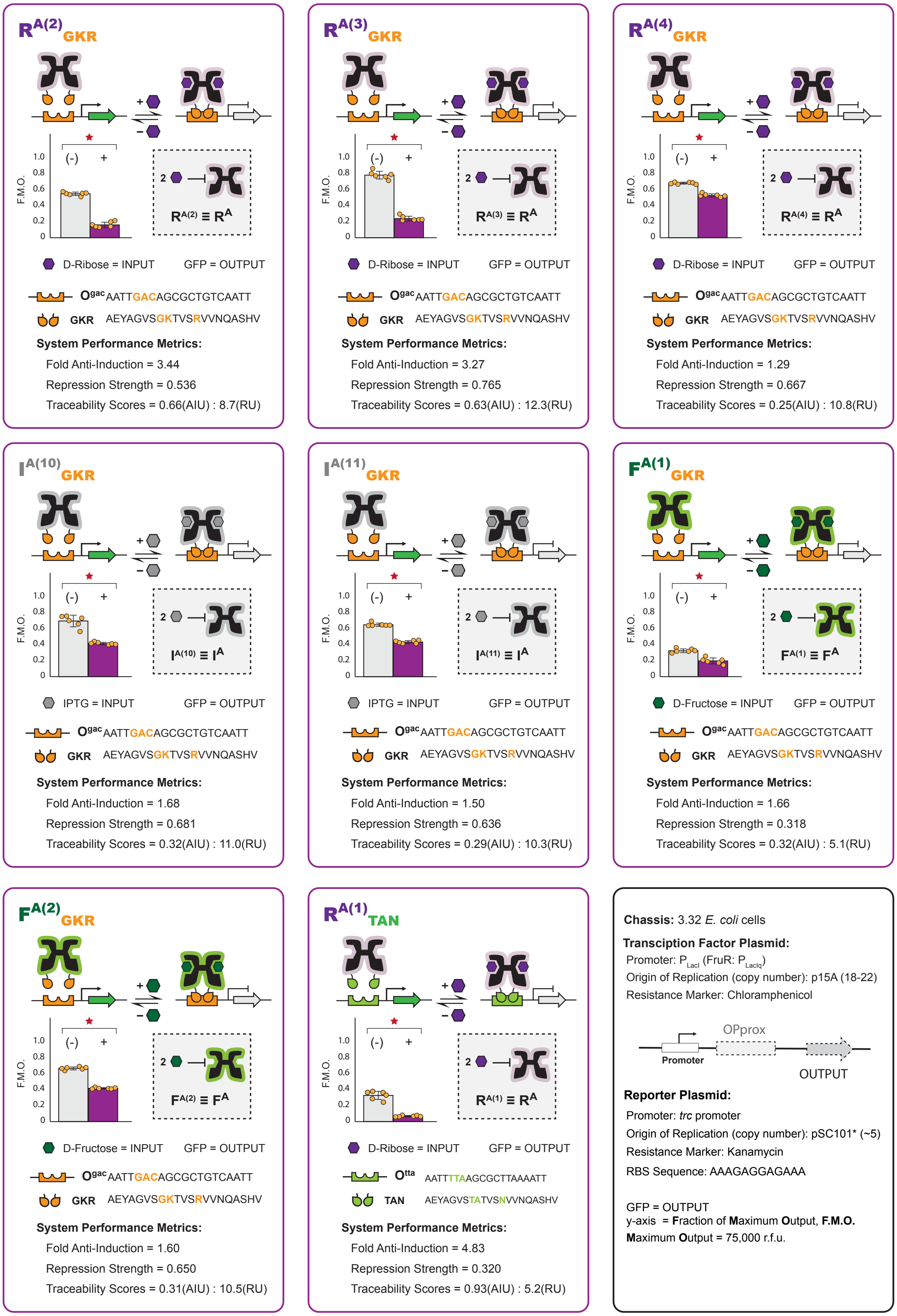

AIU = anti-induction units, relative to I^{+}_{YQR} | O^{1} unit operation

RU = repression units, relative to I^{+}_{YQR} | O^{1} unit operation

$R^{A(2)}$ TAN

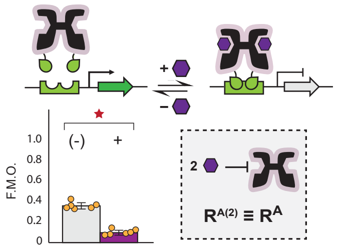

D-Ribose = INPUT GFP = OUTPUT

O^{tta} AATTTTAAGCGCTTAAAATT

TAN AEYAGVSTATVSNVVNQASHV

System Performance Metrics:

Fold Anti-Induction = 3.73

Repression Strength = 0.351

Traceability Scores = 0.72(AIU) : 5.7(RU)

$R^{A(3)}$ TAN

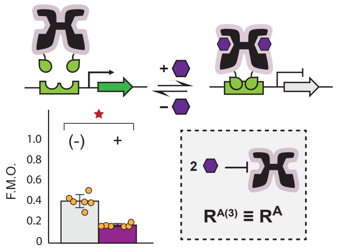

D-Ribose = INPUT GFP = OUTPUT

O^{tta} AATTTTAAGCGCTTAAAATT

TAN AEYAGVSTATVSNVVNQASHV

System Performance Metrics:

Fold Anti-Induction = 2.34

Repression Strength = 0.400

Traceability Scores = 0.45(AIU) : 6.5(RU)

$R^{A(4)}$ TAN

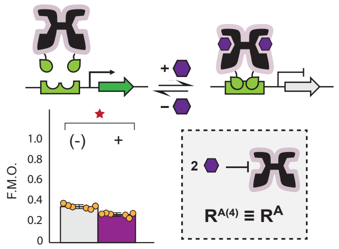

D-Ribose = INPUT GFP = OUTPUT

O^{tta} AATTTTAAGCGCTTAAAATT

TAN AEYAGVSTATVSNVVNQASHV

System Performance Metrics:

Fold Anti-Induction = 1.28

Repression Strength = 0.343

Traceability Scores = 0.25(AIU) : 5.5(RU)

$I^{A(10)}$ TAN

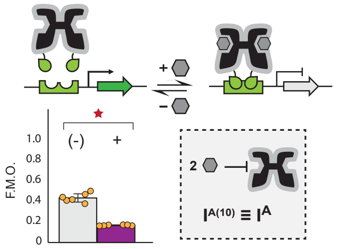

IPTG = INPUT GFP = OUTPUT

O^{tta} AATTTTAAGCGCTTAAAATT

TAN AEYAGVSTATVSNVVNQASHV

System Performance Metrics:

Fold Anti-Induction = 2.57

Repression Strength = 0.425

Traceability Scores = 0.50(AIU) : 6.8(RU)

$F^{A(1)}$ TAN

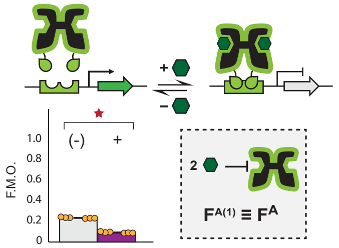

D-Fructose = INPUT GFP = OUTPUT

O^{tta} AATTTTAAGCGCTTAAAATT

TAN AEYAGVSTATVSNVVNQASHV

System Performance Metrics:

Fold Anti-Induction = 2.52

Repression Strength = 0.229

Traceability Scores = 0.48(AIU) : 3.7(RU)

$F^{A(2)}$ TAN

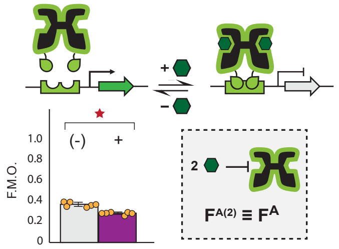

D-Fructose = INPUT GFP = OUTPUT

O^{tta} AATTTTAAGCGCTTAAAATT

TAN AEYAGVSTATVSNVVNQASHV

System Performance Metrics:

Fold Anti-Induction = 1.31

Repression Strength = 0.364

Traceability Scores = 0.25(AIU) : 5.9(RU)

$R^{A(1)}$ HTK

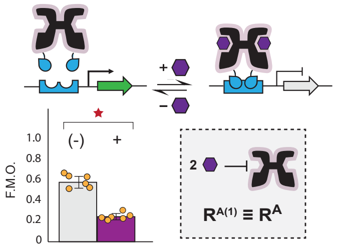

D-Ribose = INPUT GFP = OUTPUT

O^{ctt} AATTCTTAGCGCTAAGAATT

HTK AEYAGVSHTTVSKVVNQASHV

System Performance Metrics:

Fold Anti-Induction = 2.36

Repression Strength = 0.568

Traceability Scores = 0.45(AIU) : 9.2(RU)

$R^{A(2)}$ HTK

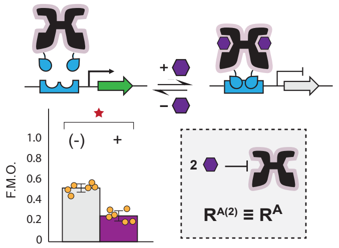

D-Ribose = INPUT GFP = OUTPUT

O^{ctt} AATTCTTAGCGCTAAGAATT

HTK AEYAGVSHTTVSKVVNQASHV

System Performance Metrics:

Fold Anti-Induction = 2.08

Repression Strength = 0.514

Traceability Scores = 0.40(AIU) : 8.3(RU)

Chassis: 3.32 *E. coli* cells

Transciption Factor Plasmid:

Promoter: P_{LacI} (FruR: P_{LacIq})

Origin of Replication (copy number): p15A (18-22)

Resistance Marker: Chloramphenicol

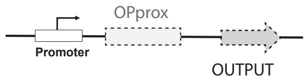

Reporter Plasmid:

Promoter: *trc* promoter

Origin of Replication (copy number): pSC101* (~5)

Resistance Marker: Kanamycin

RBS Sequence: AAAGAGGAGAAA

GFP = OUTPUT

y-axis = **F**raction of **M**aximum **O**utput, **F.M.O.**

Maximum **O**utput = 75,000 r.f.u.

AIU = anti-induction units, relative to I^{+}_{YQR} | O^1 unit operation

RU = repression units, relative to I^{+}_{YQR} | O^1 unit operation

$R^{A(3)}_{HTK}$

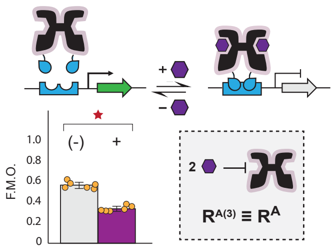

D-Ribose = INPUT GFP = OUTPUT

O^{ctt} AATTCTTAGCGCTAAGAATT

HTK AEYAGVSHTTVSKVVNQASHV

System Performance Metrics:

Fold Anti-Induction = 1.68

Repression Strength = 0.556

Traceability Scores = 0.32(AIU) : 9.0(RU)

$R^{A(4)}_{HTK}$

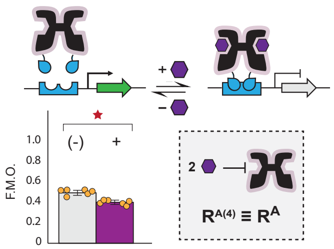

D-Ribose = INPUT GFP = OUTPUT

O^{ctt} AATTCTTAGCGCTAAGAATT

HTK AEYAGVSHTTVSKVVNQASHV

System Performance Metrics:

Fold Anti-Induction = 1.23

Repression Strength = 0.482

Traceability Scores = 0.24(AIU) : 7.8(RU)

$I^{A(10)}_{HTK}$

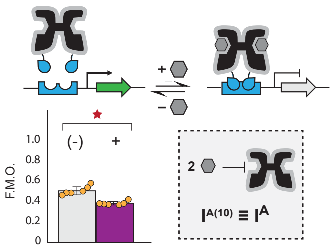

IPTG = INPUT GFP = OUTPUT

O^{ctt} AATTCTTAGCGCTAAGAATT

HTK AEYAGVSHTTVSKVVNQASHV

System Performance Metrics:

Fold Anti-Induction = 1.31

Repression Strength = 0.495

Traceability Scores = 0.25(AIU) : 8.0(RU)

$I^{A(11)}_{HTK}$

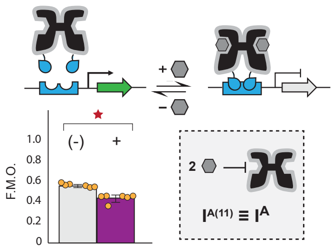

IPTG = INPUT GFP = OUTPUT

O^{ctt} AATTCTTAGCGCTAAGAATT

HTK AEYAGVSHTTVSKVVNQASHV

System Performance Metrics:

Fold Anti-Induction = 1.26

Repression Strength = 0.545

Traceability Scores = 0.24(AIU) : 8.8(RU)

$F^{A(2)}_{HTK}$

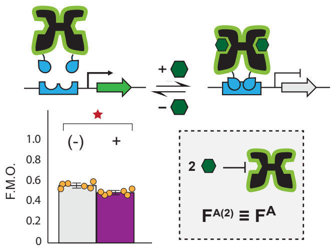

D-Fructose = INPUT GFP = OUTPUT

O^{ctt} AATTCTTAGCGCTAAGAATT

HTK AEYAGVSHTTVSKVVNQASHV

System Performance Metrics:

Fold Anti-Induction = 1.14

Repression Strength = 0.549

Traceability Scores = 0.22(AIU) : 8.9(RU)

$R^{A(1)}_{KSL}$

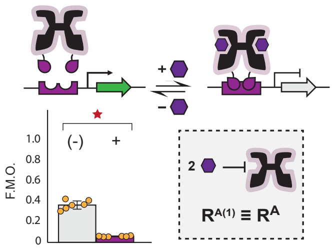

D-Ribose = INPUT GFP = OUTPUT

O^{agg} AATTAGGAGCGCTCCTAATT

KSL AEYAGVSKSTVSLVVNQASHV

System Performance Metrics:

Fold Anti-Induction = 6.19

Repression Strength = 0.358

Traceability Scores = 1.19(AIU) : 5.8(RU)

$R^{A(2)}_{KSL}$

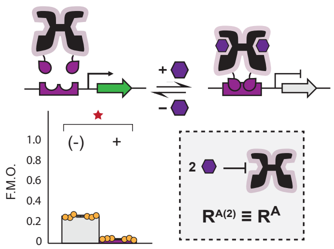

D-Ribose = INPUT GFP = OUTPUT

O^{agg} AATTAGGAGCGCTCCTAATT

KSL AEYAGVSKSTVSLVVNQASHV

System Performance Metrics:

Fold Anti-Induction = 6.48

Repression Strength = 0.260

Traceability Scores = 1.25(AIU) : 4.2(RU)

$R^{A(3)}_{KSL}$

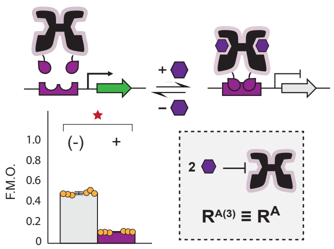

D-Ribose = INPUT GFP = OUTPUT

O^{agg} AATTAGGAGCGCTCCTAATT

KSL AEYAGVSKSTVSLVVNQASHV

System Performance Metrics:

Fold Anti-Induction = 4.42

Repression Strength = 0.481

Traceability Scores = 0.85(AIU) : 7.8(RU)

Chassis: 3.32 *E. coli* cells

Transciption Factor Plasmid:

Promoter: P_{LacI} (FruR: P_{LacIq})

Origin of Replication (copy number): p15A (18-22)

Resistance Marker: Chloramphenicol

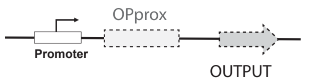

Reporter Plasmid:

Promoter: *trc* promoter

Origin of Replication (copy number): pSC101* (~5)

Resistance Marker: Kanamycin

RBS Sequence: AAAGAGGAGAAA

GFP = OUTPUT

y-axis = **F**raction of **M**aximum **O**utput, **F.M.O.**

Maximum **O**utput = 75,000 r.f.u.

AIU = anti-induction units, relative to I^{+}_{YQR} | O^{1} unit operation

RU = repression units, relative to I^{+}_{YQR} | O^{1} unit operation

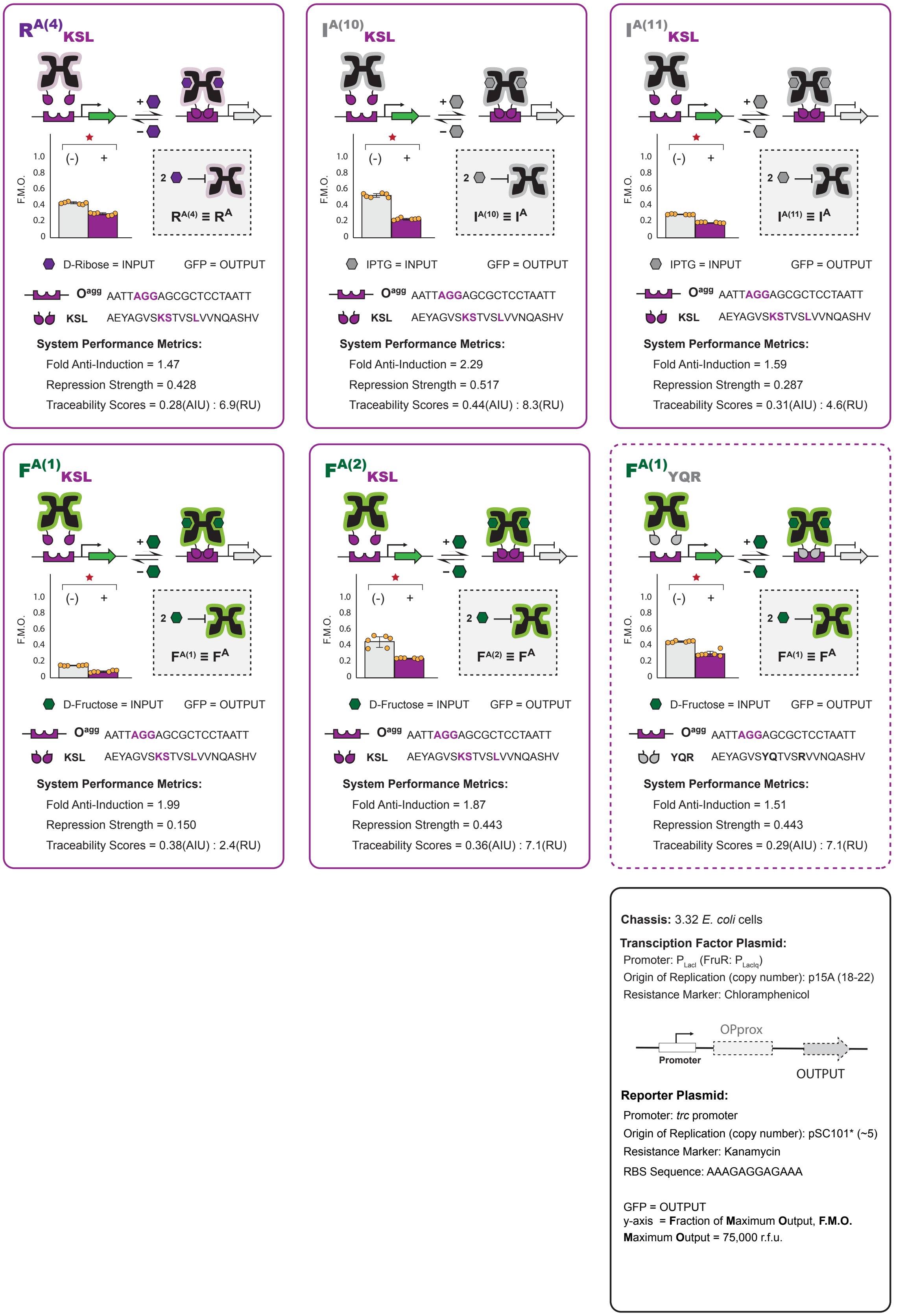

AIU = anti-induction units, relative to I^{+}_{YQR} | O^{1} unit operation

RU = repression units, relative to I^{+}_{YQR} | O^{1} unit operation

$R^{A(1)}_{YQR}$

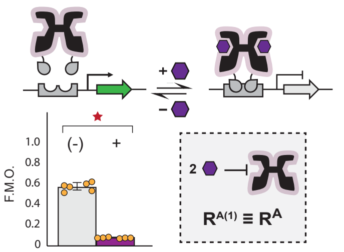

D-Ribose = INPUT GFP = OUTPUT

O^1 ATT**GTG**AGCGGATAACAATT

YQR AEYAGVS**YQ**TVS**R**VVNQASHV

System Performance Metrics:

Fold Anti-Induction = 7.10

Repression Strength = 0.556

Traceability Scores = 1.37(AIU) : 9.0(RU)

$R^{A(2)}_{YQR}$

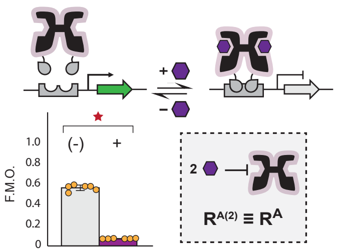

D-Ribose = INPUT GFP = OUTPUT

O^1 ATT**GTG**AGCGGATAACAATT

YQR AEYAGVS**YQ**TVS**R**VVNQASHV

System Performance Metrics:

Fold Anti-Induction = 8.23

Repression Strength = 0.552

Traceability Scores = 1.58(AIU) : 8.9(RU)

$R^{A(3)}_{YQR}$

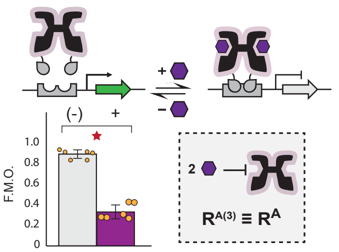

D-Ribose = INPUT GFP = OUTPUT

O^1 ATT**GTG**AGCGGATAACAATT

YQR AEYAGVS**YQ**TVS**R**VVNQASHV

System Performance Metrics:

Fold Anti-Induction = 2.71

Repression Strength = 0.872

Traceability Scores = 0.52(AIU) : 14.1(RU)

$R^{A(4)}_{YQR}$

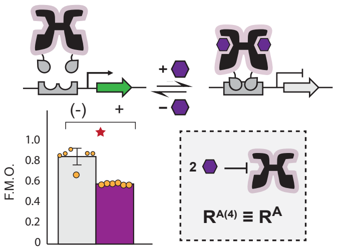

D-Ribose = INPUT GFP = OUTPUT

O^1 ATT**GTG**AGCGGATAACAATT

YQR AEYAGVS**YQ**TVS**R**VVNQASHV

System Performance Metrics:

Fold Anti-Induction = 1.46

Repression Strength = 0.832

Traceability Scores = 0.28(AIU) : 13.4(RU)

$I^{A(10)}_{YQR}$

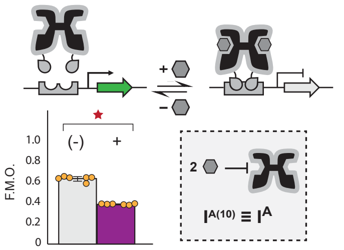

IPTG = INPUT GFP = OUTPUT

O^1 ATT**GTG**AGCGGATAACAATT

YQR AEYAGVS**YQ**TVS**R**VVNQASHV

System Performance Metrics:

Fold Anti-Induction = 1.65

Repression Strength = 0.622

Traceability Scores = 0.32(AIU) : 10.0(RU)

$I^{A(11)}_{YQR}$

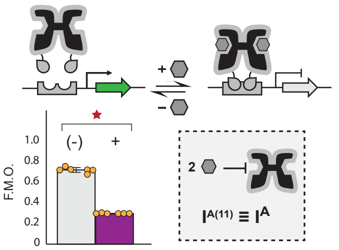

IPTG = INPUT GFP = OUTPUT

O^1 ATT**GTG**AGCGGATAACAATT

YQR AEYAGVS**YQ**TVS**R**VVNQASHV

System Performance Metrics:

Fold Anti-Induction = 2.43

Repression Strength = 0.706

Traceability Scores = 0.47(AIU) : 11.4(RU)

$F^{A(1)}_{YQR}$

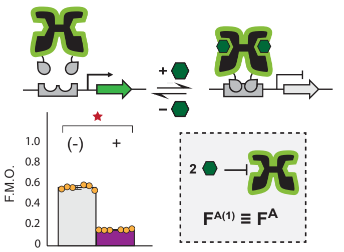

D-Fructose = INPUT GFP = OUTPUT

O^1 ATT**GTG**AGCGGATAACAATT

YQR AEYAGVS**YQ**TVS**R**VVNQASHV

System Performance Metrics:

Fold Anti-Induction = 3.66

Repression Strength = 0.554

Traceability Scores = 0.70(AIU) : 8.9(RU)

$F^{A(2)}_{YQR}$

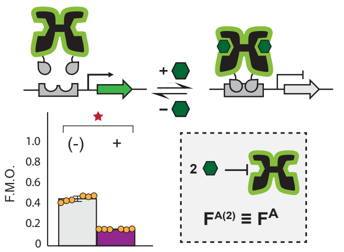

D-Fructose = INPUT GFP = OUTPUT

O^1 ATT**GTG**AGCGGATAACAATT

YQR AEYAGVS**YQ**TVS**R**VVNQASHV

System Performance Metrics:

Fold Anti-Induction = 2.85

Repression Strength = 0.447

Traceability Scores = 0.55(AIU) : 7.2(RU)

Chassis: 3.32 *E. coli* cells

Transciption Factor Plasmid:

Promoter: P_{LacI} (FruR: P_{LacIq})

Origin of Replication (copy number): p15A (18-22)

Resistance Marker: Chloramphenicol

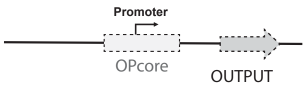

Reporter Plasmid:

Promoter: *trc* promoter

Origin of Replication (copy number): pSC101* (~5)

Resistance Marker: Kanamycin

RBS Sequence: AAAGAGGAGAAA

GFP = OUTPUT

y-axis = **F**raction of **M**aximum **O**utput, **F.M.O.**

Maximum **O**utput = 75,000 r.f.u.

AIU = anti-induction units, relative to I^{+}_{YQR} | O^1 unit operation

RU = repression units, relative to I^{+}_{YQR} | O^1 unit operation

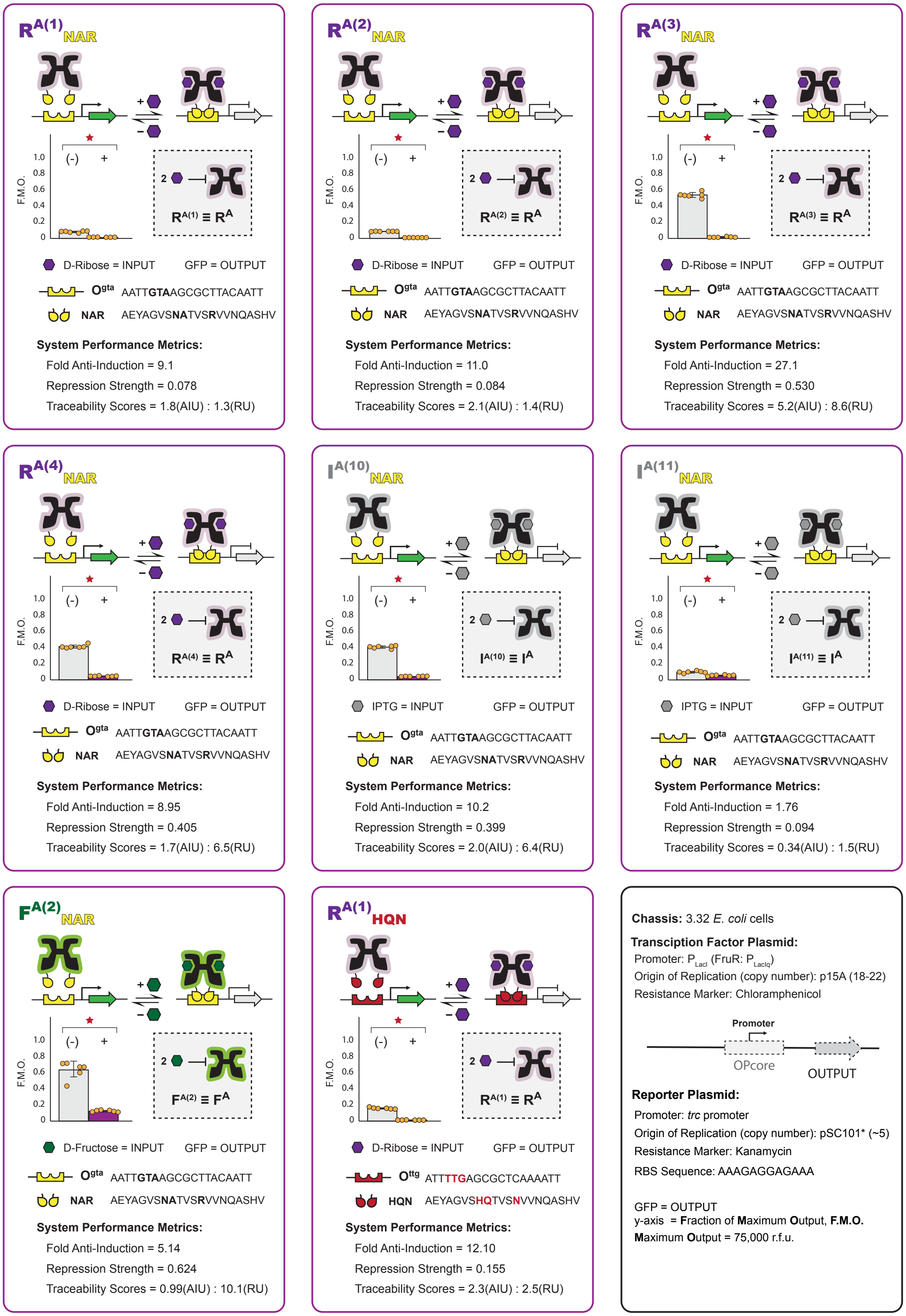

AIU = anti-induction units, relative to I^{+}_{YQR} | O^1 unit operation

RU = repression units, relative to I^{+}_{YQR} | O^1 unit operation

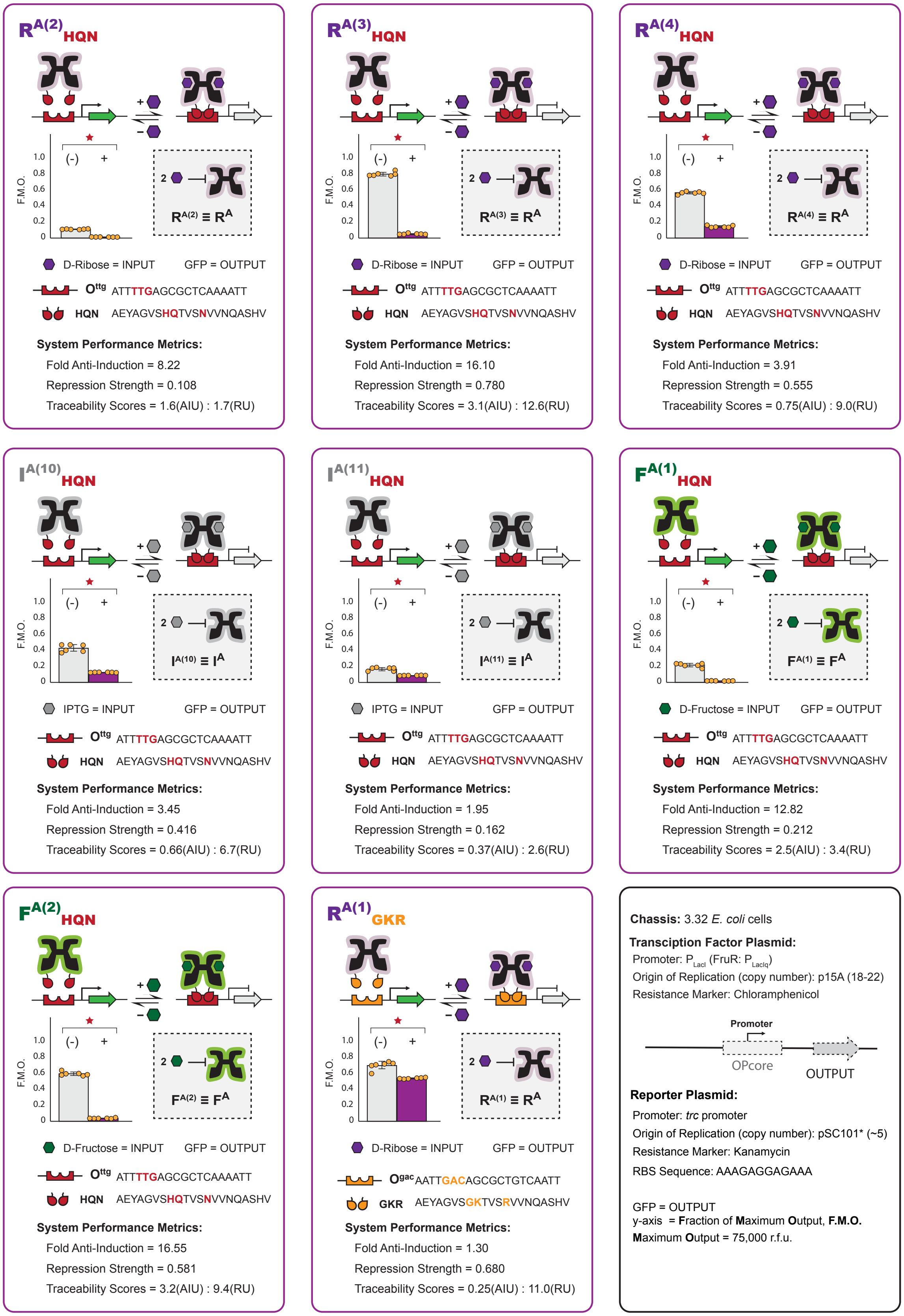

AIU = anti-induction units, relative to I^{+}_{YQR} | O^{1} unit operation

RU = repression units, relative to I^{+}_{YQR} | O^{1} unit operation

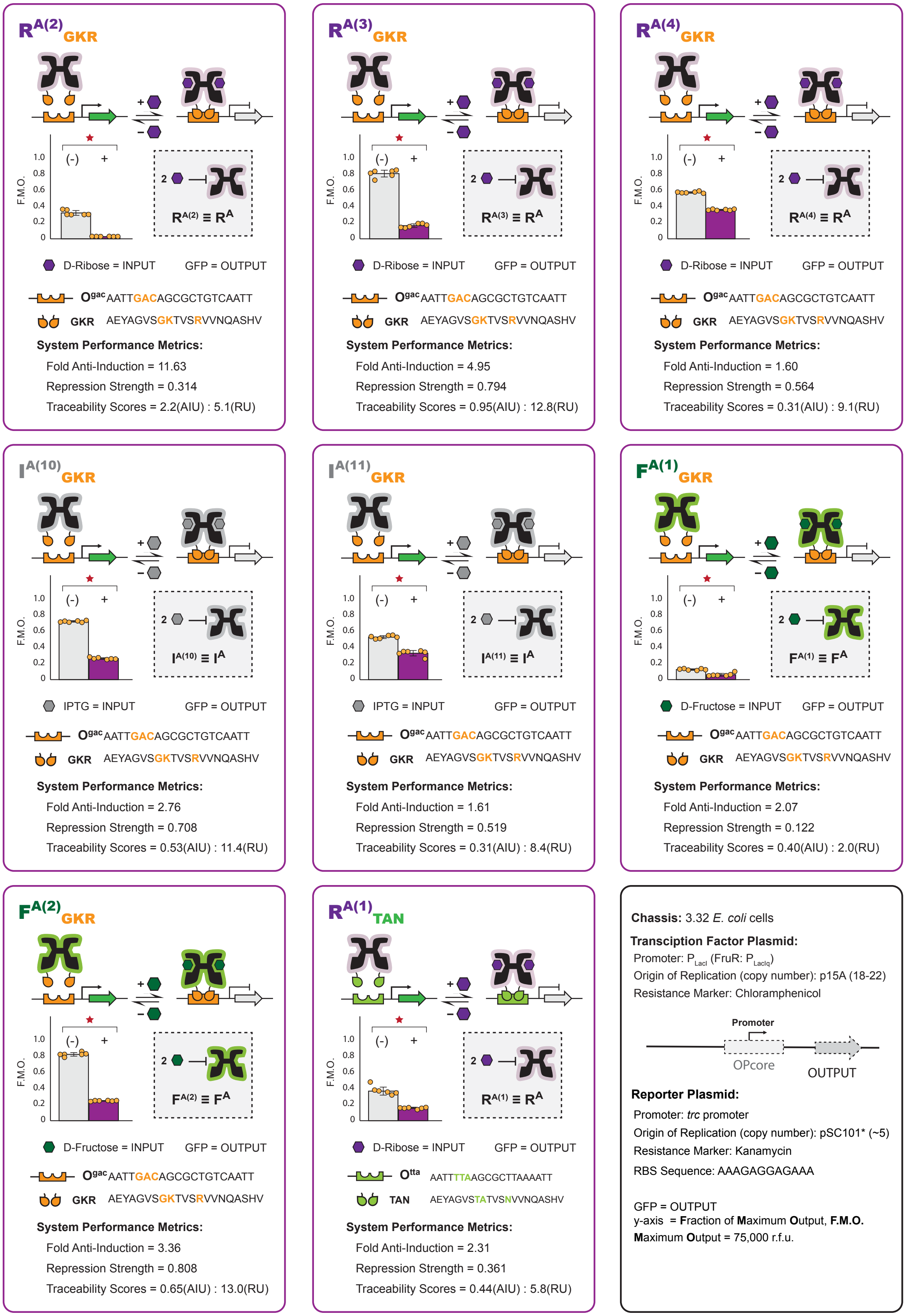

AIU = anti-induction units, relative to I^{+}_{YQR} | O^{1} unit operation

RU = repression units, relative to I^{+}_{YQR} | O^{1} unit operation

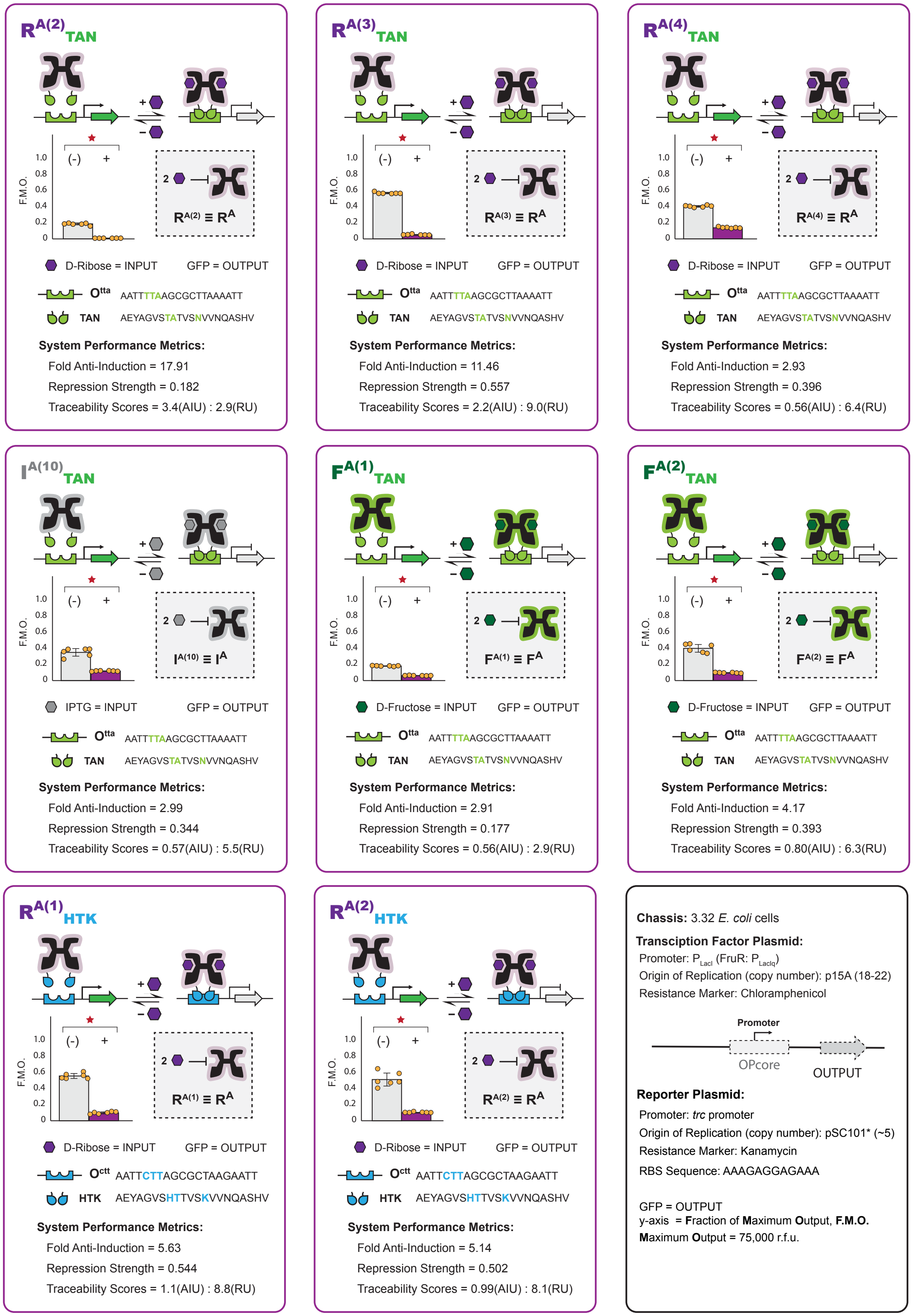

AIU = anti-induction units, relative to I^{+}_{YQR} | O^{1} unit operation

RU = repression units, relative to I^{+}_{YQR} | O^{1} unit operation

$R^{A(3)}_{HTK}$

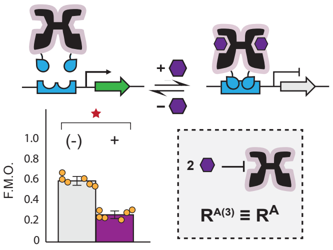

D-Ribose = INPUT GFP = OUTPUT

O^{ctt} AATTCTTAGCGCTAAGAATT

HTK AEYAGVSHTTVSKVVNQASHV

System Performance Metrics:

Fold Anti-Induction = 2.24

Repression Strength = 0.585

Traceability Scores = 0.43(AIU) : 9.4(RU)

$R^{A(4)}_{HTK}$

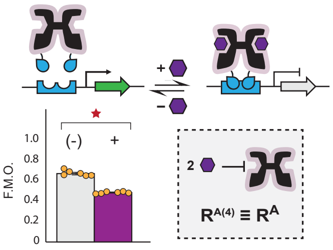

D-Ribose = INPUT GFP = OUTPUT

O^{ctt} AATTCTTAGCGCTAAGAATT

HTK AEYAGVSHTTVSKVVNQASHV

System Performance Metrics:

Fold Anti-Induction = 1.38

Repression Strength = 0.654

Traceability Scores = 0.27(AIU) : 10.5(RU)

$I^{A(10)}_{HTK}$

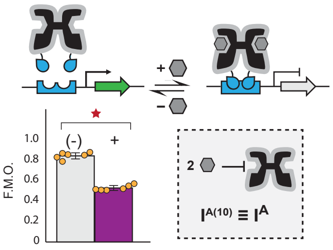

IPTG = INPUT GFP = OUTPUT

O^{ctt} AATTCTTAGCGCTAAGAATT

HTK AEYAGVSHTTVSKVVNQASHV

System Performance Metrics:

Fold Anti-Induction = 1.59

Repression Strength = 0.823

Traceability Scores = 0.31(AIU) : 13.3(RU)

$I^{A(11)}_{HTK}$

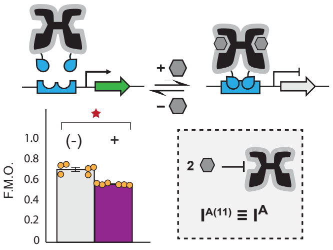

IPTG = INPUT GFP = OUTPUT

O^{ctt} AATTCTTAGCGCTAAGAATT

HTK AEYAGVSHTTVSKVVNQASHV

System Performance Metrics:

Fold Anti-Induction = 1.25

Repression Strength = 0.695

Traceability Scores = 0.24(AIU) : 11.2(RU)

$F^{A(2)}_{HTK}$

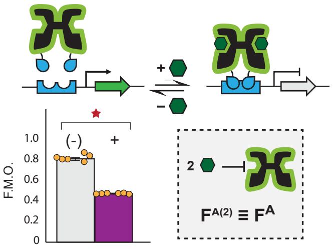

D-Fructose = INPUT GFP = OUTPUT

O^{ctt} AATTCTTAGCGCTAAGAATT

HTK AEYAGVSHTTVSKVVNQASHV

System Performance Metrics:

Fold Anti-Induction = 1.7

Repression Strength = 0.796

Traceability Scores = 0.33(AIU) : 12.8(RU)

$R^{A(1)}_{KSL}$

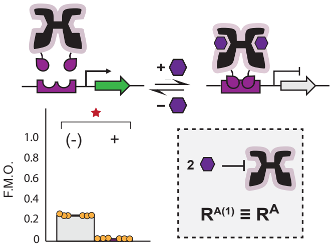

D-Ribose = INPUT GFP = OUTPUT

O^{agg} ATTAGGAGCGCTCCTAATT

KSL AEYAGVSKSTVSLVVNQASHV

System Performance Metrics:

Fold Anti-Induction = 10.55

Repression Strength = 0.246

Traceability Scores = 2.0(AIU) : 4.0(RU)

$R^{A(2)}_{KSL}$

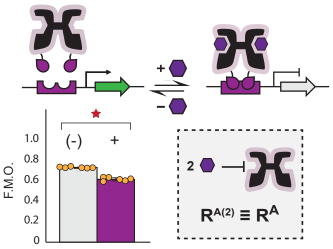

D-Ribose = INPUT GFP = OUTPUT

O^{agg} ATTAGGAGCGCTCCTAATT

KSL AEYAGVSKSTVSLVVNQASHV

System Performance Metrics:

Fold Anti-Induction = 1.25

Repression Strength = 0.749

Traceability Scores = 0.24(AIU) : 12.1(RU)

$R^{A(3)}_{KSL}$

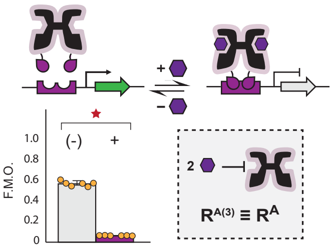

D-Ribose = INPUT GFP = OUTPUT

O^{agg} ATTAGGAGCGCTCCTAATT

KSL AEYAGVSKSTVSLVVNQASHV

System Performance Metrics:

Fold Anti-Induction = 9.10

Repression Strength = 0.558

Traceability Scores = 1.8(AIU) : 9.0(RU)

Chassis: 3.32 *E. coli* cells

Transciption Factor Plasmid:

Promoter: P_{LacI} (FruR: P_{LacIq})

Origin of Replication (copy number): p15A (18-22)

Resistance Marker: Chloramphenicol

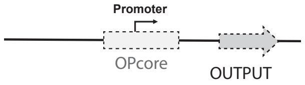

Reporter Plasmid:

Promoter: *trc* promoter

Origin of Replication (copy number): pSC101* (~5)

Resistance Marker: Kanamycin

RBS Sequence: AAAGAGGAGAAA

GFP = OUTPUT

y-axis = **F**raction of **M**aximum **O**utput, **F.M.O.**

Maximum **O**utput = 75,000 r.f.u.

AIU = anti-induction units, relative to I^{+}_{YQR} | O^1 unit operation

RU = repression units, relative to I^{+}_{YQR} | O^1 unit operation

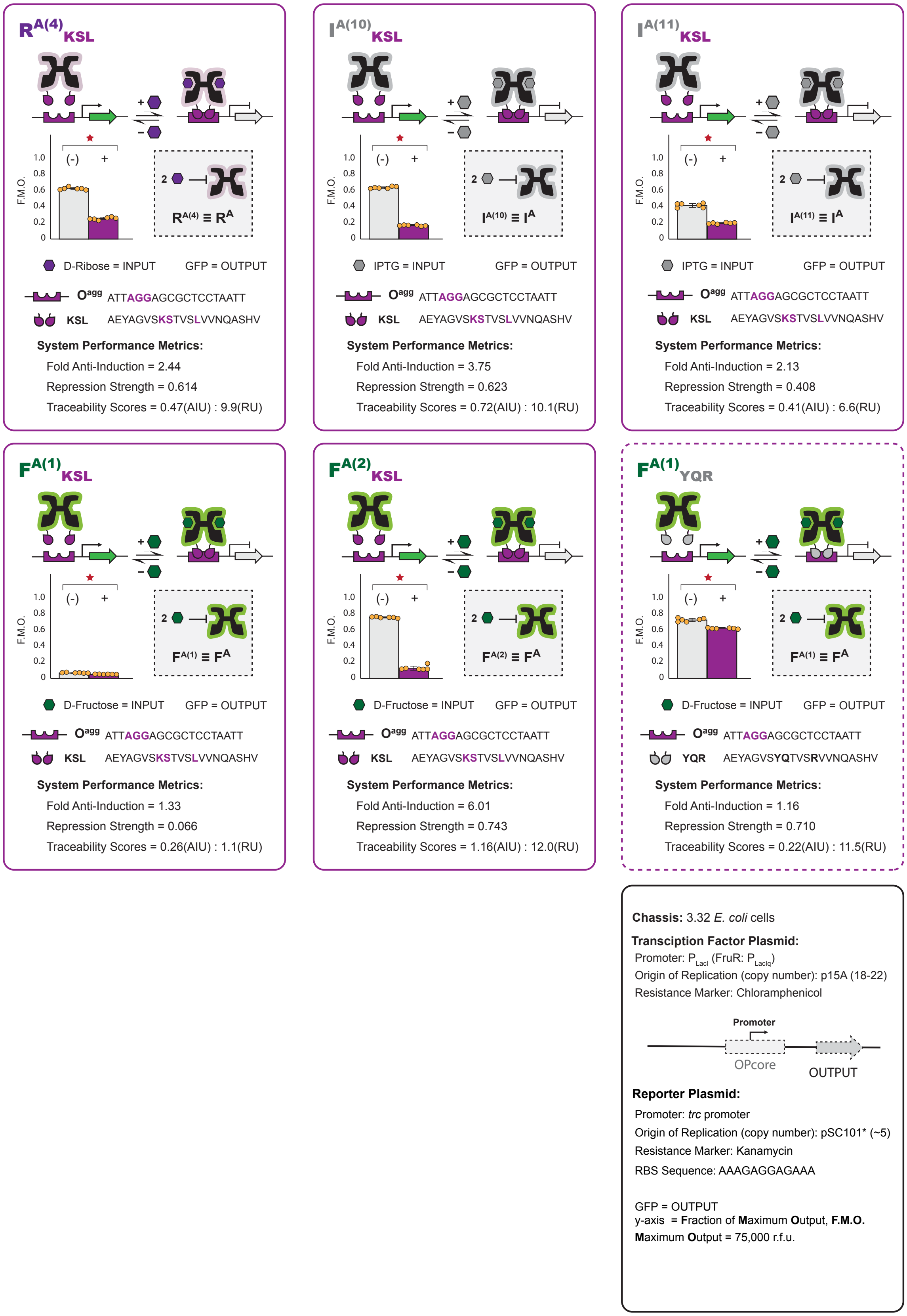

AIU = anti-induction units, relative to I^{+}_{YQR} | O^{1} unit operation

RU = repression units, relative to I^{+}_{YQR} | O^{1} unit operation

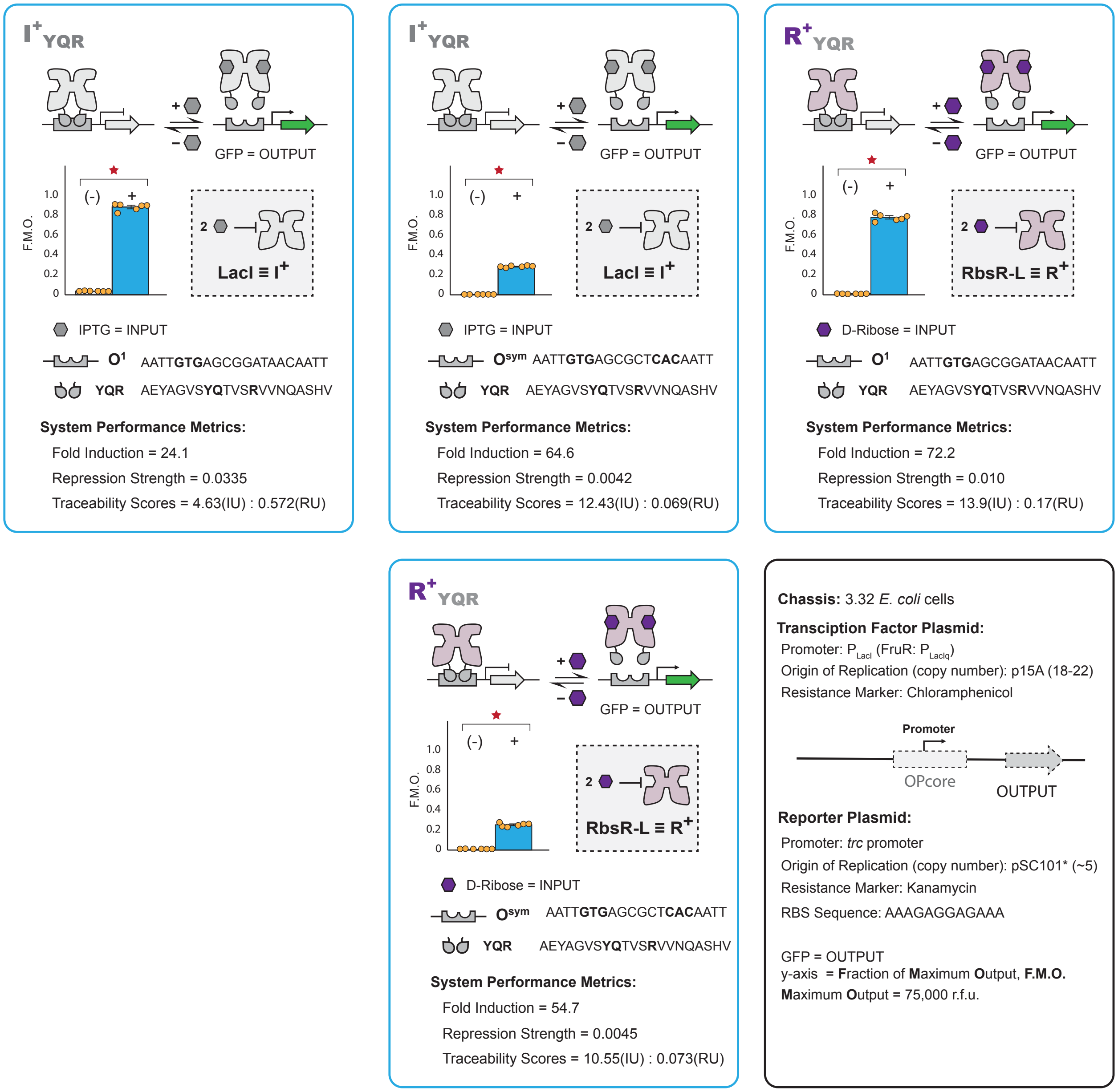

IU = induction units, relative to I^{+}_{YQR} | O^{1} unit operation

RU = repression units, relative to I^{+}_{YQR} | O^{1} unit operation

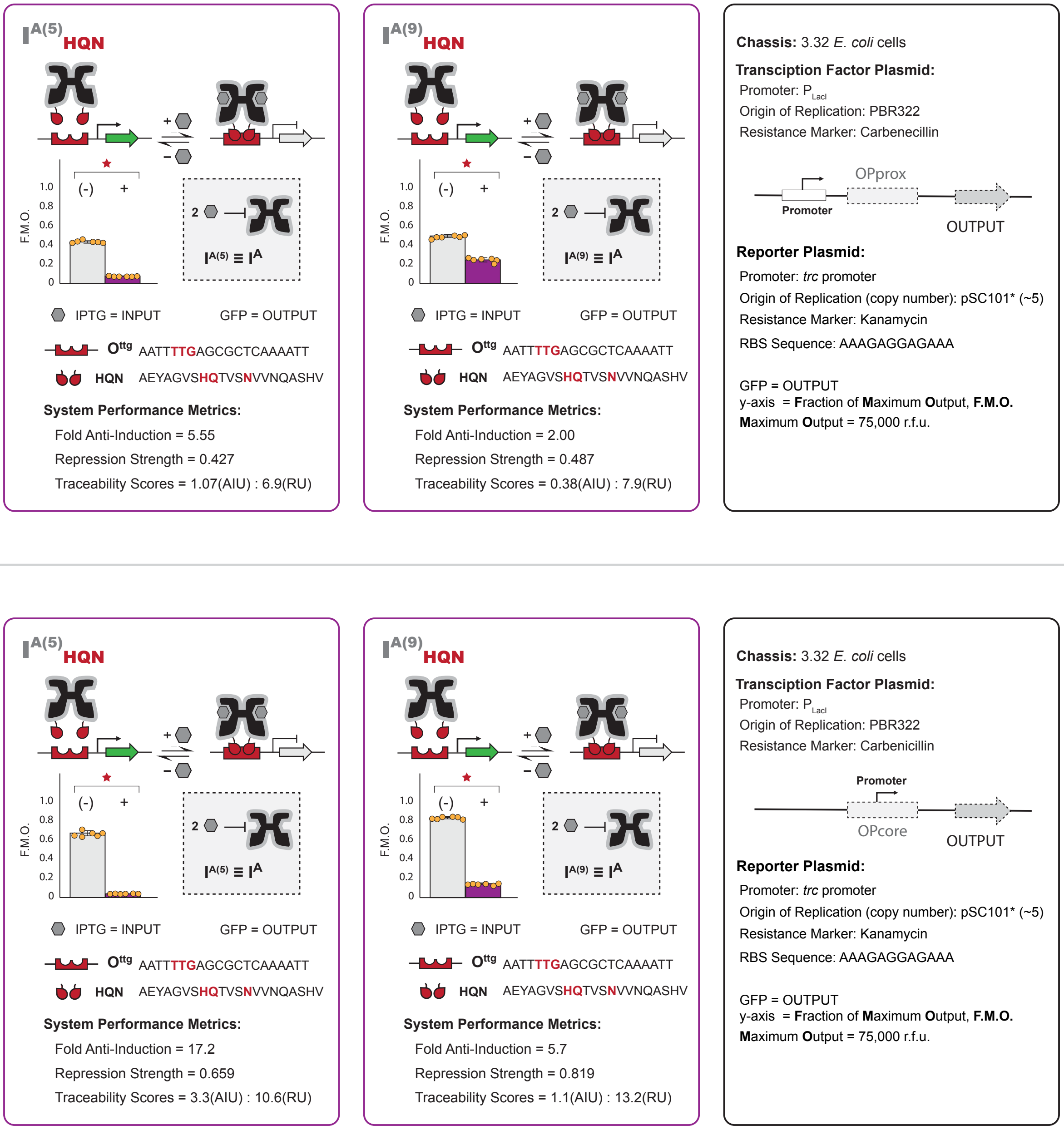

AIU = anti-indution units, relative to I^{+}_{YQR} | O^{1} unit operation

RU = repression units, relative to I^{+}_{YQR} | O^{1} unit operation

Supplementary Figure 8: Performance cards for engineered transcriptional repressors and anti-repressors. Each card corresponds to a biological unit operation (BUO), consisting of (i) a regulatory core and (ii) a DNA-binding domain paired with (iii) an operator, at a given position (core or proximal). Vertical-bars display normalized mean fluorescence intensity (defining the measure of centre), standardized to the maximum fluorescence output observed (75,000 r.f.u.), giving fraction of maximum output, F.M.O, ±1 S.D. Left bars show values without ligand, whereas the right is value in the presence of ligand. Red stars signify statistical significance (α = 0.001) in a Student's two-tailed t-test for n = 6 biological replicates. Dots are shown for individual replicates. Performance metrics can be calculated, see **Supplementary Note 4**, namely: fold induction or anti-induction (fold change), repression strength (expression in the (-) ligand state), and traceability scores, which standardize fold change and repression strength to the I^{+}_{YQR} | $O^{1(proximal)}$ BUO reference. Additionally shown is a phenotype cartoon and operator and DBD sequences. "Backs" of cards display information on BUO context, namely the cell chassis, reporter plasmid type (including core or proximal operator position), and specifications of genetic elements for the reporter and transcription factor

plasmids. Solid outlines indicate cognate interactions between operator and DBD, while dashed lines indicate non-cognate (promiscuous) interactions. Blue outlines indicate a repressor phenotype, while purple outlines indicate an anti-repressor phenotype.

Supplementary Note 4:

Metrics for X^{A}_{ADR} Anti-Repressors. The Fraction of Maximum Output (F.M.O.) ≡ [GFP/OD_{600}] / [Maximum Lac_{null} Value**], where (i) F.M.O. repression is the system without ligand, and (ii) F.M.O. anti-repressor is the system with ligand. Repression Strength ≡ F.M.O. normalized output without ligand, and Fold Anti-Repression ≡ FS, such that:

$$\mathrm{FAI} = \frac{(\mathrm{F.M.O.\ Repression})}{(\mathrm{F.M.O.\ Anti-Repression})} \tag{1}$$

Part 1 of the traceability score is given in terms of the anti-repressed state (where AIU = Anti-Induction Units) such that the AIU traceability scores were calculated as follows:

$$\mathrm{AIU\ Traceability\ Score} = \frac{\mathrm{FAI}\left(\mathrm{X}^{\mathrm{A}}_{\mathrm{ADR}}\ |\mathrm{OP}\right)}{\mathrm{FI}(\mathrm{I}^{+}_{\mathrm{YQR}}\ |\ \mathrm{O}^{1})} \tag{2}$$

$$\mathrm{AIU\ Reference\ Score} = \frac{\mathrm{FI}(\mathrm{I}^{+}_{\mathrm{YQR}}|\mathrm{O}^{1})}{\mathrm{FI}(\mathrm{I}^{+}_{\mathrm{YQR}}|\mathrm{O}^{1})} = 1 \tag{3}$$

Part 2 of the traceability score is given in terms of the repressed state (where RU = Repression Units) such that the RU traceability scores for an anti-repressor were calculated as follows:

$$\mathrm{RU\ Traceability\ Score} = \frac{\mathrm{F.M.O.\ Repression}\left(\mathrm{X}^{\mathrm{A}}_{\mathrm{ADR}}\ |\mathrm{OP}\right)}{\mathrm{F.M.O.\ Repression}\ (\mathrm{I}^{+}_{\mathrm{YQR}}\ |\ \mathrm{O}^{1})} \tag{4}$$

$$\mathrm{RU\ Reference\ Score} = \frac{\mathrm{F.M.O.\ Repression}\ (\mathrm{I}^{+}_{\mathrm{YQR}}|\mathrm{O}^{1})}{\mathrm{F.M.O.\ Repression}\ (\mathrm{I}^{+}_{\mathrm{YQR}}|\mathrm{O}^{1})} = 1 \tag{5}$$

**Maximum Lac_{null} value = 75,000 relative fluorescence units (rfu), OD_{600} normalized

The reduced dynamic range observed in ~13% of the BUO in the proximal position (relative to the core position) likely resulted from non-competitive binding between a given anti-repressor and RNA polymerase, see **Fig. 3a-b**. Namely, the anti-repressor in the repressed state (*i.e.*, without ligand) is not displaced by RNA polymerase resulting in diminished OUTPUT relative to the core unit operation. This mechanism at the proximal position can also result in greater (relative) leakiness in the anti-induced state (*i.e.*, with ligand). In general, one (or both) events result in an observed reduction in the dynamic range at the proximal position, see **Fig. 5c-d**.

Supplementary Figure 9

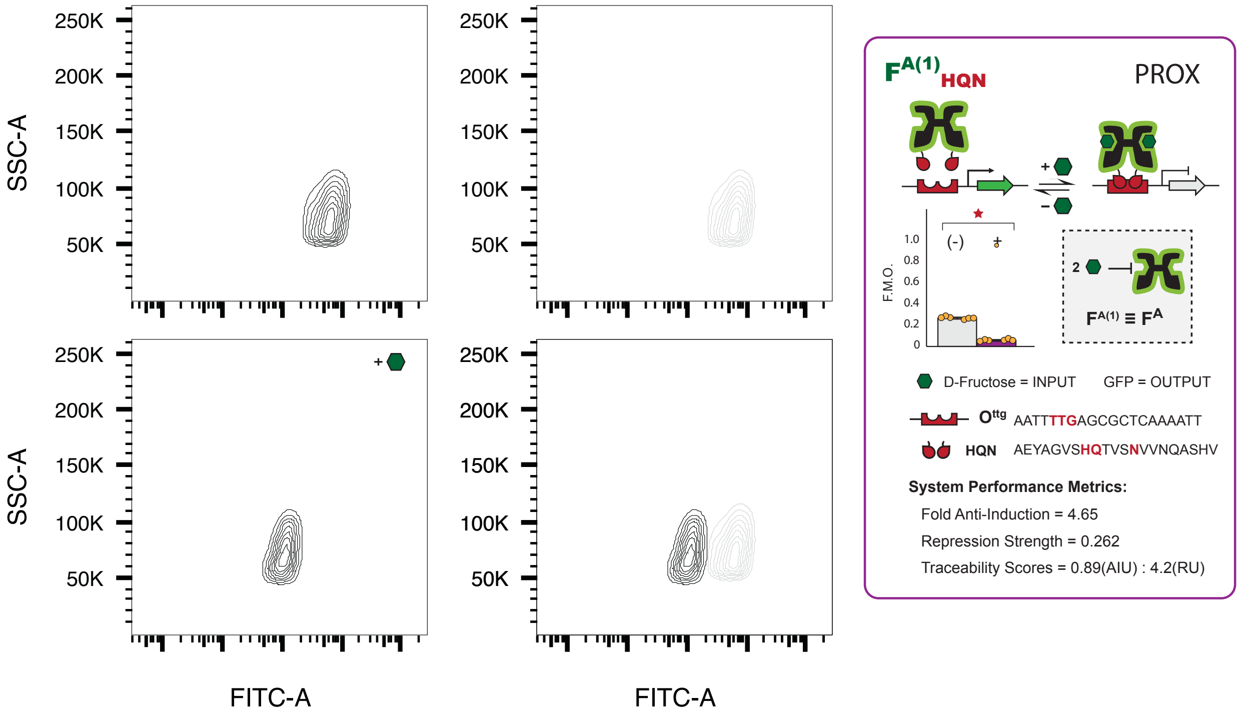

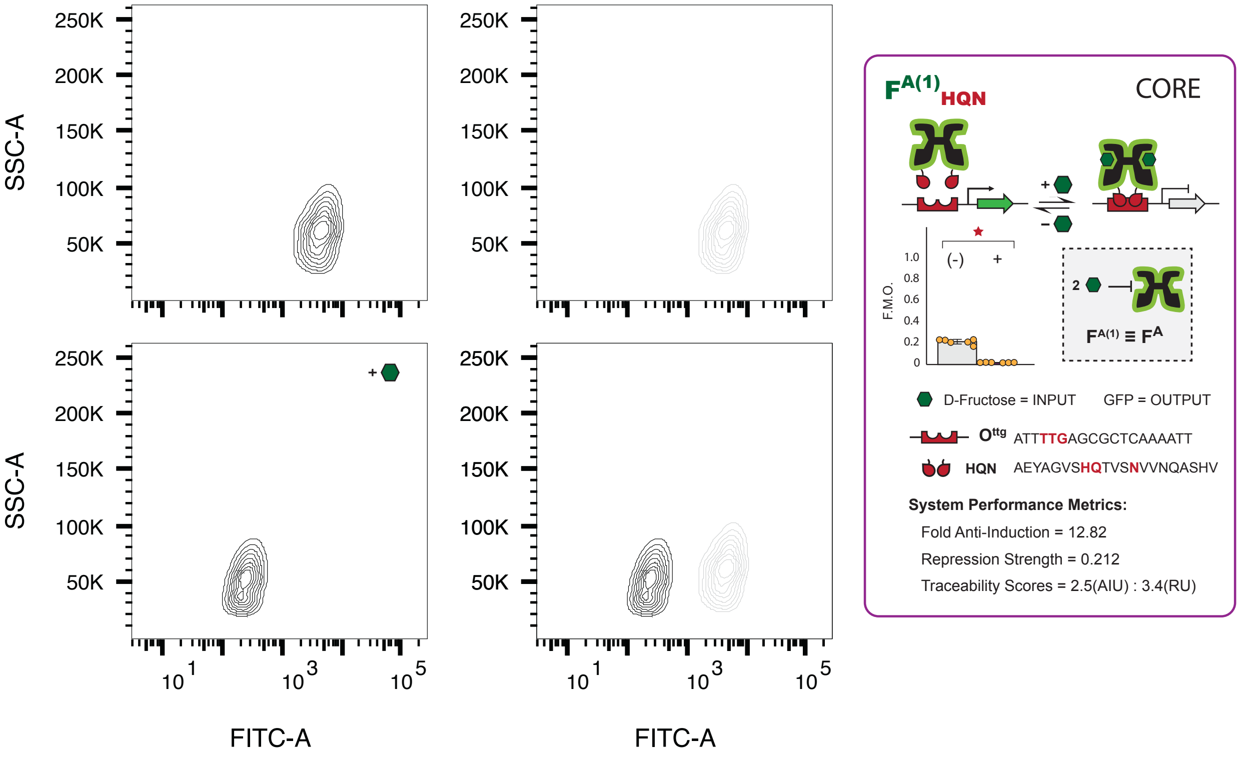

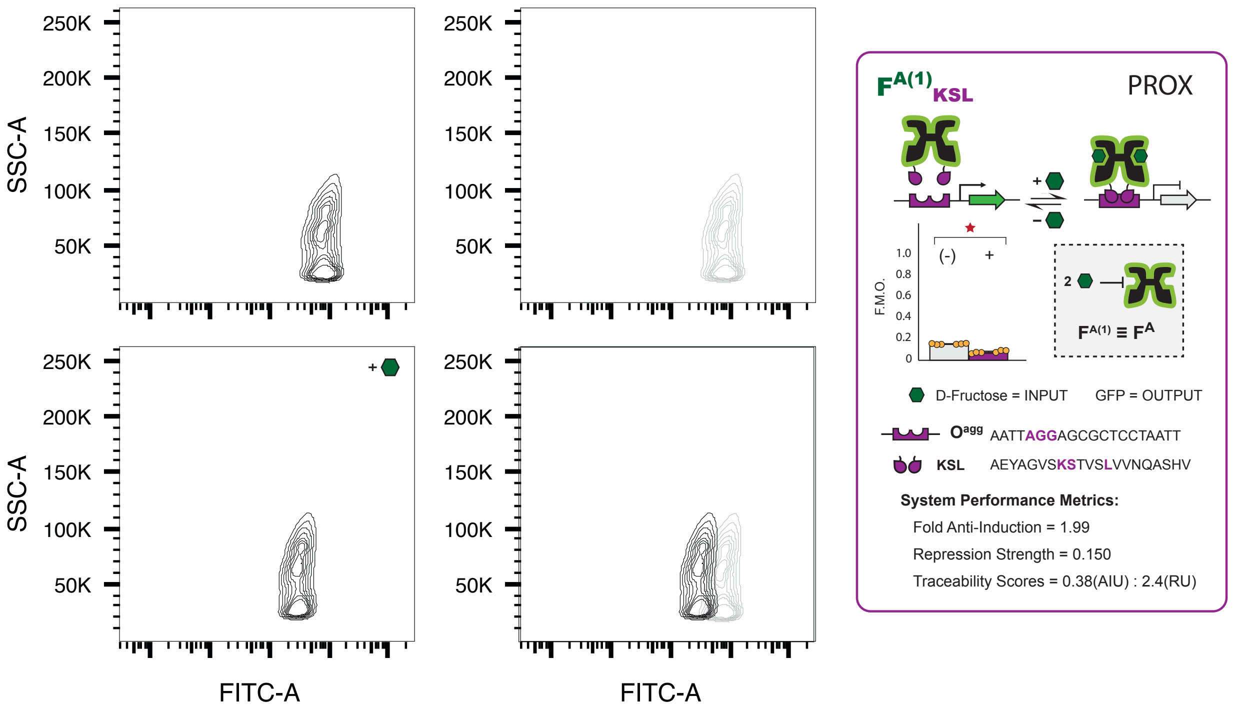
250K
200K
150K
100K
50K
SSC-A
FITC-A
FITC-A
$F^{A(1)}_{KSL}$
PROX
F.M.O.
1.0
0.8
0.6
0.4
0.2
0
(-)
+
2
$F^{A(1)} \equiv F^{A}$
D-Fructose = INPUT
GFP = OUTPUT
O^{agg} AATTAGGAGCGCTCCTAATT
KSL AEYAGVSKSTVSLVVNQASHV
System Performance Metrics:
Fold Anti-Induction = 1.99
Repression Strength = 0.150
Traceability Scores = 0.38(AIU) : 2.4(RU)

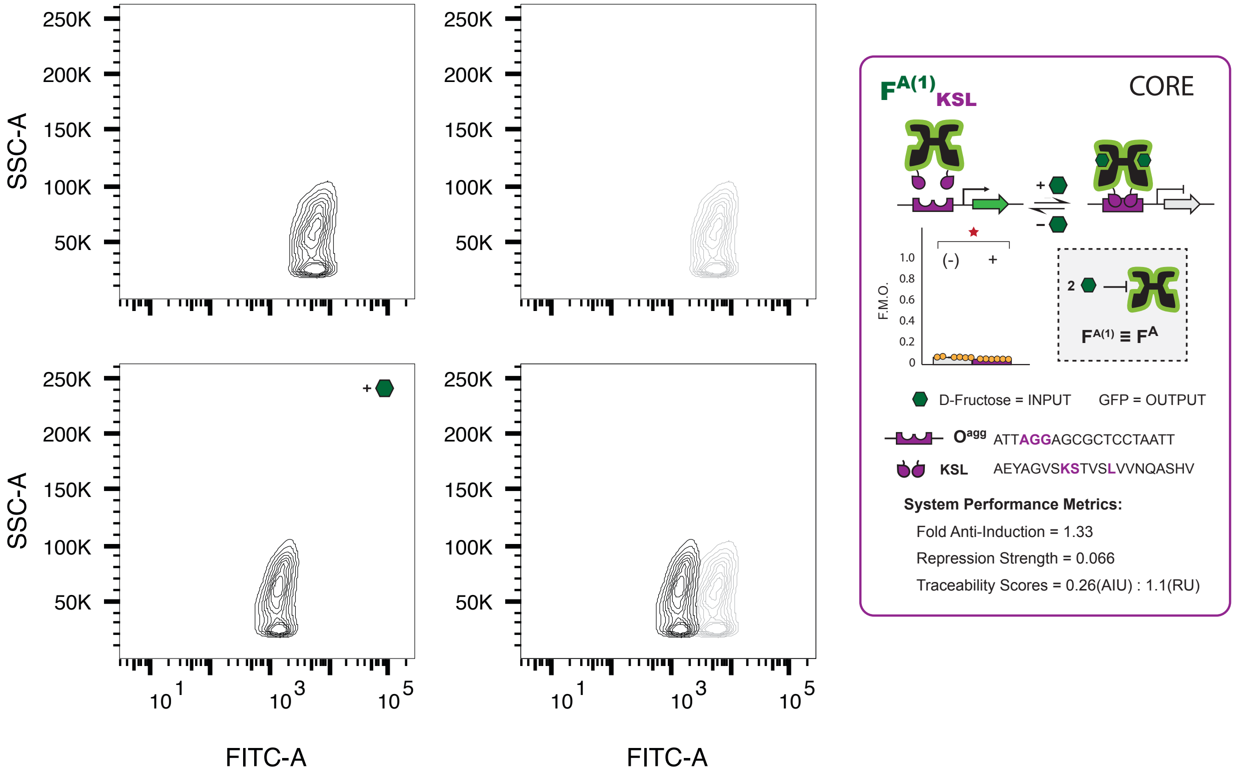
250K
200K
150K
100K
50K
SSC-A
10^1
10^3
10^5
FITC-A
FITC-A
$F^{A(1)}_{KSL}$
CORE
F.M.O.
1.0
0.8
0.6
0.4
0.2
0
(-)
+
2
$F^{A(1)} \equiv F^{A}$
D-Fructose = INPUT
GFP = OUTPUT
O^{agg} ATTAGGAGCGCTCCTAATT
KSL AEYAGVSKSTVSLVVNQASHV
System Performance Metrics:
Fold Anti-Induction = 1.33
Repression Strength = 0.066
Traceability Scores = 0.26(AIU) : 1.1(RU)

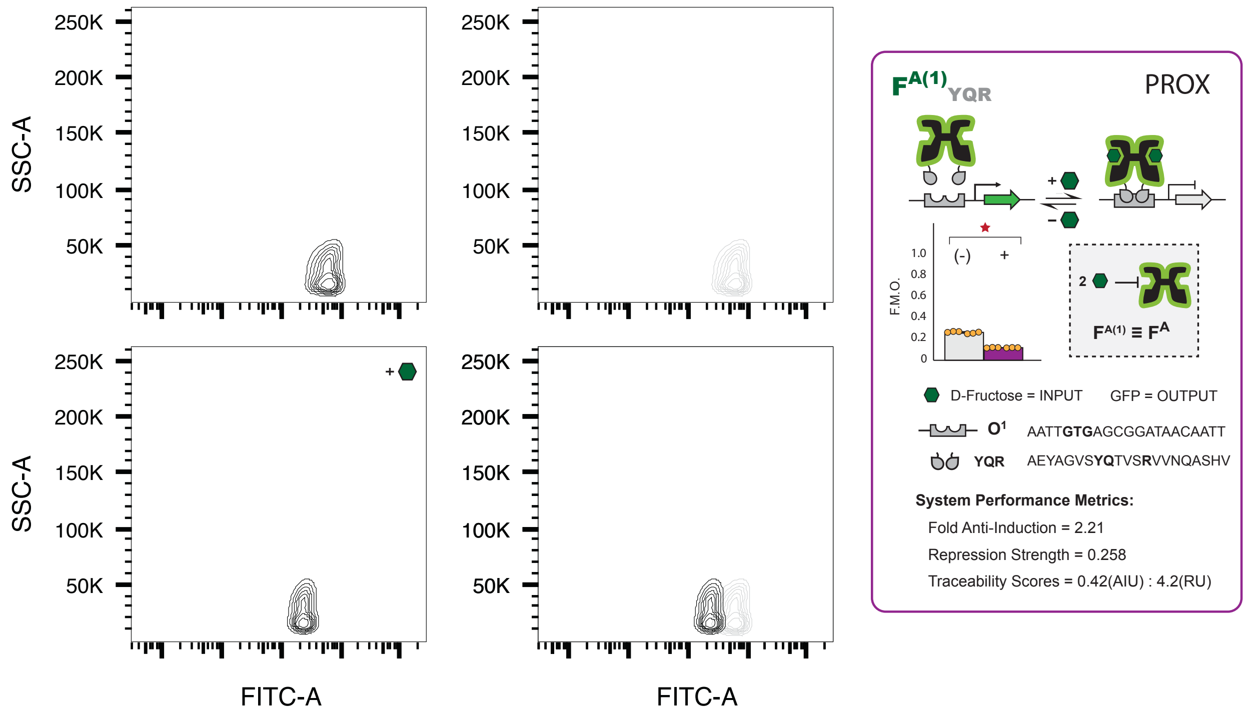

250K
200K
150K
100K
50K
SSC-A
FITC-A
+
F^A(1) YQR
PROX
+
-
1.0
0.8
0.6
0.4
0.2
0
F.M.O.
(-)
+
2
F^A(1) ≡ F^A
D-Fructose = INPUT
GFP = OUTPUT
O1
AATTGTGAGCGGATAACAATT
YQR
AEYAGVSYQTVSRVVNQASHV
System Performance Metrics:
Fold Anti-Induction = 2.21
Repression Strength = 0.258
Traceability Scores = 0.42(AIU) : 4.2(RU)

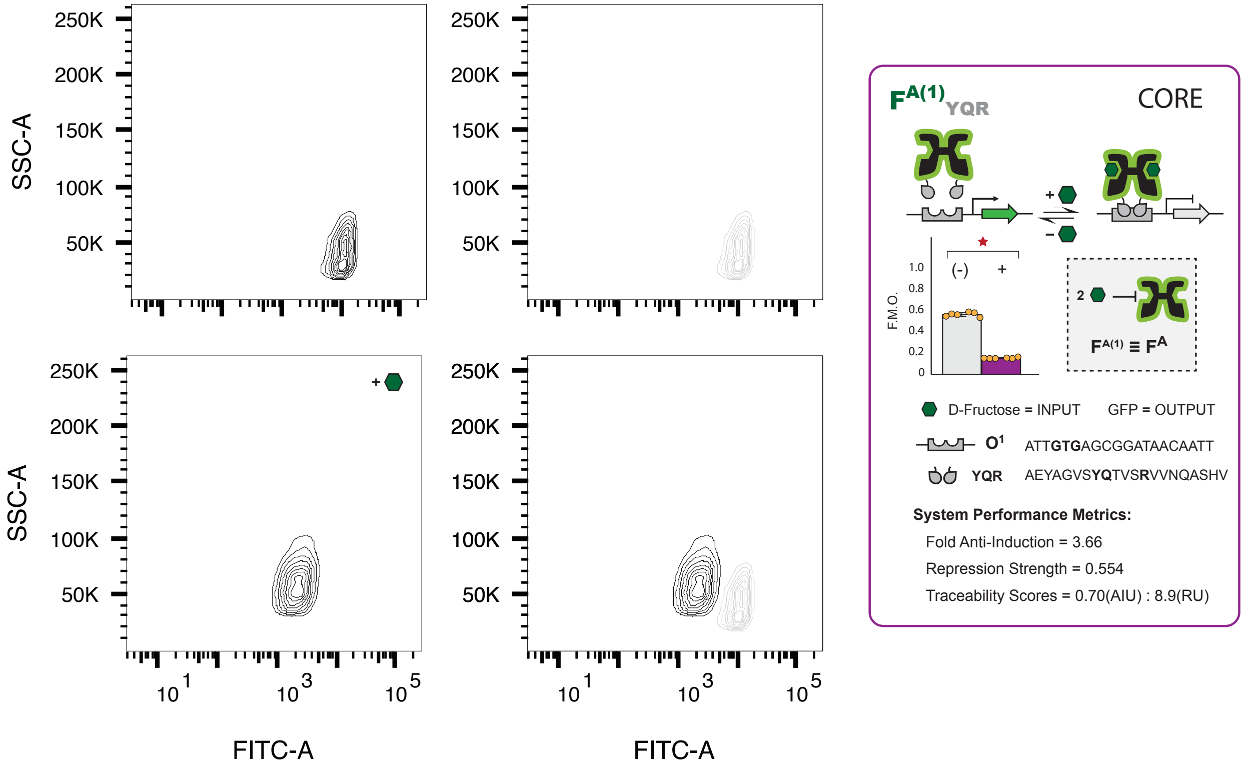

250K
200K
150K
100K
50K
SSC-A
10^1
10^3
10^5
FITC-A
+
F^A(1) YQR
CORE
+
-
1.0
0.8
0.6
0.4
0.2
0
F.M.O.
(-)
+
2
F^A(1) ≡ F^A
D-Fructose = INPUT
GFP = OUTPUT
O1
ATTGTGAGCGGATAACAATT
YQR
AEYAGVSYQTVSRVVNQASHV
System Performance Metrics:
Fold Anti-Induction = 3.66
Repression Strength = 0.554
Traceability Scores = 0.70(AIU) : 8.9(RU)

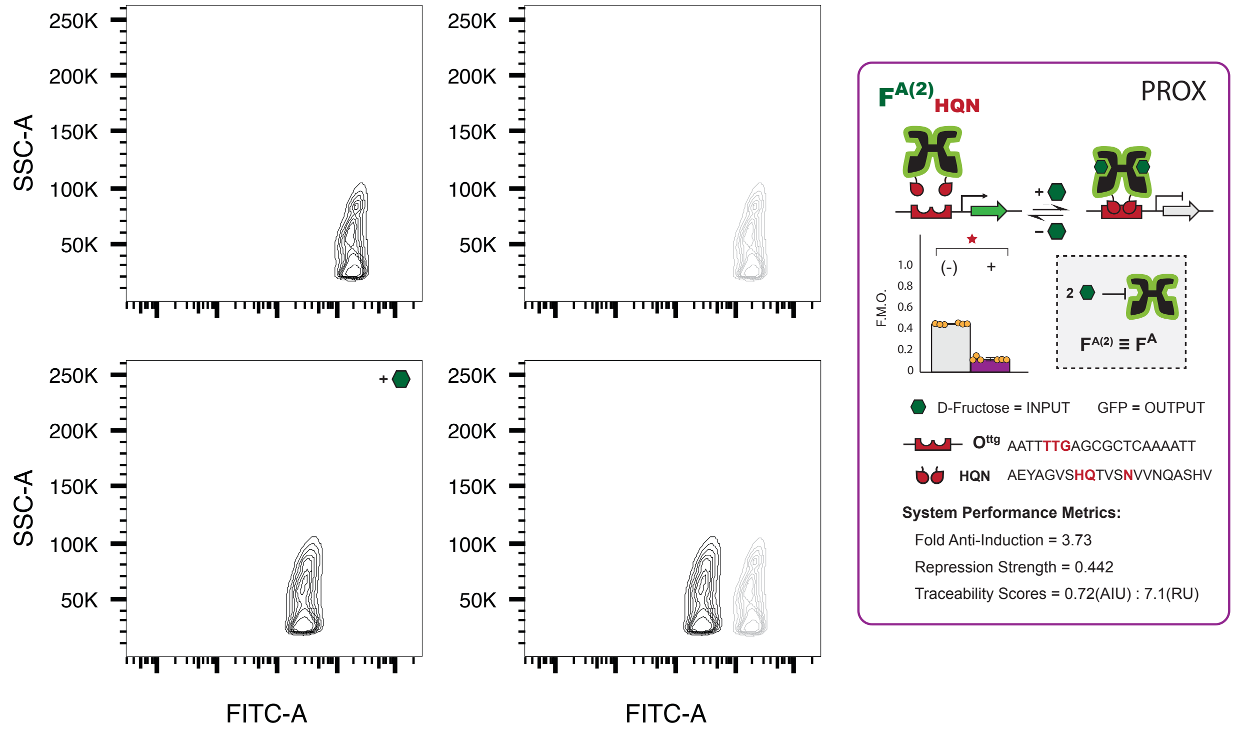
250K
200K
150K
100K
50K
SSC-A
FITC-A
FITC-A
F A(2) HQN
PROX
1.0
0.8
0.6
0.4
0.2
0
F.M.O.
(-)
+
2
FA(2) ≡ FA
D-Fructose = INPUT
GFP = OUTPUT
Ottg AATTTTGAGCGCTCAAAATT
HQN AEYAGVSHQTVSNVVNQASHV
System Performance Metrics:
Fold Anti-Induction = 3.73
Repression Strength = 0.442
Traceability Scores = 0.72(AIU) : 7.1(RU)

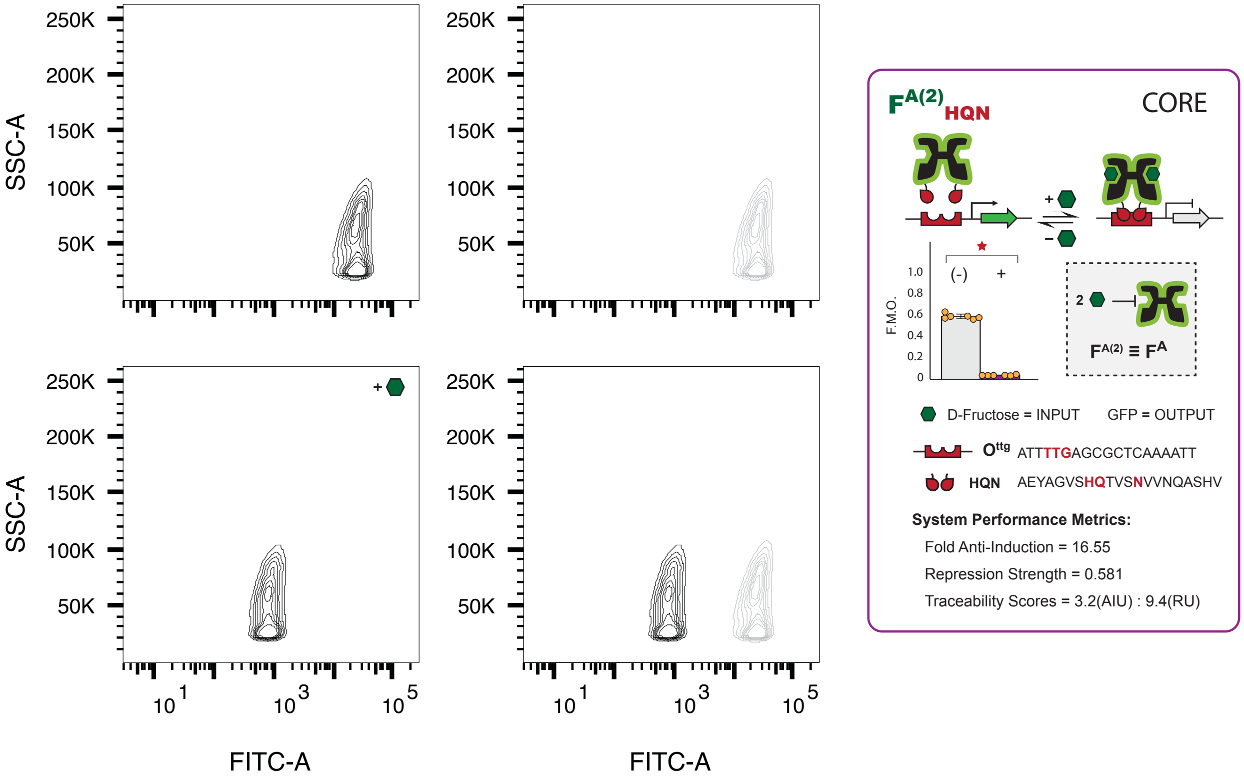
250K
200K
150K
100K
50K
SSC-A
10 1
10 3
10 5
FITC-A
FITC-A
F A(2) HQN
CORE
1.0
0.8
0.6
0.4
0.2
0
F.M.O.
(-)
+
2
FA(2) ≡ FA
D-Fructose = INPUT
GFP = OUTPUT
Ottg ATTTTGAGCGCTCAAAATT
HQN AEYAGVSHQTVSNVVNQASHV
System Performance Metrics:
Fold Anti-Induction = 16.55
Repression Strength = 0.581
Traceability Scores = 3.2(AIU) : 9.4(RU)

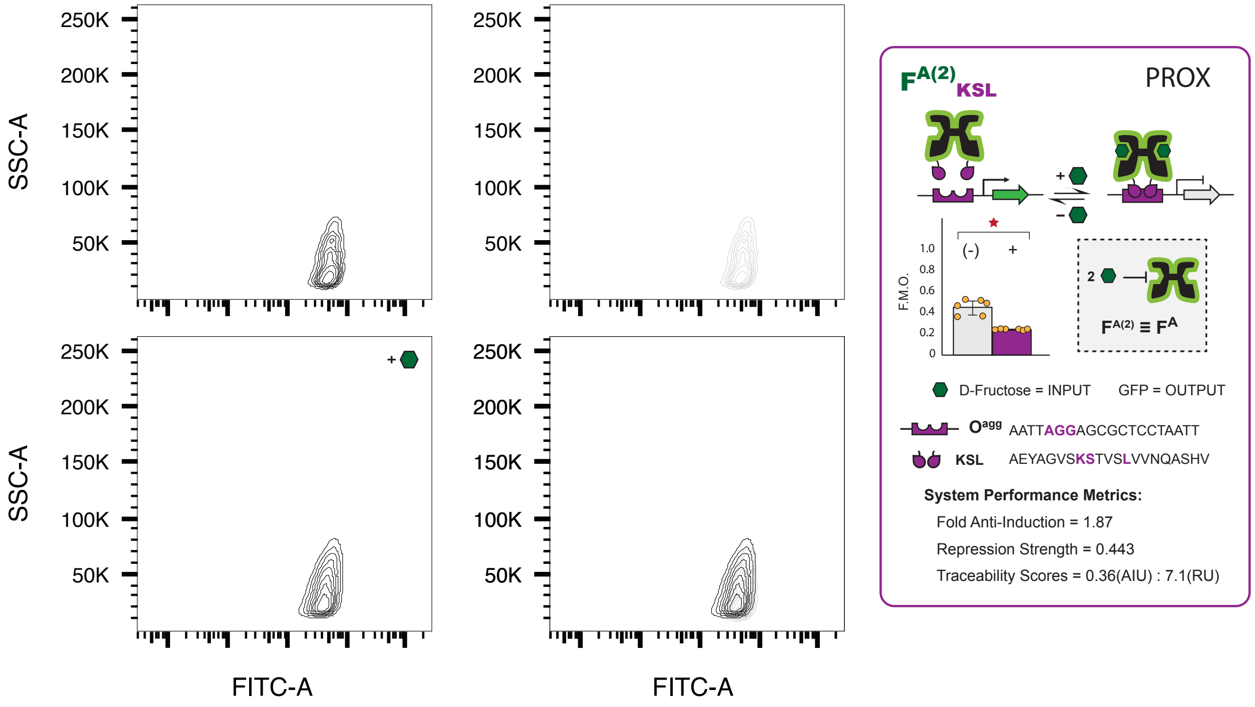
SSC-A
250K
200K
150K
100K
50K
FITC-A
$F^{A(2)}_{KSL}$
PROX
F.M.O.
1.0
0.8
0.6
0.4
0.2
0
(-)
+
2
$F^{A(2)} \equiv F^{A}$
D-Fructose = INPUT
GFP = OUTPUT
O^{agg} AATTAGGAGCGCTCCTAATT
KSL AEYAGVSKSTVSLVVNQASHV
System Performance Metrics:
Fold Anti-Induction = 1.87
Repression Strength = 0.443
Traceability Scores = 0.36(AIU) : 7.1(RU)

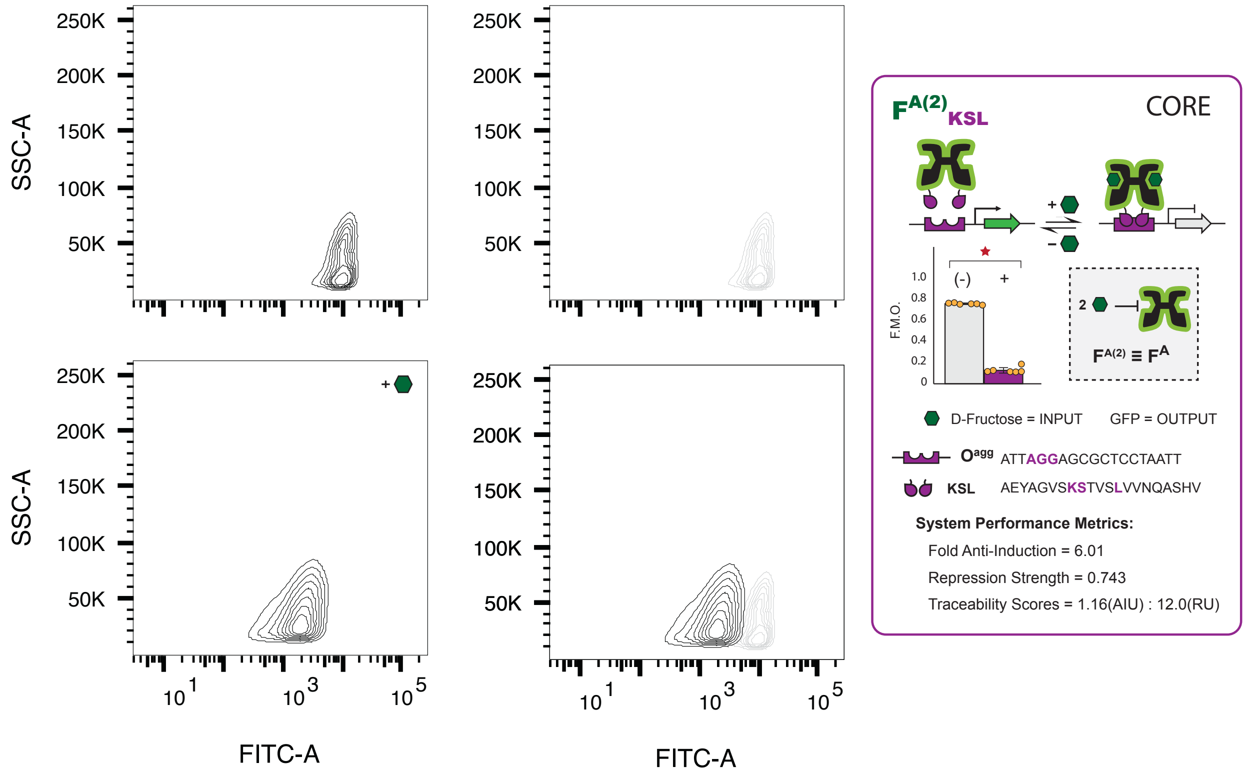
SSC-A
250K
200K
150K
100K
50K
10^1
10^3
10^5
FITC-A
$F^{A(2)}_{KSL}$
CORE
F.M.O.
1.0
0.8
0.6
0.4
0.2
0
(-)
+
2
$F^{A(2)} \equiv F^{A}$
D-Fructose = INPUT
GFP = OUTPUT
O^{agg} ATTAGGAGCGCTCCTAATT
KSL AEYAGVSKSTVSLVVNQASHV
System Performance Metrics:
Fold Anti-Induction = 6.01
Repression Strength = 0.743
Traceability Scores = 1.16(AIU) : 12.0(RU)

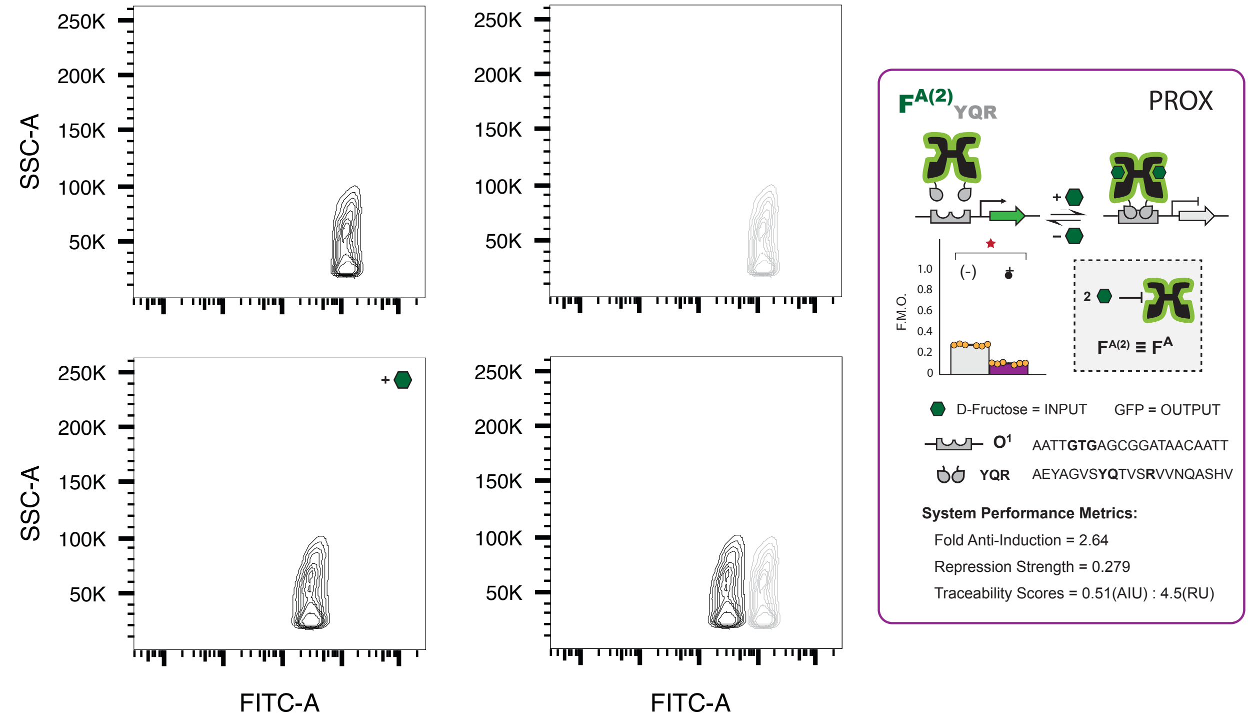

SSC-A
FITC-A
250K
200K
150K
100K
50K
PROX
FA(2)YQR
(-)
F.M.O.
1.0
0.8
0.6
0.4
0.2
0
FA(2) ≡ FA
D-Fructose = INPUT
GFP = OUTPUT
O1
AATTGTGAGCGGATAACAATT
YQR
AEYAGVSYQTVSRVVNQASHV
System Performance Metrics:
Fold Anti-Induction = 2.64
Repression Strength = 0.279
Traceability Scores = 0.51(AIU) : 4.5(RU)

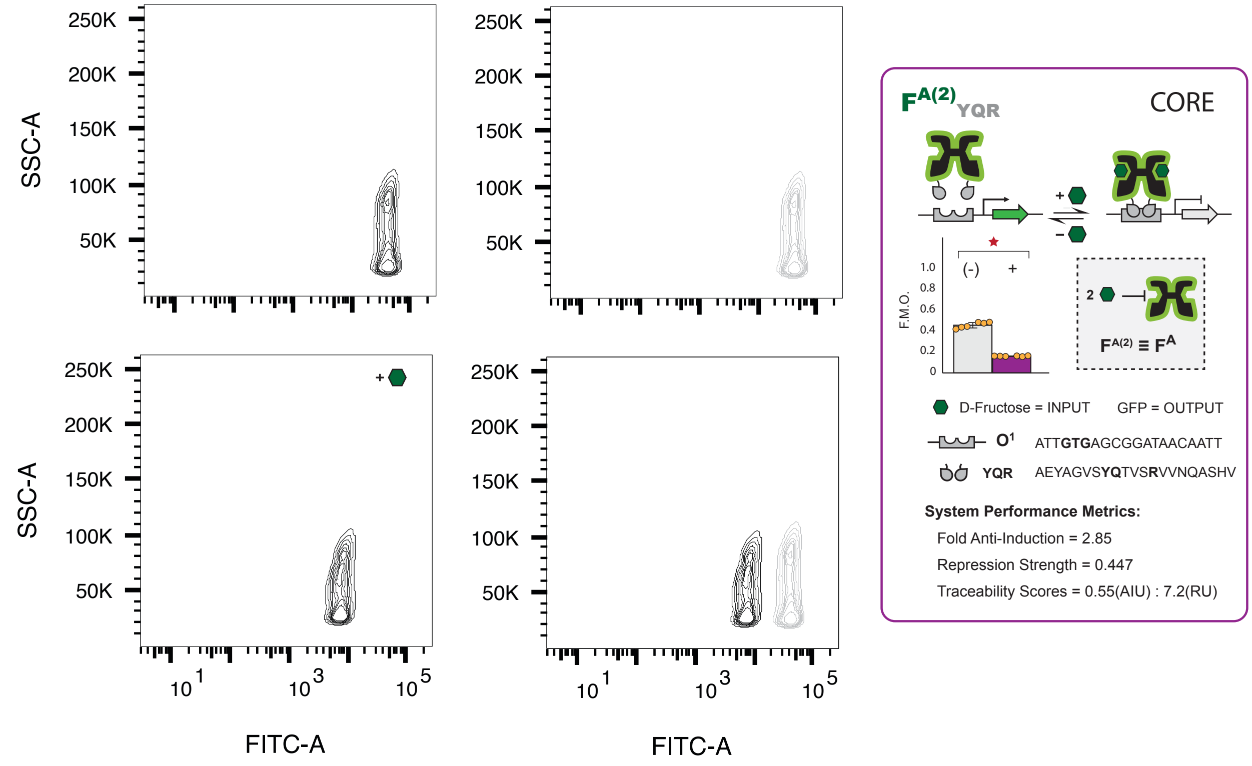

SSC-A
FITC-A
250K
200K
150K
100K
50K
10 1
10 3
10 5
CORE
FA(2)YQR
(-)
+
F.M.O.
1.0
0.8
0.6
0.4
0.2
0
FA(2) ≡ FA
D-Fructose = INPUT
GFP = OUTPUT
O1
ATTGTGAGCGGATAACAATT
YQR
AEYAGVSYQTVSRVVNQASHV
System Performance Metrics:
Fold Anti-Induction = 2.85
Repression Strength = 0.447
Traceability Scores = 0.55(AIU) : 7.2(RU)

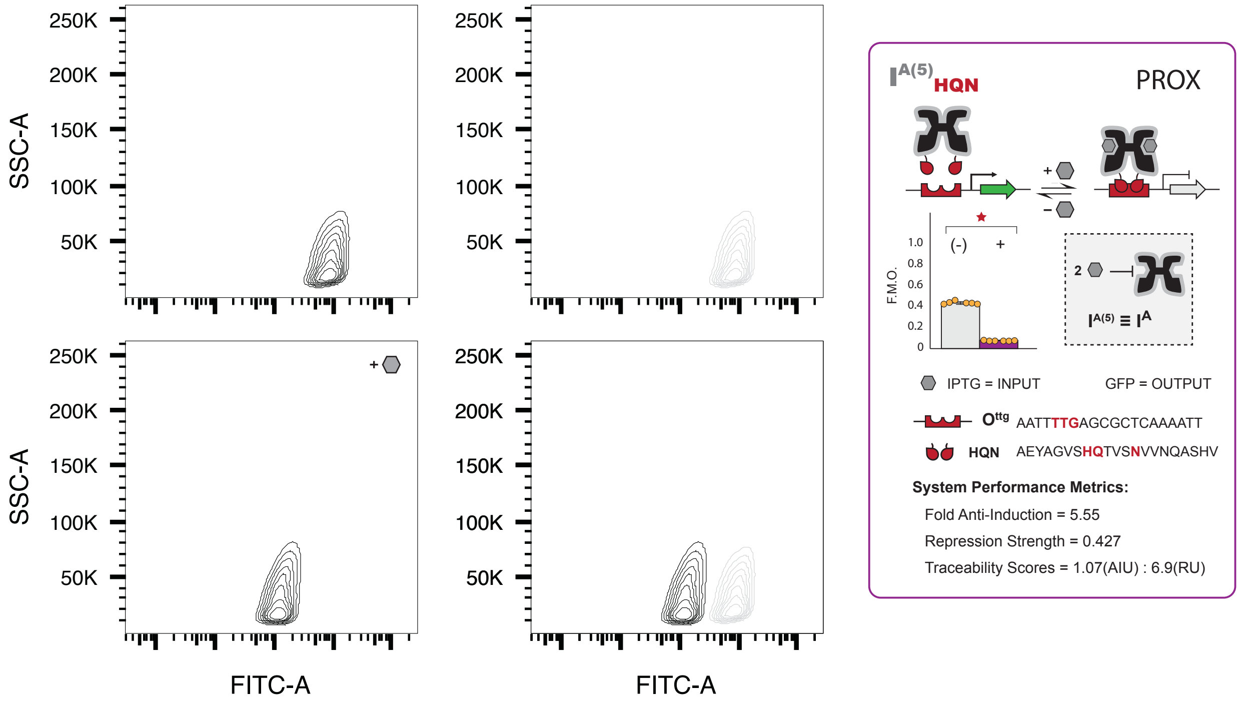

250K
200K
150K
100K
50K
SSC-A
FITC-A
+
I A(5) HQN
PROX
1.0
0.8
0.6
0.4
0.2
0
F.M.O.
(-)
+
2
I A(5) ≡ I A
IPTG = INPUT
GFP = OUTPUT
O ttg AATTTTGAGCGCTCAAAATT
HQN AEYAGVSHQTVSNVVNQASHV
System Performance Metrics:
Fold Anti-Induction = 5.55
Repression Strength = 0.427
Traceability Scores = 1.07(AIU) : 6.9(RU)

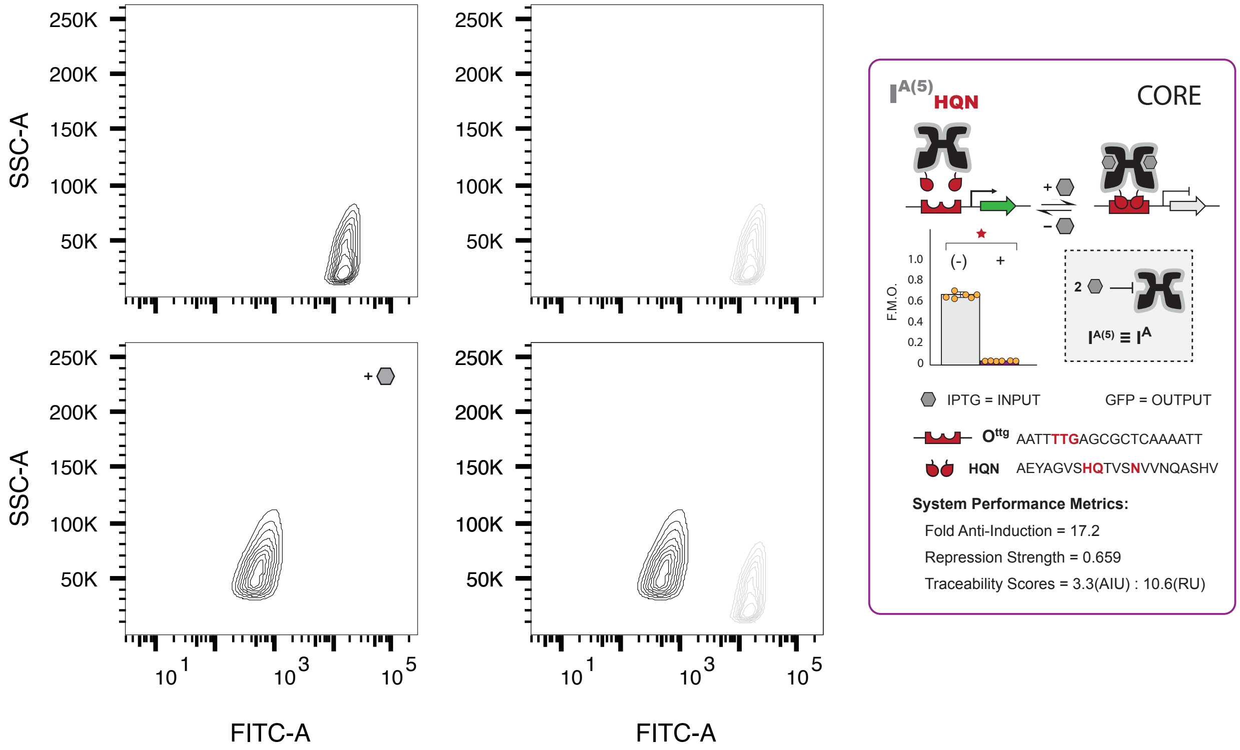

250K
200K
150K
100K
50K
SSC-A
10 1
10 3
10 5
FITC-A
+
I A(5) HQN
CORE
1.0
0.8
0.6
0.4
0.2
0
F.M.O.
(-)
+
2
I A(5) ≡ I A
IPTG = INPUT
GFP = OUTPUT
O ttg AATTTTGAGCGCTCAAAATT
HQN AEYAGVSHQTVSNVVNQASHV
System Performance Metrics:
Fold Anti-Induction = 17.2
Repression Strength = 0.659
Traceability Scores = 3.3(AIU) : 10.6(RU)

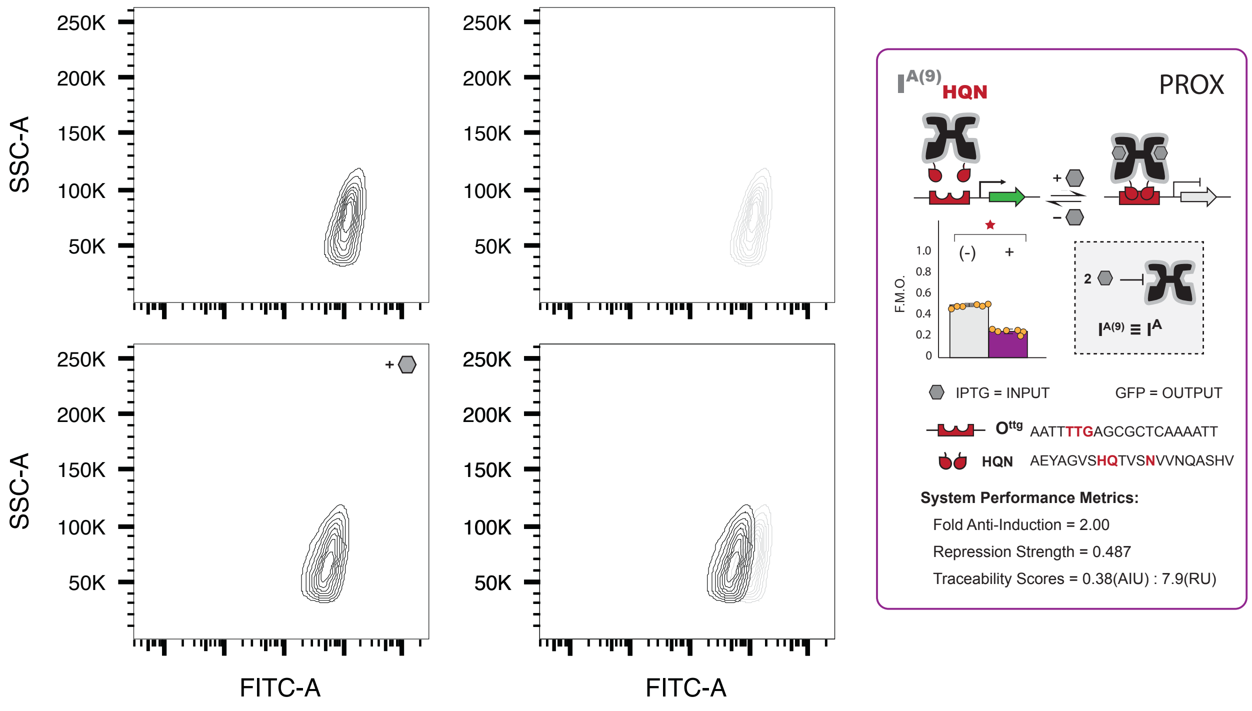

SSC-A
FITC-A
250K
200K
150K
100K
50K
PROX
HQN
IPTG = INPUT
GFP = OUTPUT
F.M.O.
(-)
+
AATTTTGAGCGCTCAAAATT
AEYAGVSHQTVSNVVNQASHV
System Performance Metrics:
Fold Anti-Induction = 2.00
Repression Strength = 0.487
Traceability Scores = 0.38(AIU) : 7.9(RU)

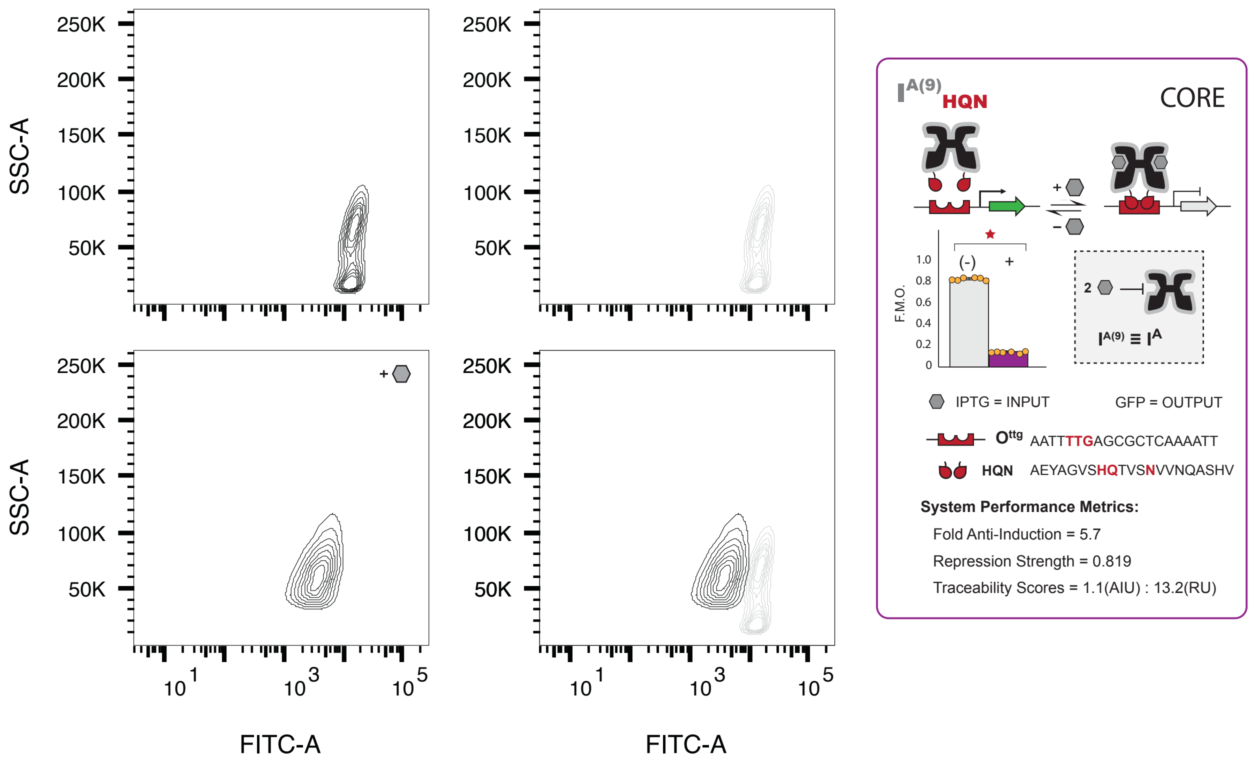

SSC-A
FITC-A
250K
200K
150K
100K
50K
10 1
10 3
10 5
CORE
HQN
IPTG = INPUT
GFP = OUTPUT
F.M.O.
(-)
+
AATTTTGAGCGCTCAAAATT
AEYAGVSHQTVSNVVNQASHV
System Performance Metrics:
Fold Anti-Induction = 5.7
Repression Strength = 0.819
Traceability Scores = 1.1(AIU) : 13.2(RU)

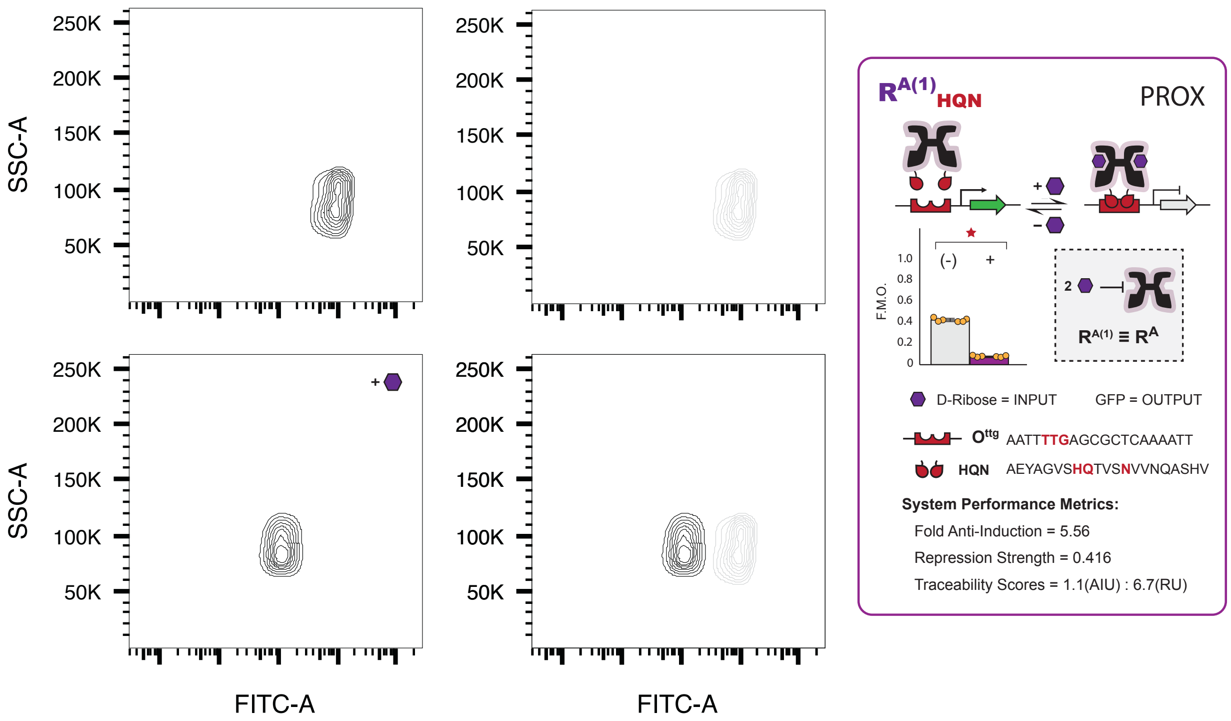

SSC-A
250K
200K
150K
100K
50K
FITC-A
R^A(1) HQN
PROX
1.0
0.8
0.6
0.4
0.2
0
F.M.O.
(-)
+
2
R^A(1) ≡ R^A
D-Ribose = INPUT
GFP = OUTPUT
O^ttg AATTTTGAGCGCTCAAAATT
HQN AEYAGVSHQTVSNVVNQASHV
System Performance Metrics:
Fold Anti-Induction = 5.56
Repression Strength = 0.416
Traceability Scores = 1.1(AIU) : 6.7(RU)

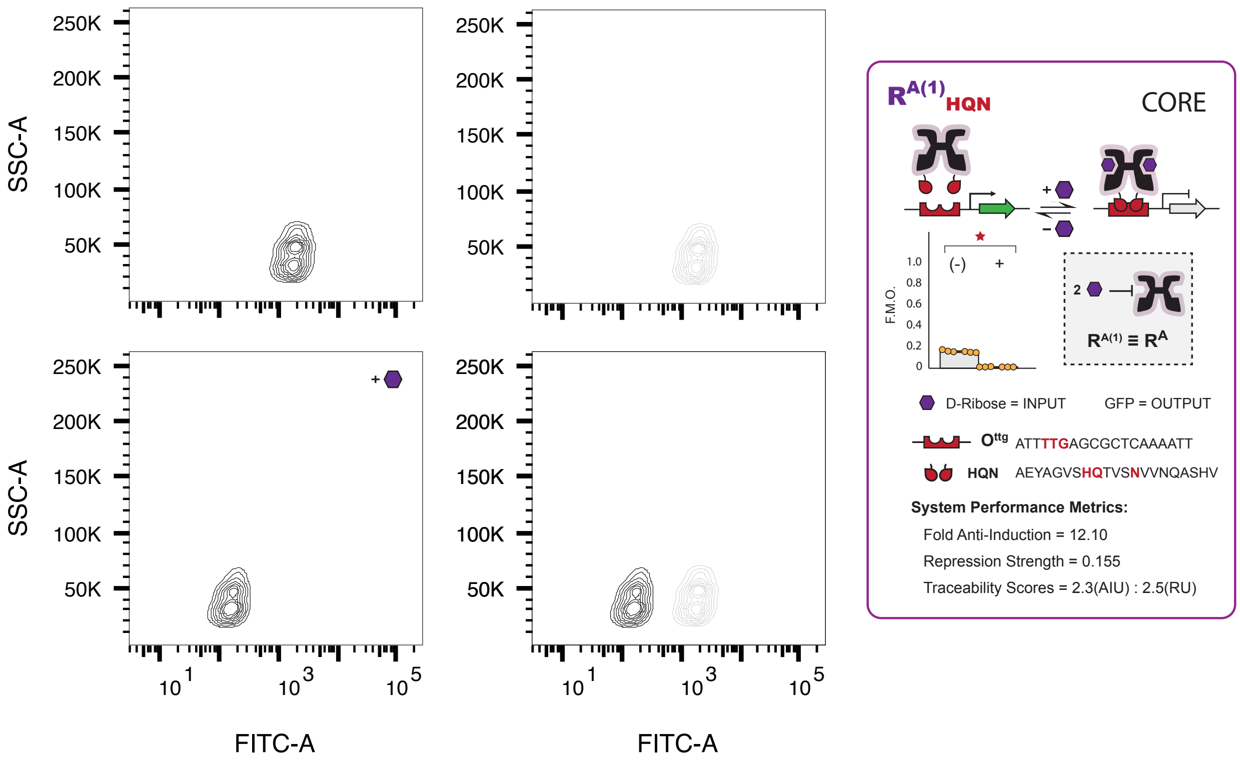

SSC-A
250K
200K
150K
100K
50K
10^1
10^3
10^5
FITC-A
R^A(1) HQN
CORE
1.0
0.8
0.6
0.4
0.2
0
F.M.O.
(-)
+
2
R^A(1) ≡ R^A
D-Ribose = INPUT
GFP = OUTPUT
O^ttg ATTTTGAGCGCTCAAAATT
HQN AEYAGVSHQTVSNVVNQASHV
System Performance Metrics:
Fold Anti-Induction = 12.10
Repression Strength = 0.155
Traceability Scores = 2.3(AIU) : 2.5(RU)

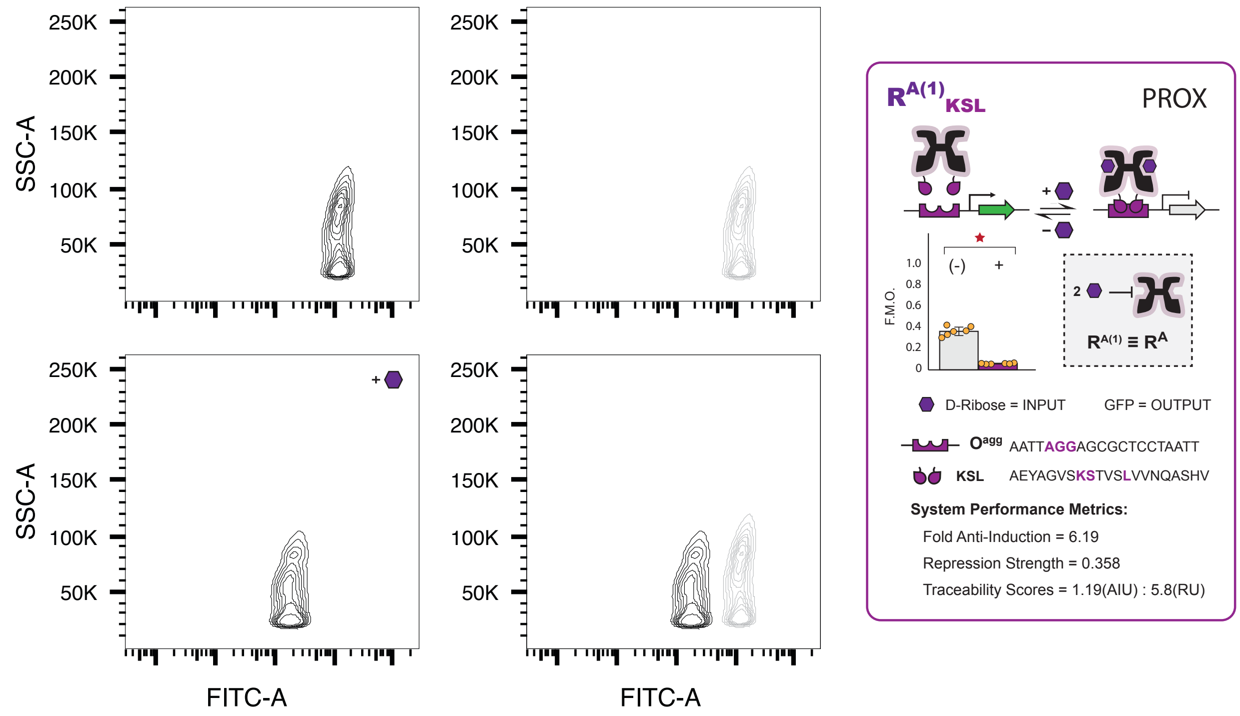

SSC-A
250K
200K
150K
100K
50K
FITC-A
+
R^A(1)_KSL
PROX
+
−
(-)
+
F.M.O.
1.0
0.8
0.6
0.4
0.2
0
2
R^A(1) ≡ R^A
D-Ribose = INPUT
GFP = OUTPUT
O^agg AATTAGGAGCGCTCCTAATT
KSL AEYAGVSKSTVSLVVNQASHV
System Performance Metrics:
Fold Anti-Induction = 6.19
Repression Strength = 0.358
Traceability Scores = 1.19(AIU) : 5.8(RU)

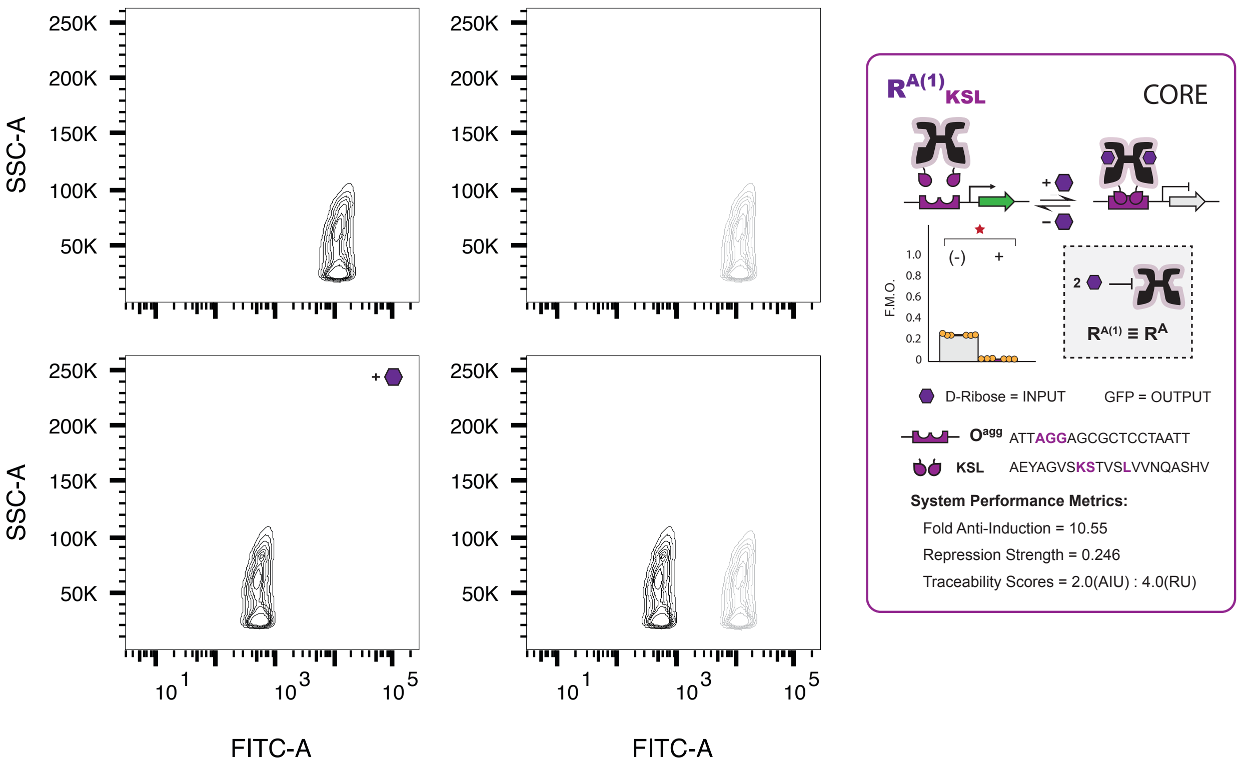

SSC-A
250K
200K
150K
100K
50K
10^1
10^3
10^5
FITC-A
+
R^A(1)_KSL
CORE
+
−
(-)
+
F.M.O.
1.0
0.8
0.6
0.4
0.2
0
2
R^A(1) ≡ R^A
D-Ribose = INPUT
GFP = OUTPUT
O^agg ATTAGGAGCGCTCCTAATT
KSL AEYAGVSKSTVSLVVNQASHV
System Performance Metrics:
Fold Anti-Induction = 10.55
Repression Strength = 0.246
Traceability Scores = 2.0(AIU) : 4.0(RU)

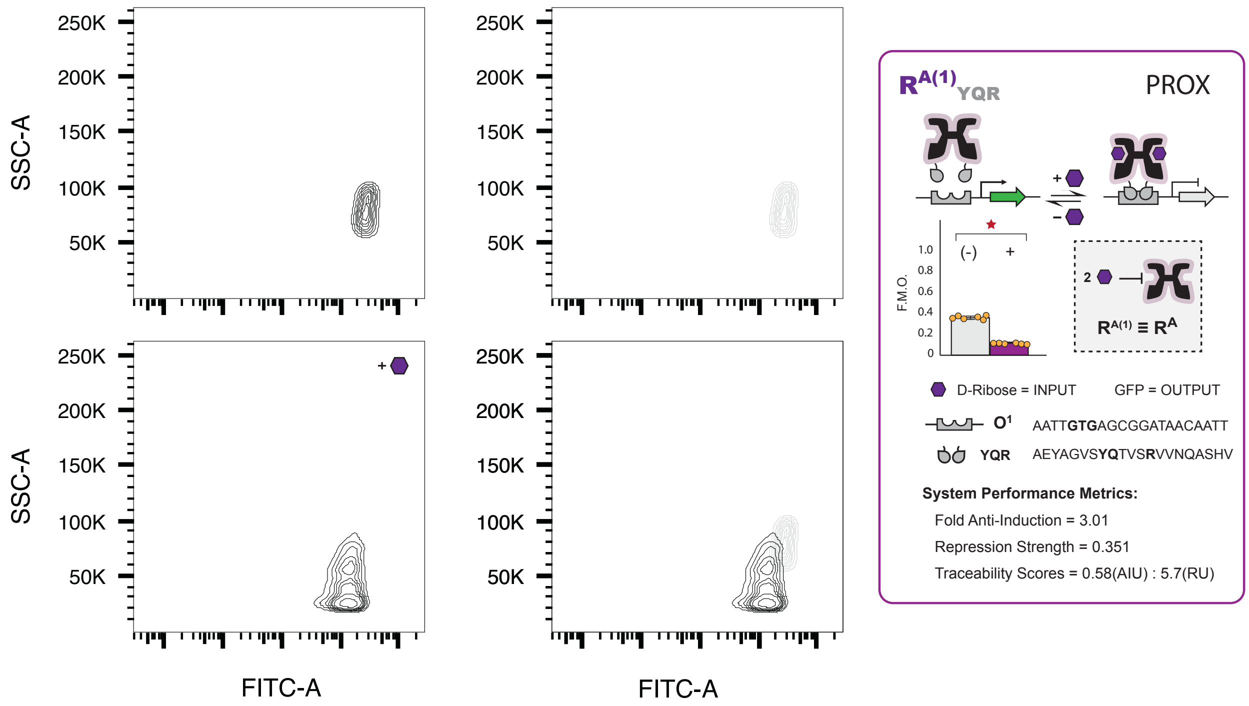
SSC-A
250K
200K
150K
100K
50K
FITC-A
+
R^A(1) YQR
PROX
1.0
0.8
0.6
0.4
0.2
0
F.M.O.
(-)
+
2
R^A(1) ≡ R^A
D-Ribose = INPUT
GFP = OUTPUT
O1
AATTGTGAGCGGATAACAATT
YQR
AEYAGVSYQTVSRVVNQASHV
System Performance Metrics:
Fold Anti-Induction = 3.01
Repression Strength = 0.351
Traceability Scores = 0.58(AIU) : 5.7(RU)

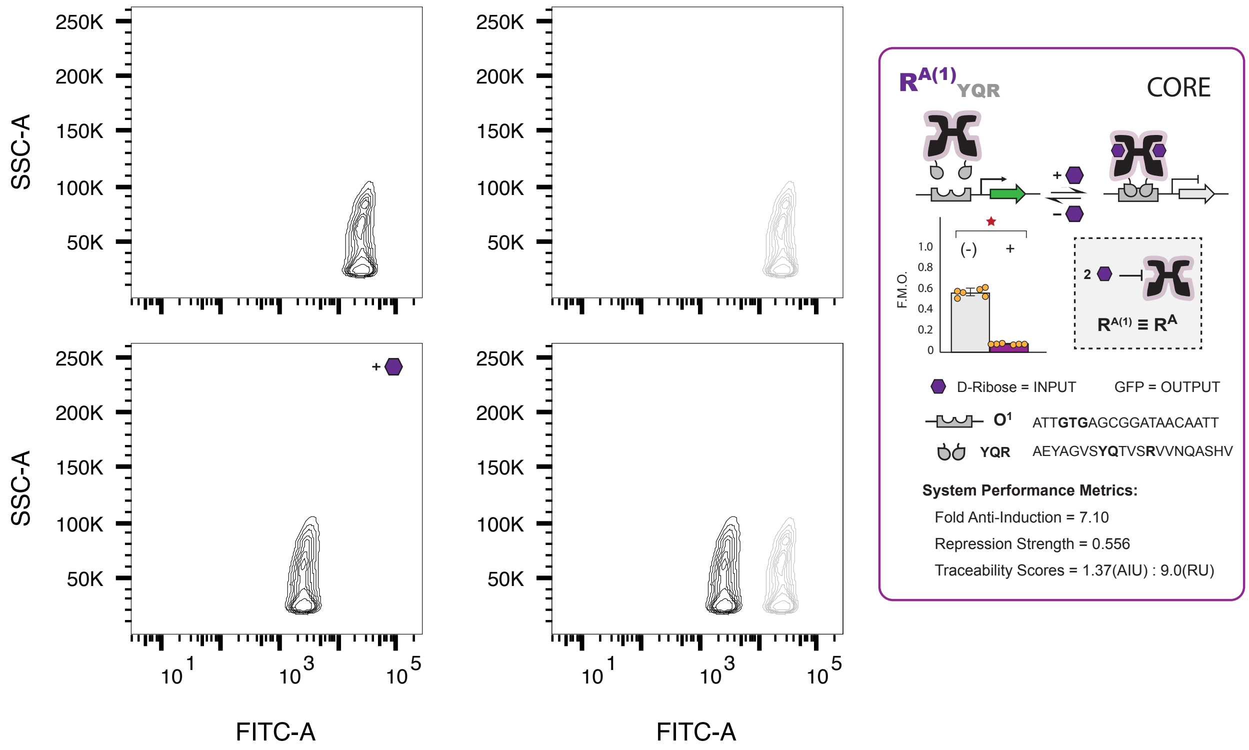
SSC-A
250K
200K
150K
100K
50K
10 1
10 3
10 5
FITC-A
+
R^A(1) YQR
CORE
1.0
0.8
0.6
0.4
0.2
0
F.M.O.
(-)
+
2
R^A(1) ≡ R^A
D-Ribose = INPUT
GFP = OUTPUT
O1
ATTGTGAGCGGATAACAATT
YQR
AEYAGVSYQTVSRVVNQASHV
System Performance Metrics:
Fold Anti-Induction = 7.10
Repression Strength = 0.556
Traceability Scores = 1.37(AIU) : 9.0(RU)

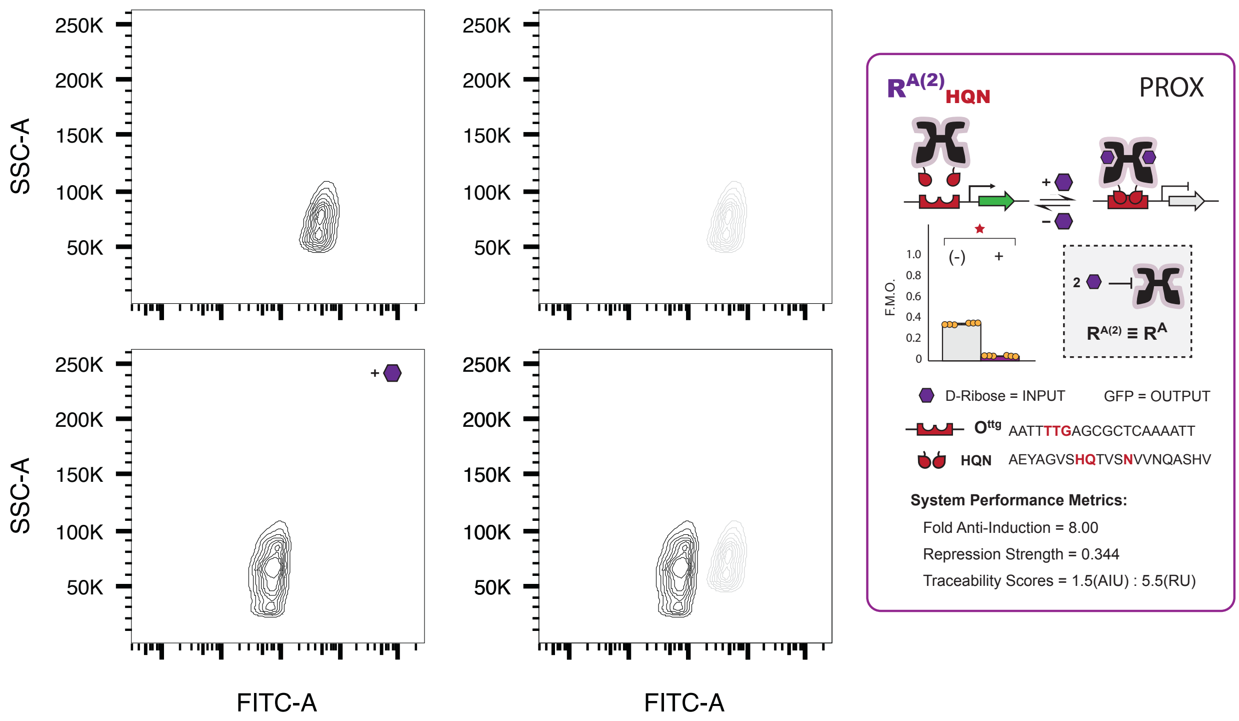
SSC-A
250K
200K
150K
100K
50K
FITC-A
R^A(2) HQN
PROX
F.M.O.
1.0
0.8
0.6
0.4
0.2
0
(-)
+
2
R^A(2) ≡ R^A
D-Ribose = INPUT
GFP = OUTPUT
O^ttg AATTTTGAGCGCTCAAAATT
HQN AEYAGVSHQTVSNVVNQASHV
System Performance Metrics:
Fold Anti-Induction = 8.00
Repression Strength = 0.344
Traceability Scores = 1.5(AIU) : 5.5(RU)

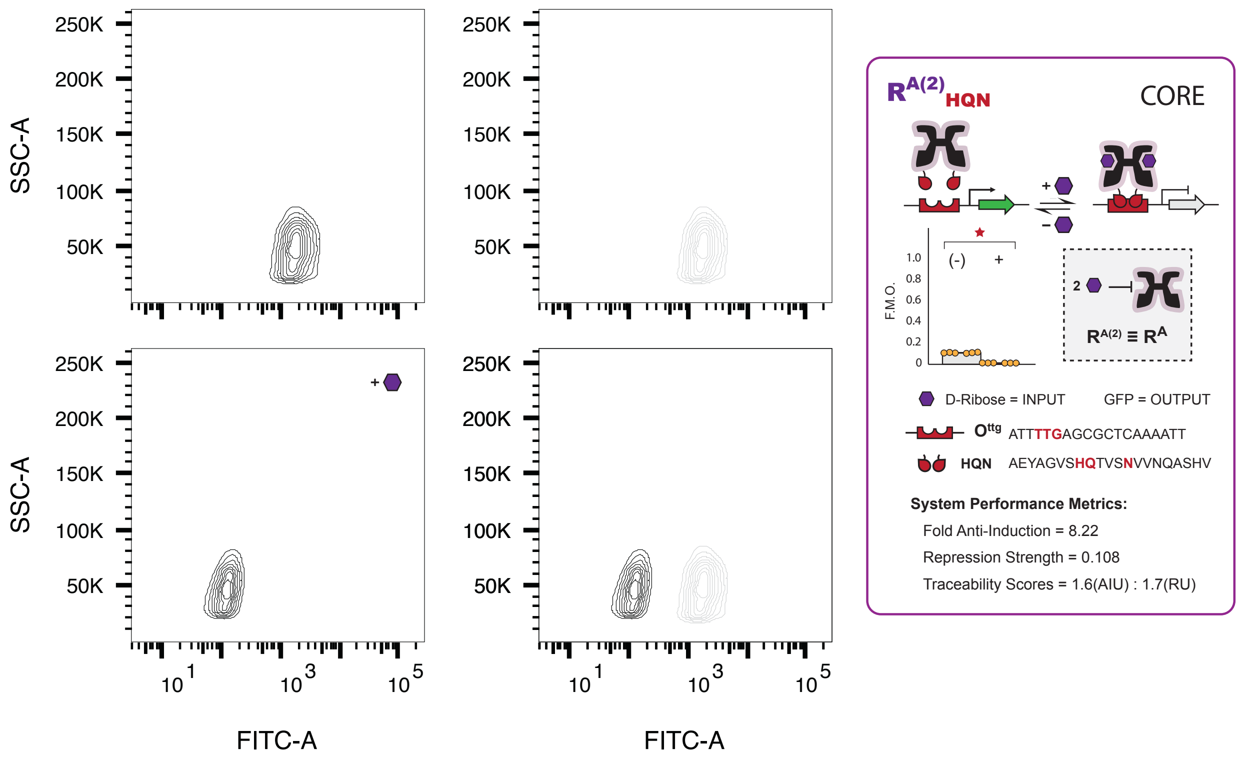
SSC-A
250K
200K
150K
100K
50K
10^1
10^3
10^5
FITC-A
R^A(2) HQN
CORE
F.M.O.
1.0
0.8
0.6
0.4
0.2
0
(-)
+
2
R^A(2) ≡ R^A
D-Ribose = INPUT
GFP = OUTPUT
O^ttg ATTTTGAGCGCTCAAAATT
HQN AEYAGVSHQTVSNVVNQASHV
System Performance Metrics:
Fold Anti-Induction = 8.22
Repression Strength = 0.108
Traceability Scores = 1.6(AIU) : 1.7(RU)

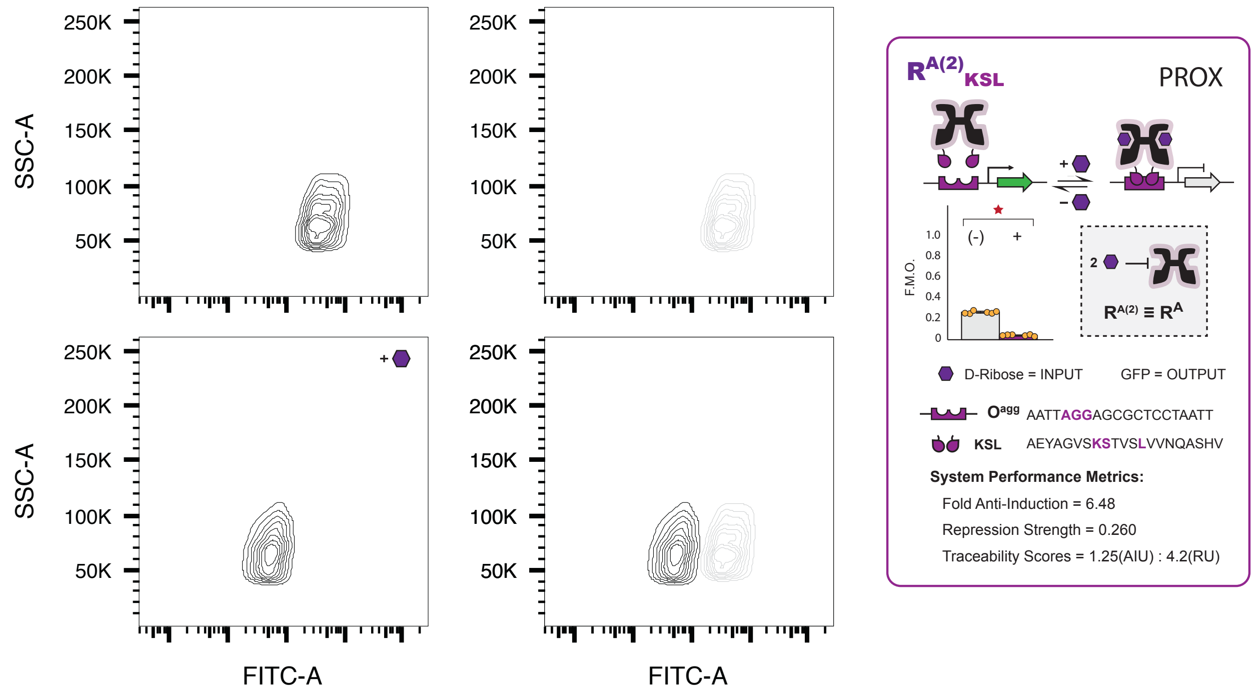

SSC-A
250K
200K
150K
100K
50K
FITC-A
+
$R^{A(2)}{}_{KSL}$
PROX
+
–
F.M.O.
1.0
0.8
0.6
0.4
0.2
0
(-)
+
2
$R^{A(2)} \equiv R^{A}$
D-Ribose = INPUT
GFP = OUTPUT
O^{agg} AATTAGGAGCGCTCCTAATT
KSL AEYAGVSKSTVSLVVNQASHV
System Performance Metrics:
Fold Anti-Induction = 6.48
Repression Strength = 0.260
Traceability Scores = 1.25(AIU) : 4.2(RU)

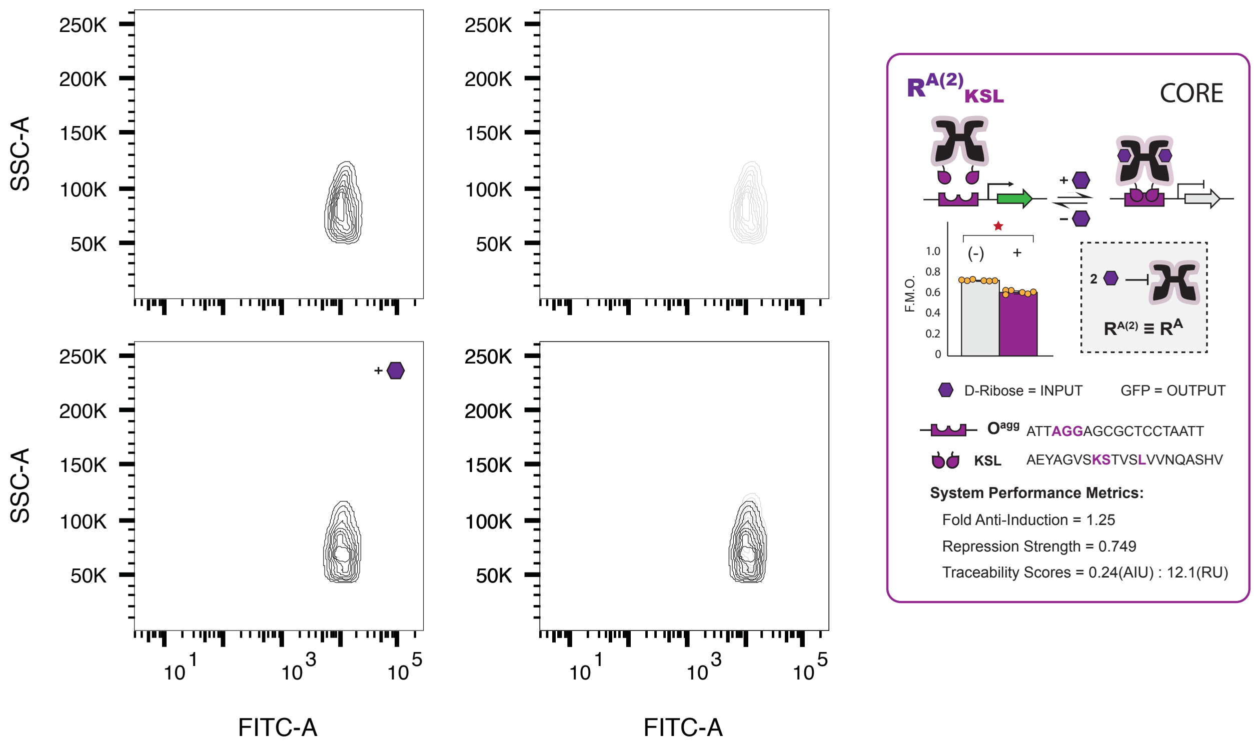

SSC-A
250K
200K
150K
100K
50K
+
10^1
10^3
10^5
FITC-A
$R^{A(2)}{}_{KSL}$
CORE
+
–
F.M.O.
1.0
0.8
0.6
0.4
0.2
0
(-)
+
2
$R^{A(2)} \equiv R^{A}$
D-Ribose = INPUT
GFP = OUTPUT
O^{agg} ATTAGGAGCGCTCCTAATT
KSL AEYAGVSKSTVSLVVNQASHV
System Performance Metrics:
Fold Anti-Induction = 1.25
Repression Strength = 0.749
Traceability Scores = 0.24(AIU) : 12.1(RU)

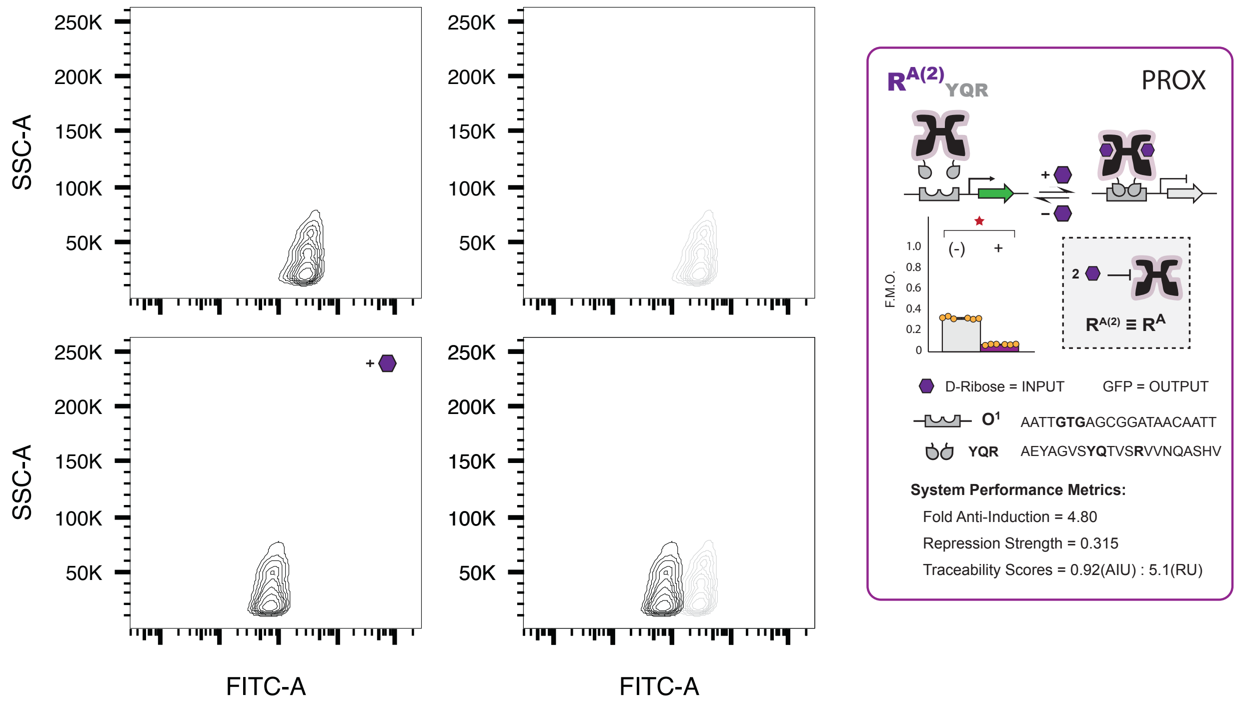
SSC-A
250K
200K
150K
100K
50K
FITC-A
R^A(2) YQR
PROX
F.M.O.
(-)
+
2
R^A(2) ≡ R^A
D-Ribose = INPUT
GFP = OUTPUT
O1
AATTGTGAGCGGATAACAATT
YQR
AEYAGVSYQTVSRVVNQASHV
System Performance Metrics:
Fold Anti-Induction = 4.80
Repression Strength = 0.315
Traceability Scores = 0.92(AIU) : 5.1(RU)

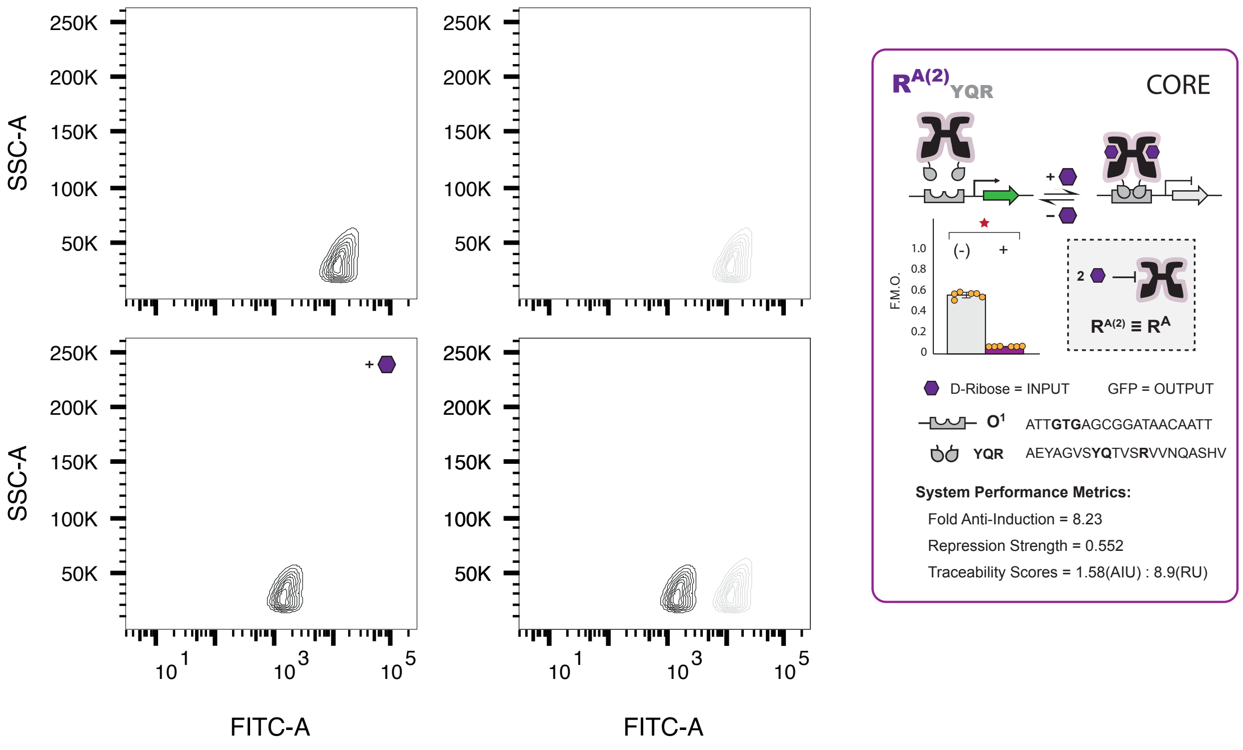
SSC-A
250K
200K
150K
100K
50K
10 1
10 3
10 5
FITC-A
R^A(2) YQR
CORE
F.M.O.
(-)
+
2
R^A(2) ≡ R^A
D-Ribose = INPUT
GFP = OUTPUT
O1
ATTGTGAGCGGATAACAATT
YQR
AEYAGVSYQTVSRVVNQASHV
System Performance Metrics:
Fold Anti-Induction = 8.23
Repression Strength = 0.552
Traceability Scores = 1.58(AIU) : 8.9(RU)

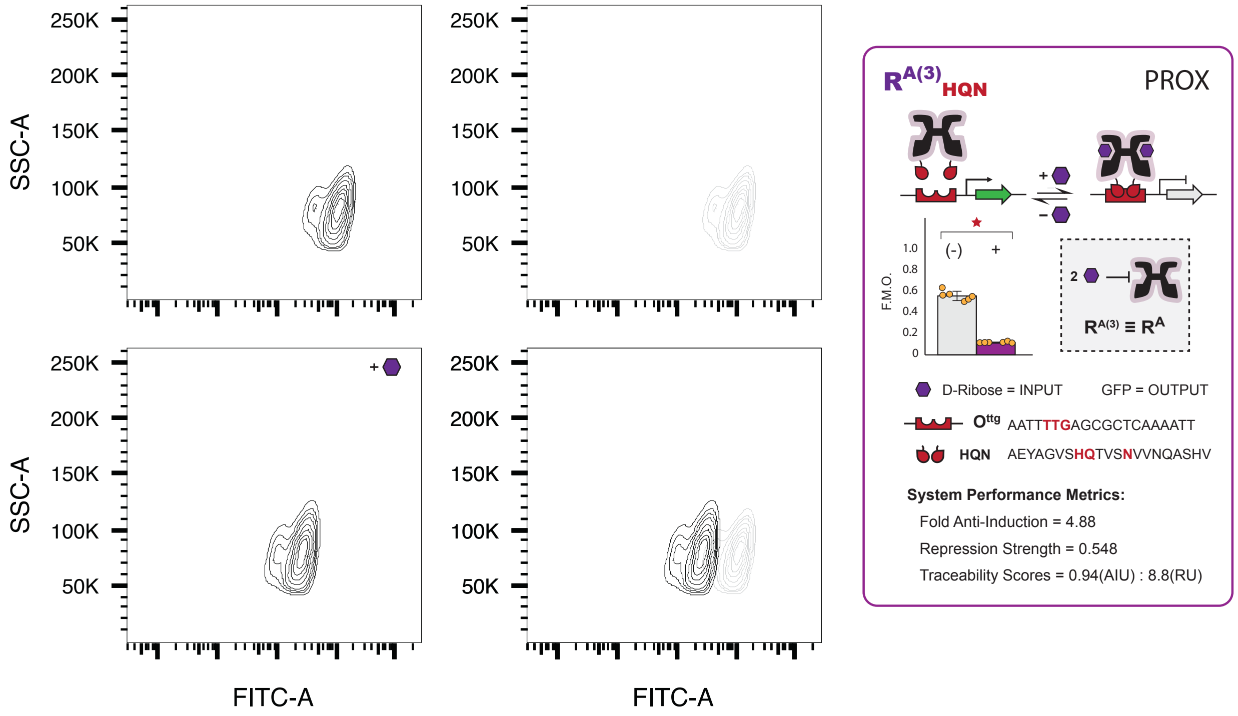
SSC-A
250K
200K
150K
100K
50K
FITC-A
+
R^A(3) HQN
PROX
+
−
1.0
0.8
0.6
0.4
0.2
0
F.M.O.
(-)
+
2
R^A(3) ≡ R^A
D-Ribose = INPUT
GFP = OUTPUT
O^ttg AATTTTGAGCGCTCAAAATT
HQN AEYAGVSHQTVSNVVNQASHV
System Performance Metrics:
Fold Anti-Induction = 4.88
Repression Strength = 0.548
Traceability Scores = 0.94(AIU) : 8.8(RU)

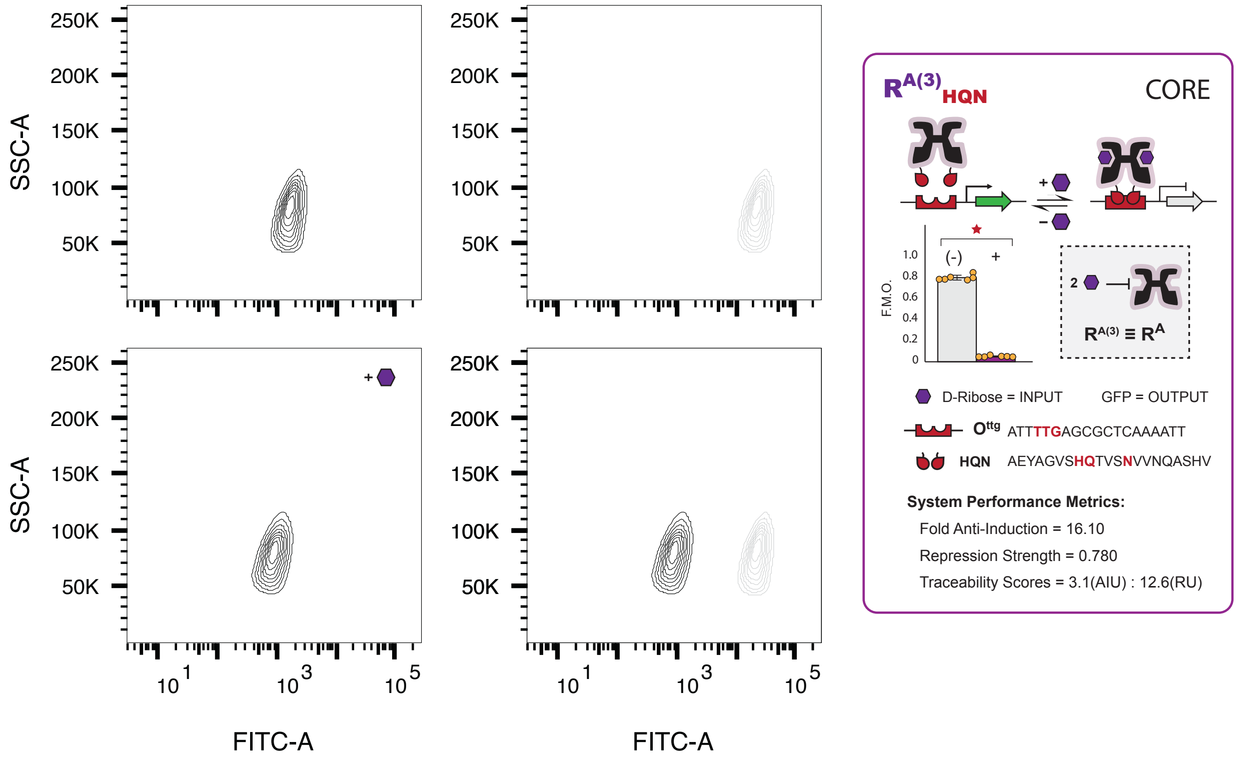
SSC-A
250K
200K
150K
100K
50K
10^1
10^3
10^5
FITC-A
+
R^A(3) HQN
CORE
+
−
1.0
0.8
0.6
0.4
0.2
0
F.M.O.
(-)
+
2
R^A(3) ≡ R^A
D-Ribose = INPUT
GFP = OUTPUT
O^ttg ATTTTGAGCGCTCAAAATT
HQN AEYAGVSHQTVSNVVNQASHV
System Performance Metrics:
Fold Anti-Induction = 16.10
Repression Strength = 0.780
Traceability Scores = 3.1(AIU) : 12.6(RU)

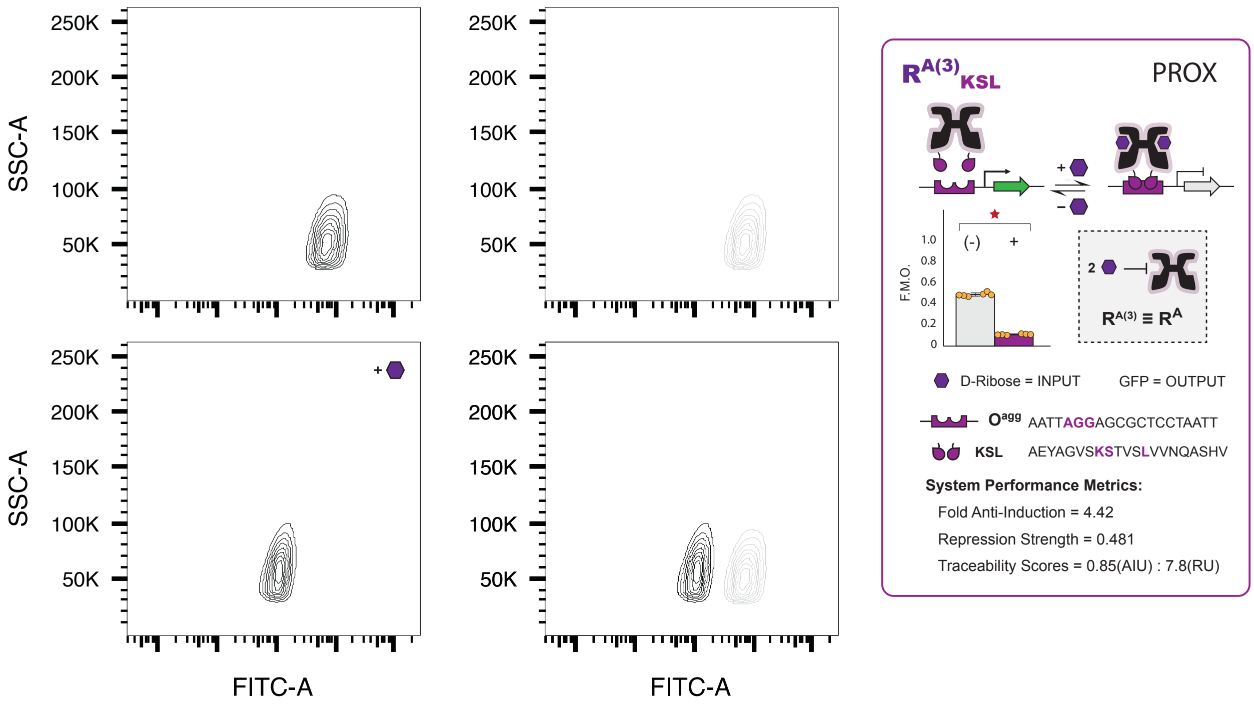
250K
200K
150K
100K
50K
SSC-A
FITC-A
$R^{A(3)}_{KSL}$
PROX
F.M.O.
1.0
0.8
0.6
0.4
0.2
0
(-)
+
2
$R^{A(3)} \equiv R^{A}$
D-Ribose = INPUT
GFP = OUTPUT
O^{agg} AATTAGGAGCGCTCCTAATT
KSL AEYAGVSKSTVSLVVNQASHV
System Performance Metrics:
Fold Anti-Induction = 4.42
Repression Strength = 0.481
Traceability Scores = 0.85(AIU) : 7.8(RU)

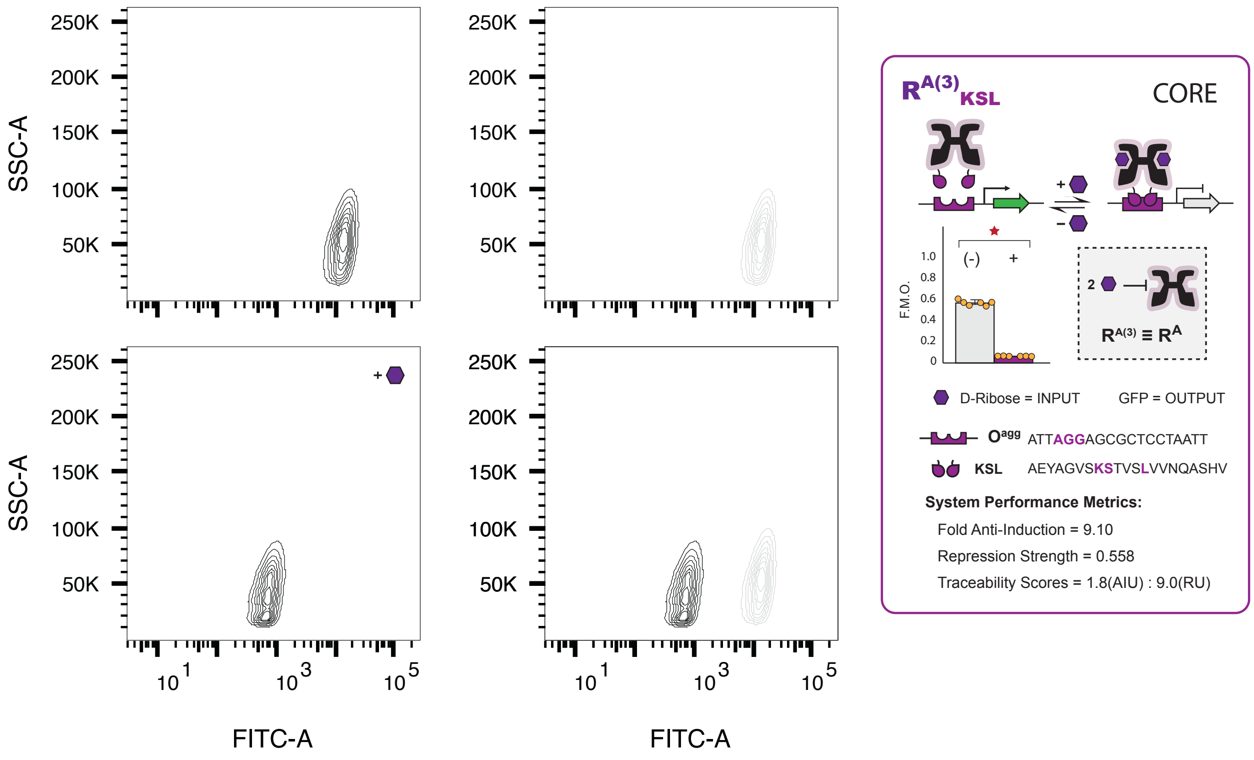
250K
200K
150K
100K
50K
SSC-A
10^1
10^3
10^5
FITC-A
$R^{A(3)}_{KSL}$
CORE
F.M.O.
1.0
0.8
0.6
0.4
0.2
0
(-)
+
2
$R^{A(3)} \equiv R^{A}$
D-Ribose = INPUT
GFP = OUTPUT
O^{agg} ATTAGGAGCGCTCCTAATT
KSL AEYAGVSKSTVSLVVNQASHV
System Performance Metrics:
Fold Anti-Induction = 9.10
Repression Strength = 0.558
Traceability Scores = 1.8(AIU) : 9.0(RU)

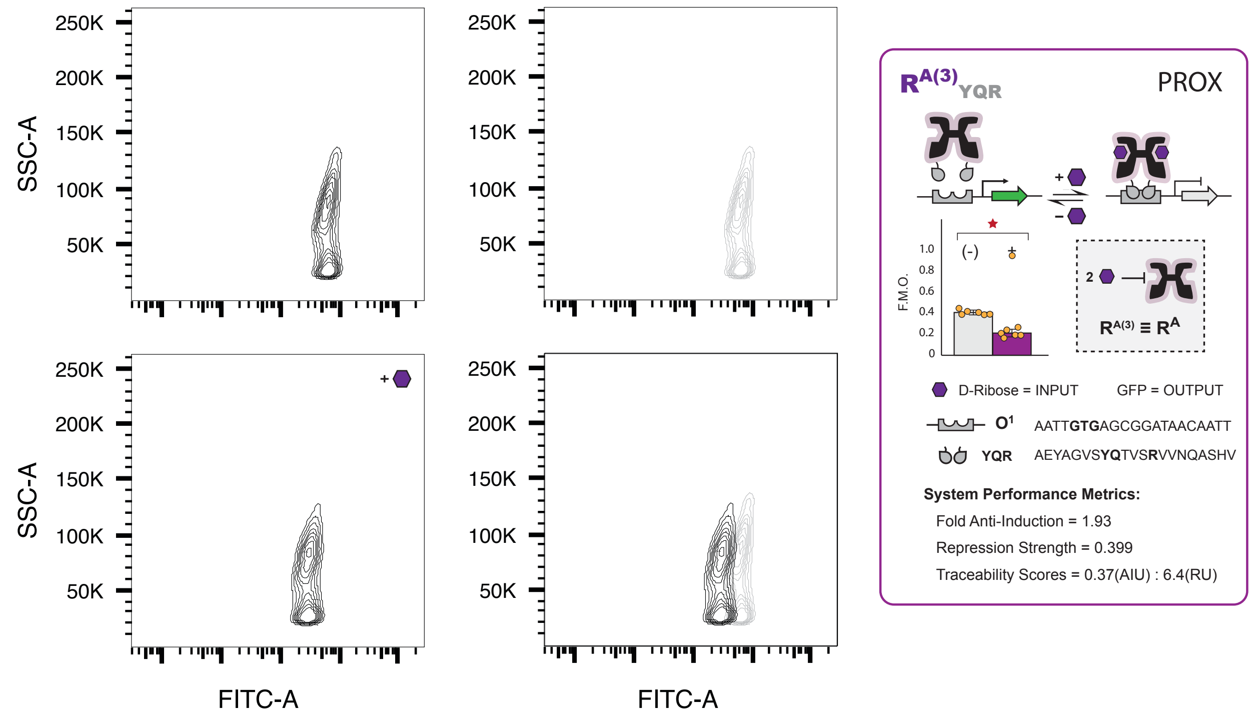

SSC-A
FITC-A
250K
200K
150K
100K
50K
$R^{A(3)}_{YQR}$
PROX
F.M.O.
(-)
+
$R^{A(3)} \equiv R^{A}$
D-Ribose = INPUT
GFP = OUTPUT
O1
AATTGTGAGCGGATAACAATT
YQR
AEYAGVSYQTVSRVVNQASHV
System Performance Metrics:
Fold Anti-Induction = 1.93
Repression Strength = 0.399
Traceability Scores = 0.37(AIU) : 6.4(RU)

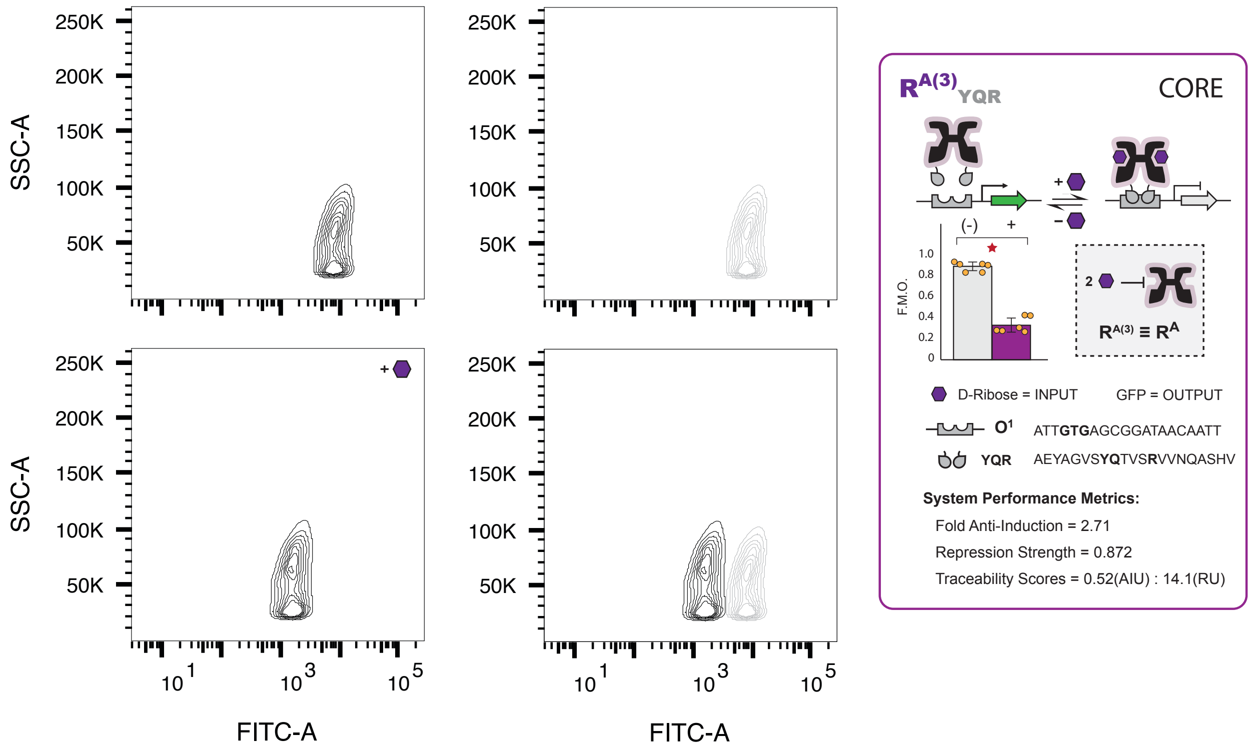

SSC-A
FITC-A
250K
200K
150K
100K
50K
10^1
10^3
10^5
$R^{A(3)}_{YQR}$
CORE
F.M.O.
(-)
+
$R^{A(3)} \equiv R^{A}$
D-Ribose = INPUT
GFP = OUTPUT
O1
ATTGTGAGCGGATAACAATT
YQR
AEYAGVSYQTVSRVVNQASHV
System Performance Metrics:
Fold Anti-Induction = 2.71
Repression Strength = 0.872
Traceability Scores = 0.52(AIU) : 14.1(RU)

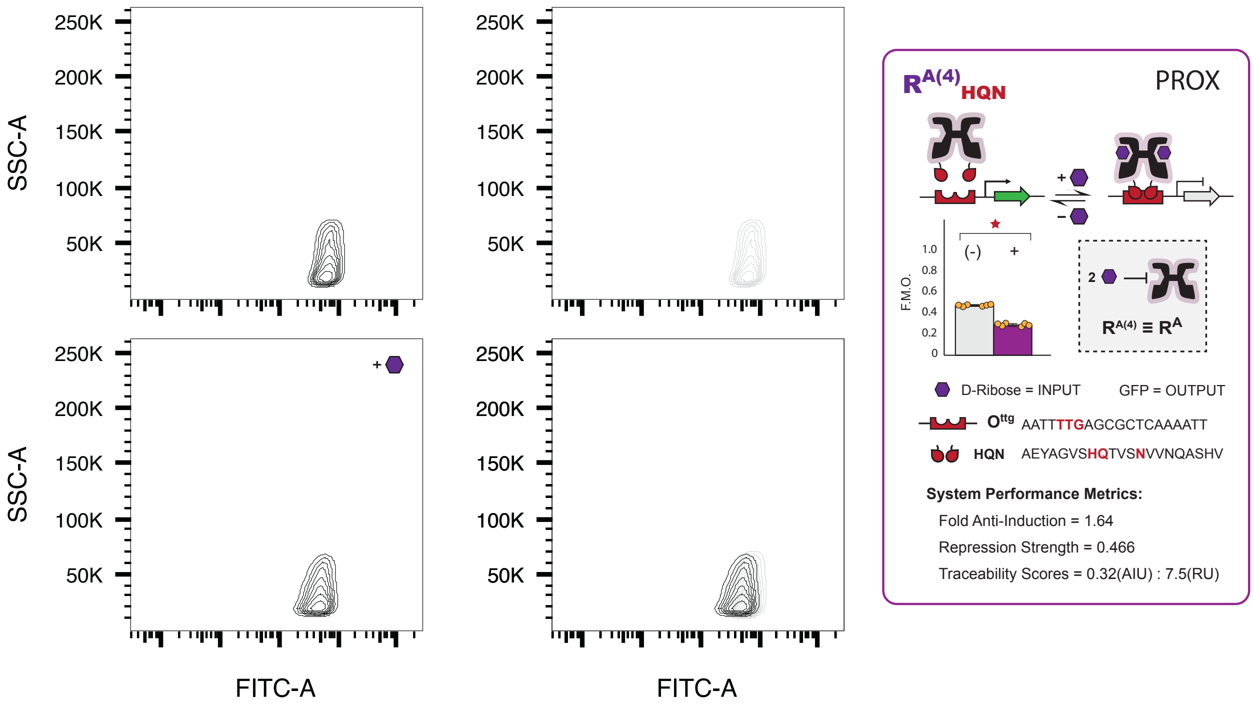
SSC-A
250K
200K
150K
100K
50K
FITC-A
R^A(4) HQN
PROX
1.0
0.8
0.6
0.4
0.2
0
F.M.O.
(-)
+
2
R^A(4) ≡ R^A
D-Ribose = INPUT
GFP = OUTPUT
O^ttg AATTTTGAGCGCTCAAAATT
HQN AEYAGVSHQTVSNVVNQASHV
System Performance Metrics:
Fold Anti-Induction = 1.64
Repression Strength = 0.466
Traceability Scores = 0.32(AIU) : 7.5(RU)

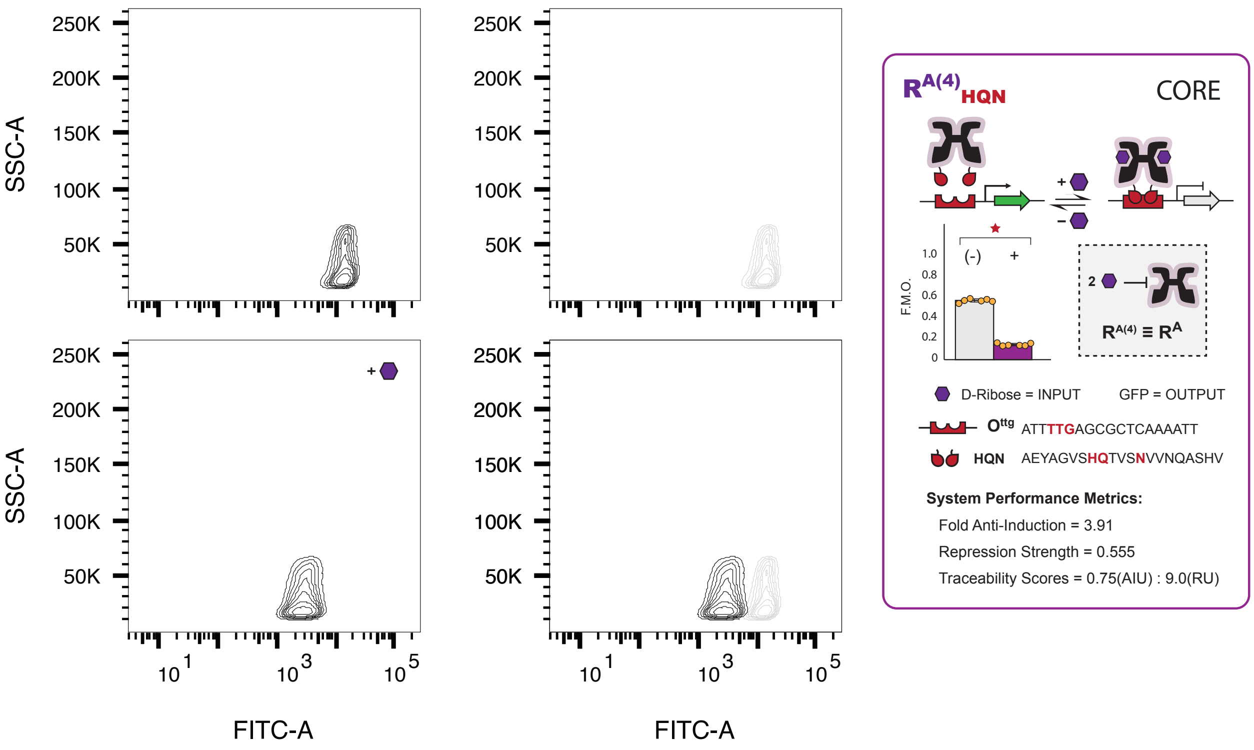
SSC-A
250K
200K
150K
100K
50K
10^1
10^3
10^5
FITC-A
R^A(4) HQN
CORE
1.0
0.8
0.6
0.4
0.2
0
F.M.O.
(-)
+
2
R^A(4) ≡ R^A
D-Ribose = INPUT
GFP = OUTPUT
O^ttg ATTTTGAGCGCTCAAAATT
HQN AEYAGVSHQTVSNVVNQASHV
System Performance Metrics:
Fold Anti-Induction = 3.91
Repression Strength = 0.555
Traceability Scores = 0.75(AIU) : 9.0(RU)

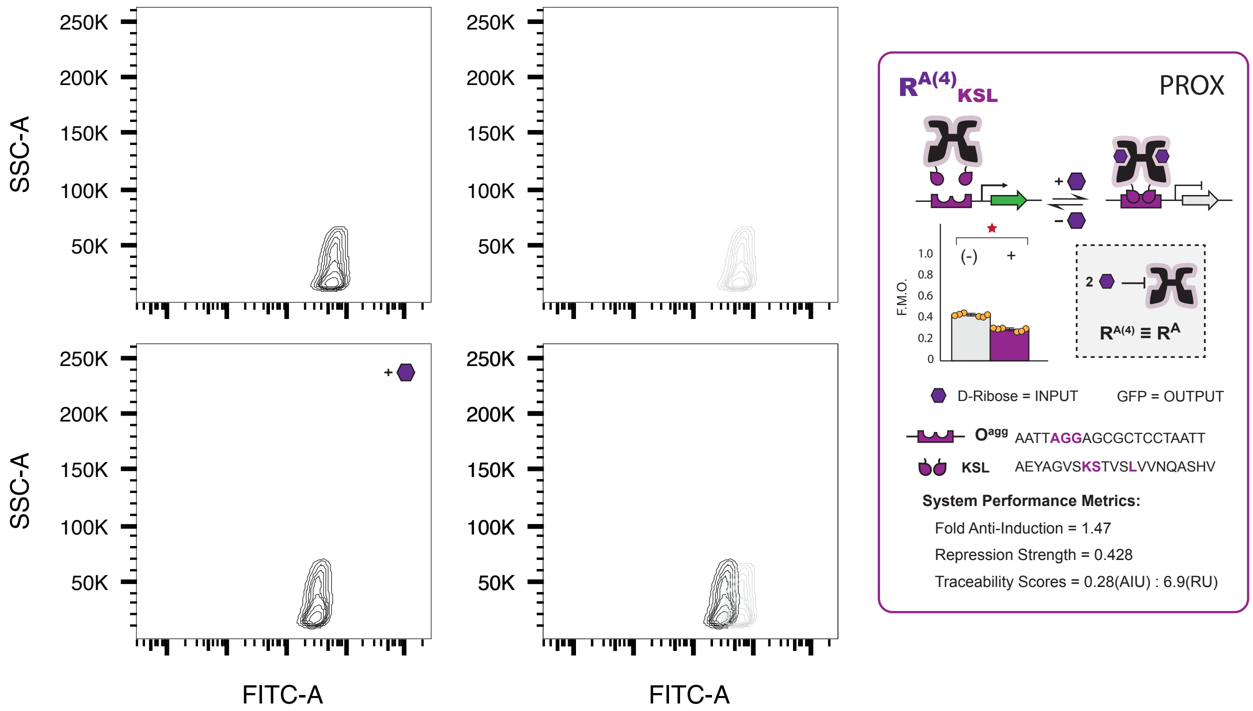

SSC-A
250K
200K
150K
100K
50K
FITC-A
RA(4)KSL
PROX
(-)
+
F.M.O.
1.0
0.8
0.6
0.4
0.2
0
2
RA(4) ≡ RA
D-Ribose = INPUT
GFP = OUTPUT
Oagg AATTAGGAGCGCTCCTAATT
KSL AEYAGVSKSTVSLVVNQASHV
System Performance Metrics:
Fold Anti-Induction = 1.47
Repression Strength = 0.428
Traceability Scores = 0.28(AIU) : 6.9(RU)

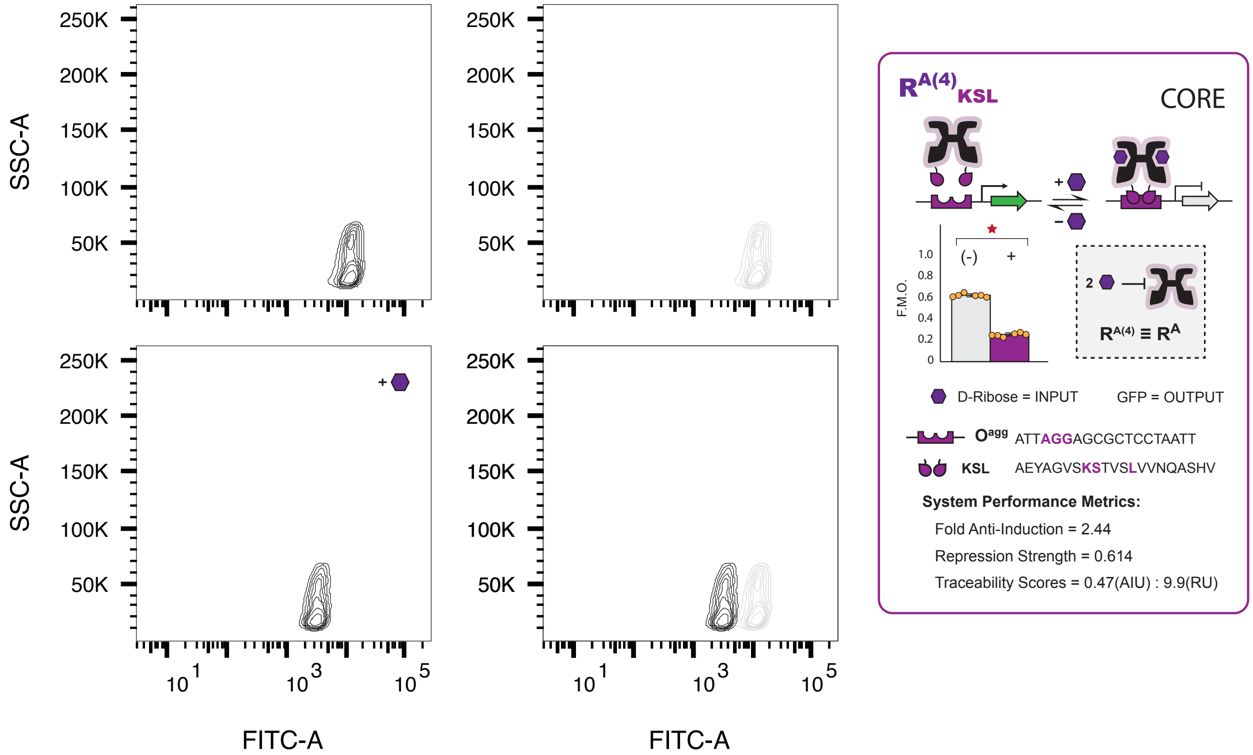

SSC-A
250K
200K
150K
100K
50K
10 1
10 3
10 5
FITC-A
RA(4)KSL
CORE
(-)
+
F.M.O.
1.0
0.8
0.6
0.4
0.2
0
2
RA(4) ≡ RA
D-Ribose = INPUT
GFP = OUTPUT
Oagg ATTAGGAGCGCTCCTAATT
KSL AEYAGVSKSTVSLVVNQASHV
System Performance Metrics:
Fold Anti-Induction = 2.44
Repression Strength = 0.614
Traceability Scores = 0.47(AIU) : 9.9(RU)

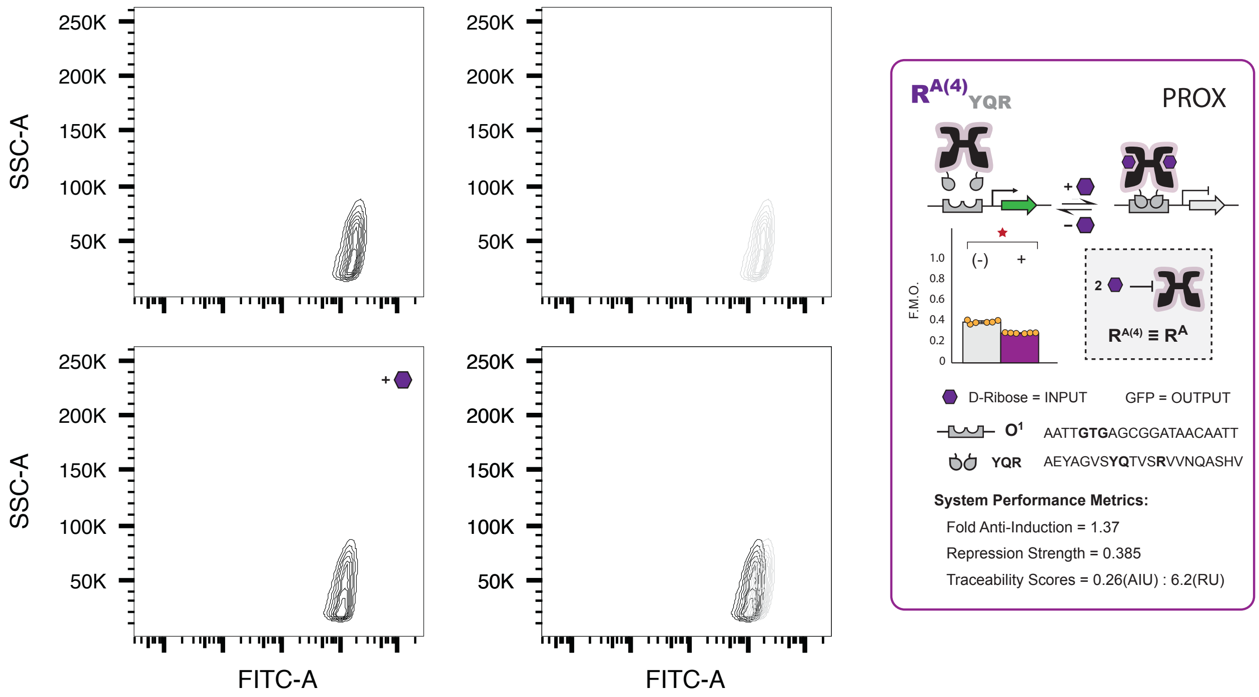

SSC-A
250K
200K
150K
100K
50K
FITC-A
R^A(4)_YQR
PROX
F.M.O.
1.0
0.8
0.6
0.4
0.2
0
(-)
+
2
R^A(4) ≡ R^A
D-Ribose = INPUT
GFP = OUTPUT
O1
AATTGTGAGCGGATAACAATT
YQR
AEYAGVSYQTVSRVVNQASHV
System Performance Metrics:
Fold Anti-Induction = 1.37
Repression Strength = 0.385
Traceability Scores = 0.26(AIU) : 6.2(RU)

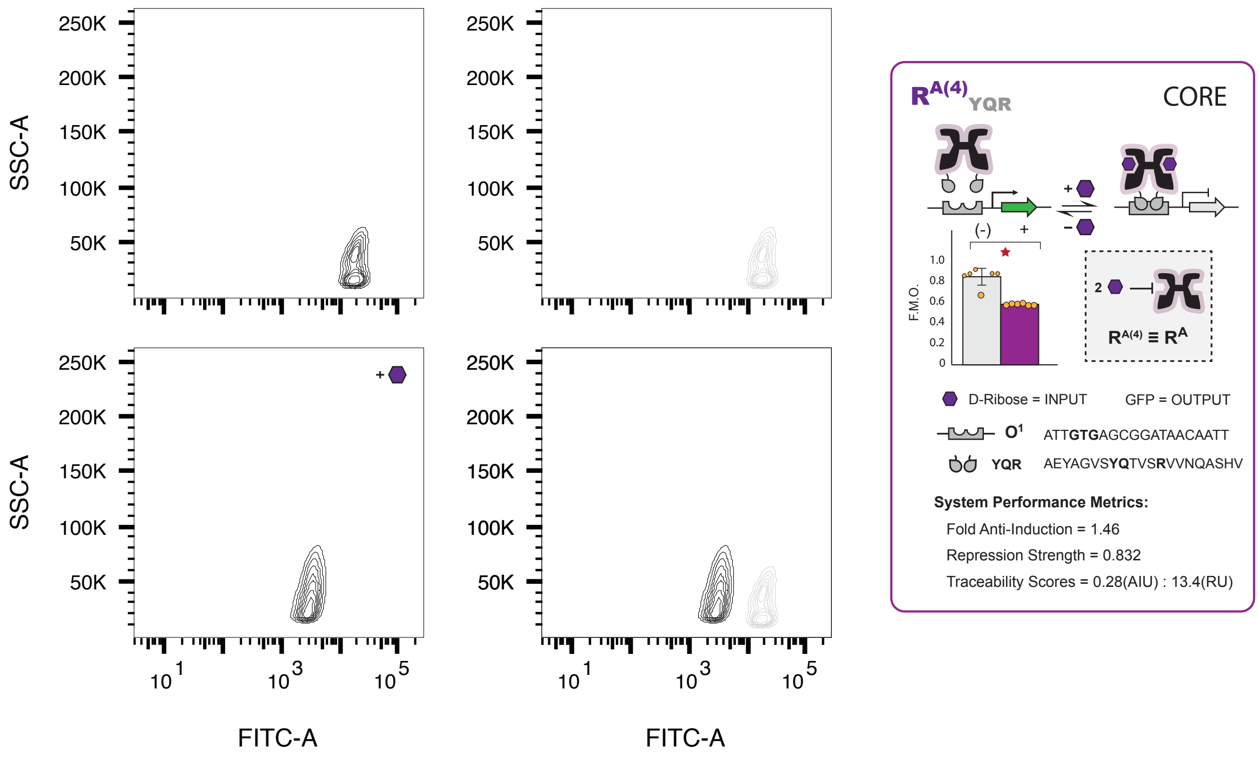

SSC-A
250K
200K
150K
100K
50K
10 1
10 3
10 5
FITC-A
R^A(4)_YQR
CORE
F.M.O.
1.0
0.8
0.6
0.4
0.2
0
(-)
+
2
R^A(4) ≡ R^A
D-Ribose = INPUT
GFP = OUTPUT
O1
ATTGTGAGCGGATAACAATT
YQR
AEYAGVSYQTVSRVVNQASHV
System Performance Metrics:
Fold Anti-Induction = 1.46
Repression Strength = 0.832
Traceability Scores = 0.28(AIU) : 13.4(RU)

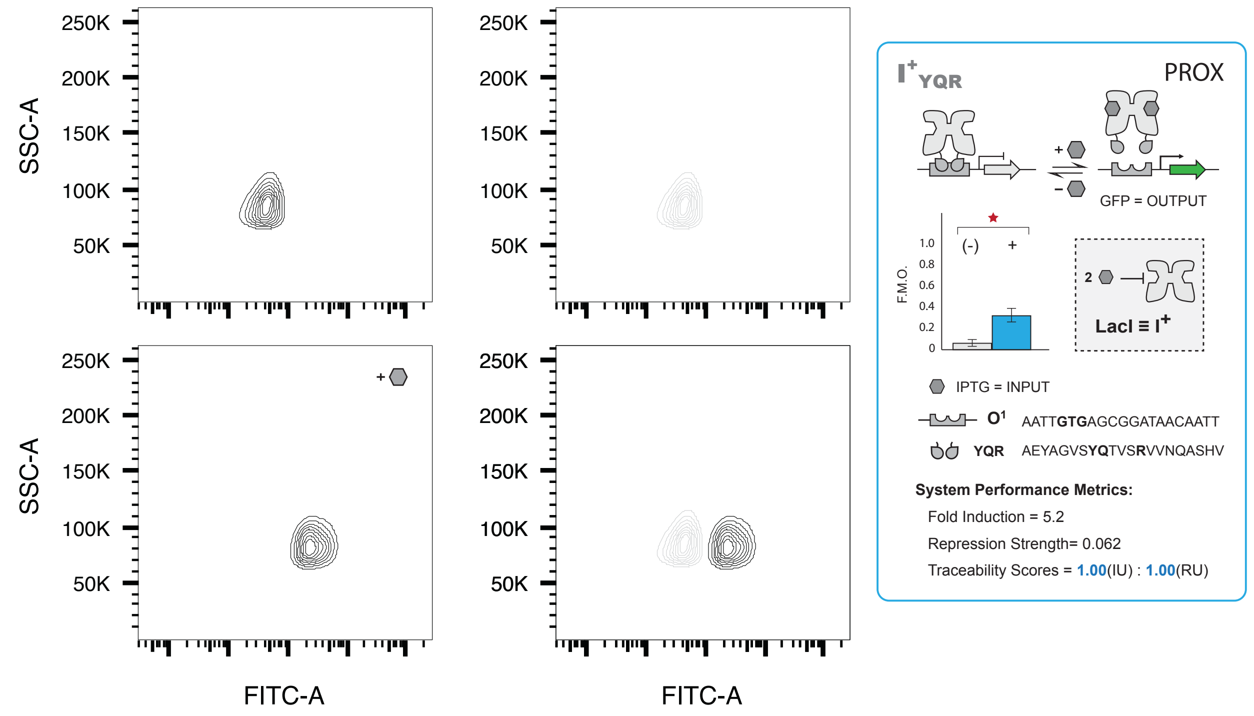
SSC-A
250K
200K
150K
100K
50K
FITC-A
FITC-A
I+YQR
PROX
GFP = OUTPUT
F.M.O.
(-)
+
2
LacI ≡ I+
IPTG = INPUT
O1 AATTGTGAGCGGATAACAATT
YQR AEYAGVSYQTVSRVVNQASHV
System Performance Metrics:
Fold Induction = 5.2
Repression Strength= 0.062
Traceability Scores = 1.00(IU) : 1.00(RU)

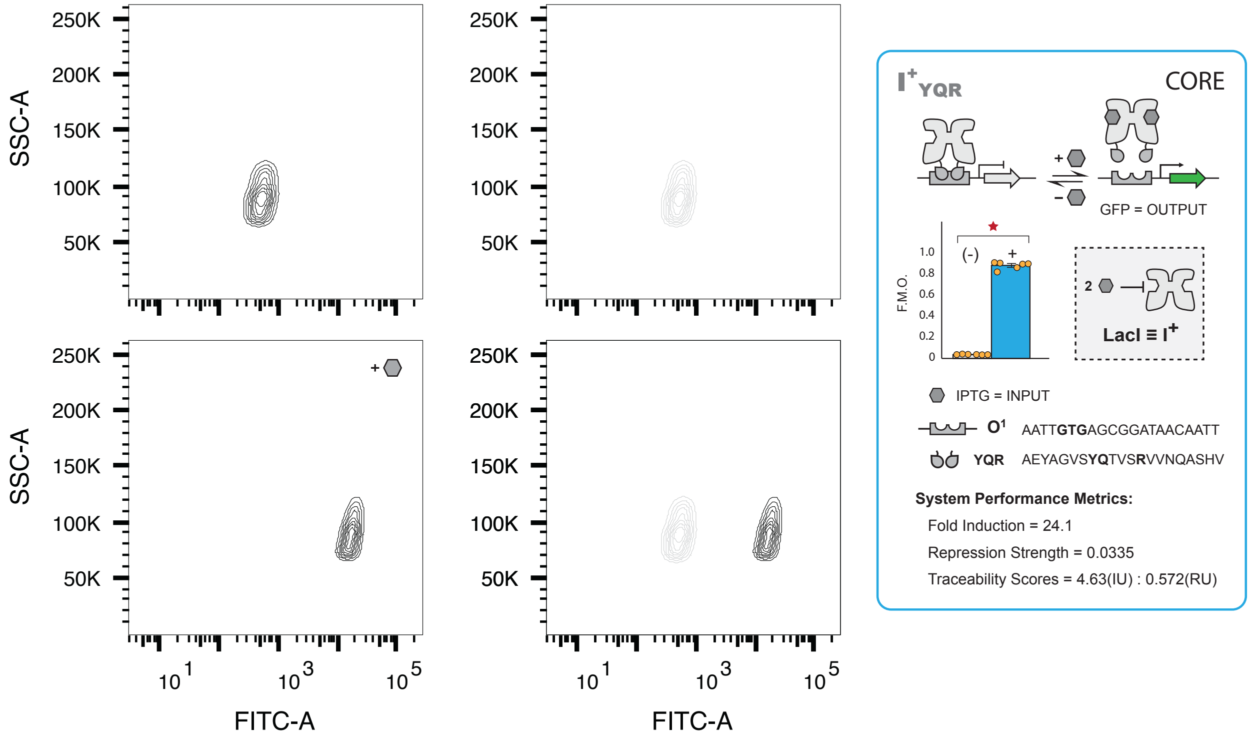
SSC-A
250K
200K
150K
100K
50K
10^1
10^3
10^5
FITC-A
FITC-A
I+YQR
CORE
GFP = OUTPUT
F.M.O.
(-)
+
2
LacI ≡ I+
IPTG = INPUT
O1 AATTGTGAGCGGATAACAATT
YQR AEYAGVSYQTVSRVVNQASHV
System Performance Metrics:
Fold Induction = 24.1
Repression Strength = 0.0335
Traceability Scores = 4.63(IU) : 0.572(RU)

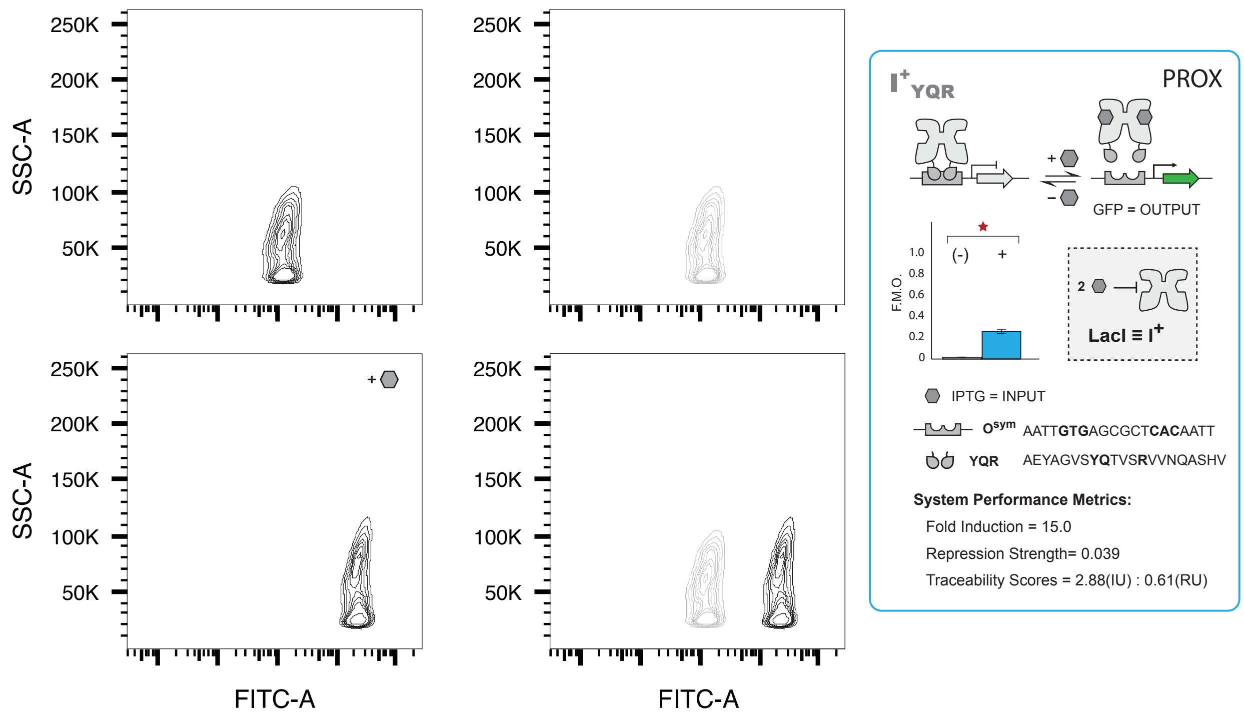
SSC-A
250K
200K
150K
100K
50K
FITC-A
I^{+}_{YQR}
PROX
GFP = OUTPUT
F.M.O.
(-)
+
2
LacI ≡ I^{+}
IPTG = INPUT
O^{sym} AATTGTGAGCGCTCACAATT
YQR AEYAGVSYQTVSRVVNQASHV
System Performance Metrics:
Fold Induction = 15.0
Repression Strength= 0.039
Traceability Scores = 2.88(IU) : 0.61(RU)

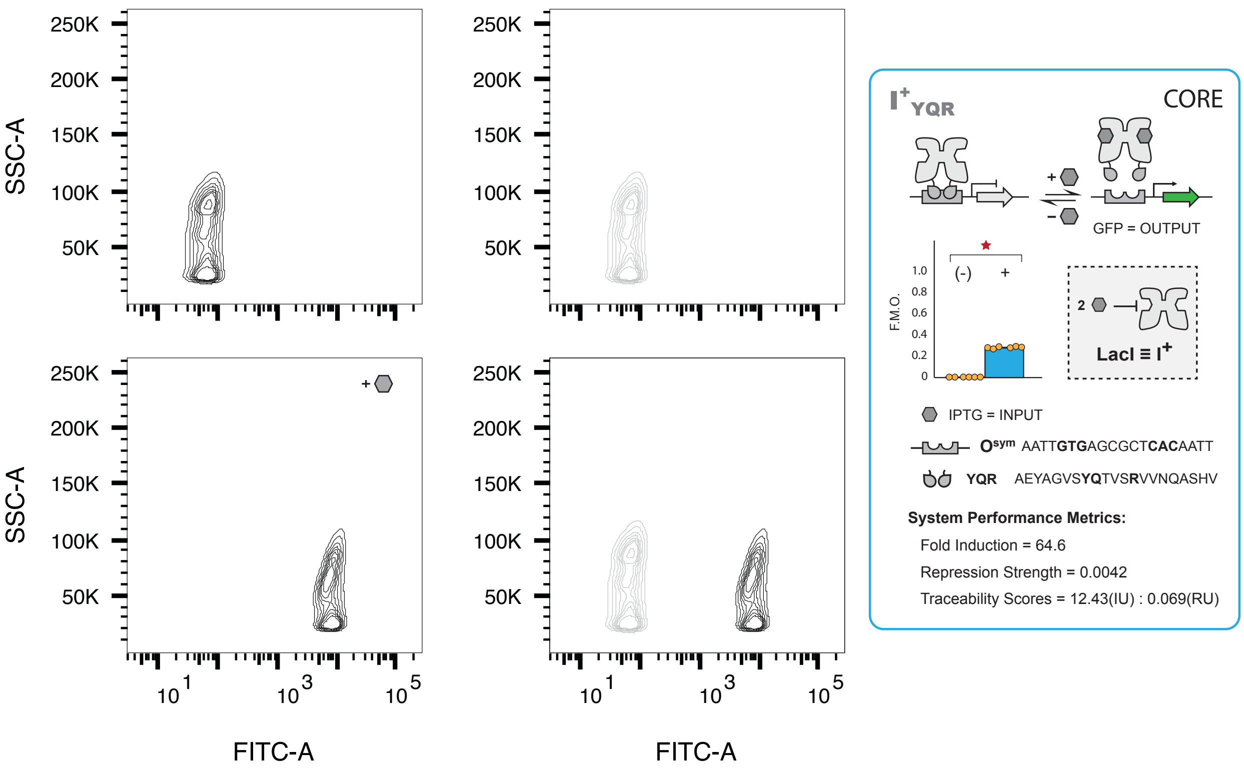
SSC-A
250K
200K
150K
100K
50K
10^1
10^3
10^5
FITC-A
I^{+}_{YQR}
CORE
GFP = OUTPUT
F.M.O.
(-)
+
2
LacI ≡ I^{+}
IPTG = INPUT
O^{sym} AATTGTGAGCGCTCACAATT
YQR AEYAGVSYQTVSRVVNQASHV
System Performance Metrics:
Fold Induction = 64.6
Repression Strength = 0.0042
Traceability Scores = 12.43(IU) : 0.069(RU)

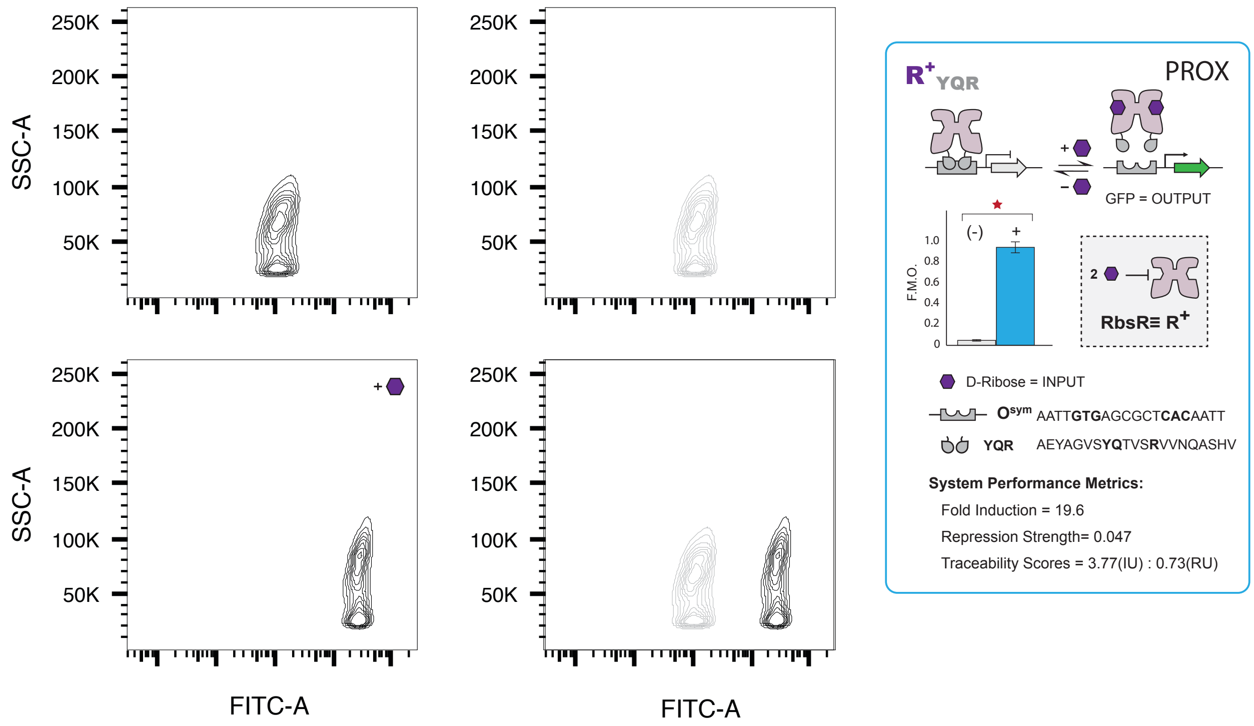

SSC-A
250K
200K
150K
100K
50K
FITC-A
R+ YQR
PROX
GFP = OUTPUT
F.M.O.
(-)
+
2
RbsR≡ R+
D-Ribose = INPUT
Osym AATTGTGAGCGCTCACAATT
YQR AEYAGVSYQTVSRVVNQASHV
System Performance Metrics:
Fold Induction = 19.6
Repression Strength= 0.047
Traceability Scores = 3.77(IU) : 0.73(RU)

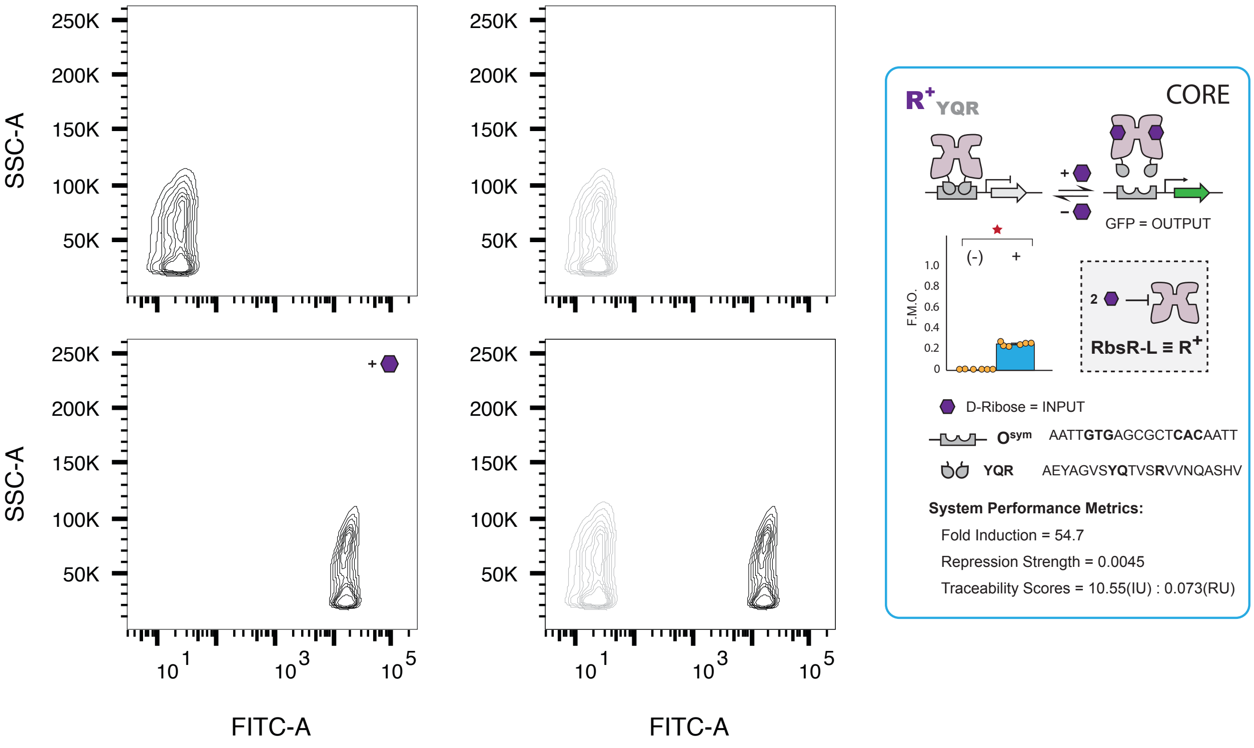

SSC-A
250K
200K
150K
100K
50K
10 1
10 3
10 5
FITC-A
R+ YQR
CORE
GFP = OUTPUT
F.M.O.
(-)
+
2
RbsR-L ≡ R+
D-Ribose = INPUT
Osym AATTGTGAGCGCTCACAATT
YQR AEYAGVSYQTVSRVVNQASHV
System Performance Metrics:
Fold Induction = 54.7
Repression Strength = 0.0045
Traceability Scores = 10.55(IU) : 0.073(RU)

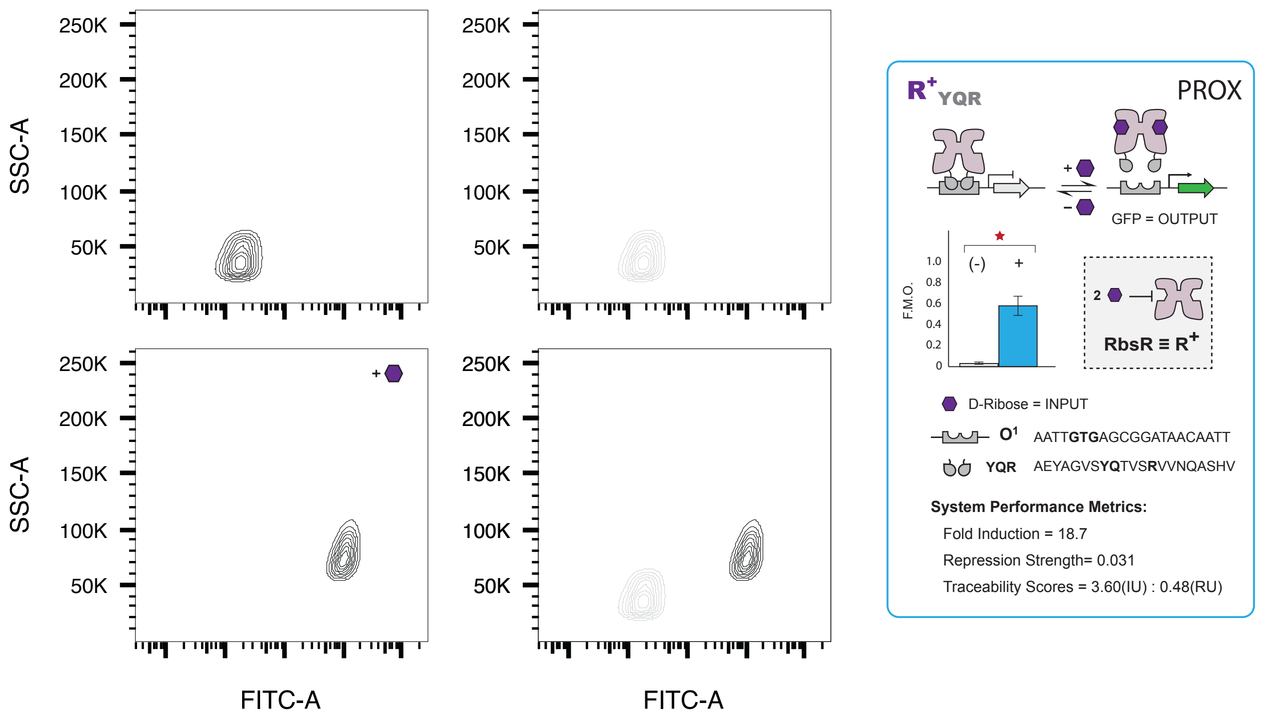
SSC-A
FITC-A
250K
200K
150K
100K
50K
R+ YQR
PROX
GFP = OUTPUT
F.M.O.
(-)
+
1.0
0.8
0.6
0.4
0.2
0
2
RbsR ≡ R+
D-Ribose = INPUT
O1 AATTGTGAGCGGATAACAATT
YQR AEYAGVSYQTVSRVVNQASHV
System Performance Metrics:
Fold Induction = 18.7
Repression Strength= 0.031
Traceability Scores = 3.60(IU) : 0.48(RU)

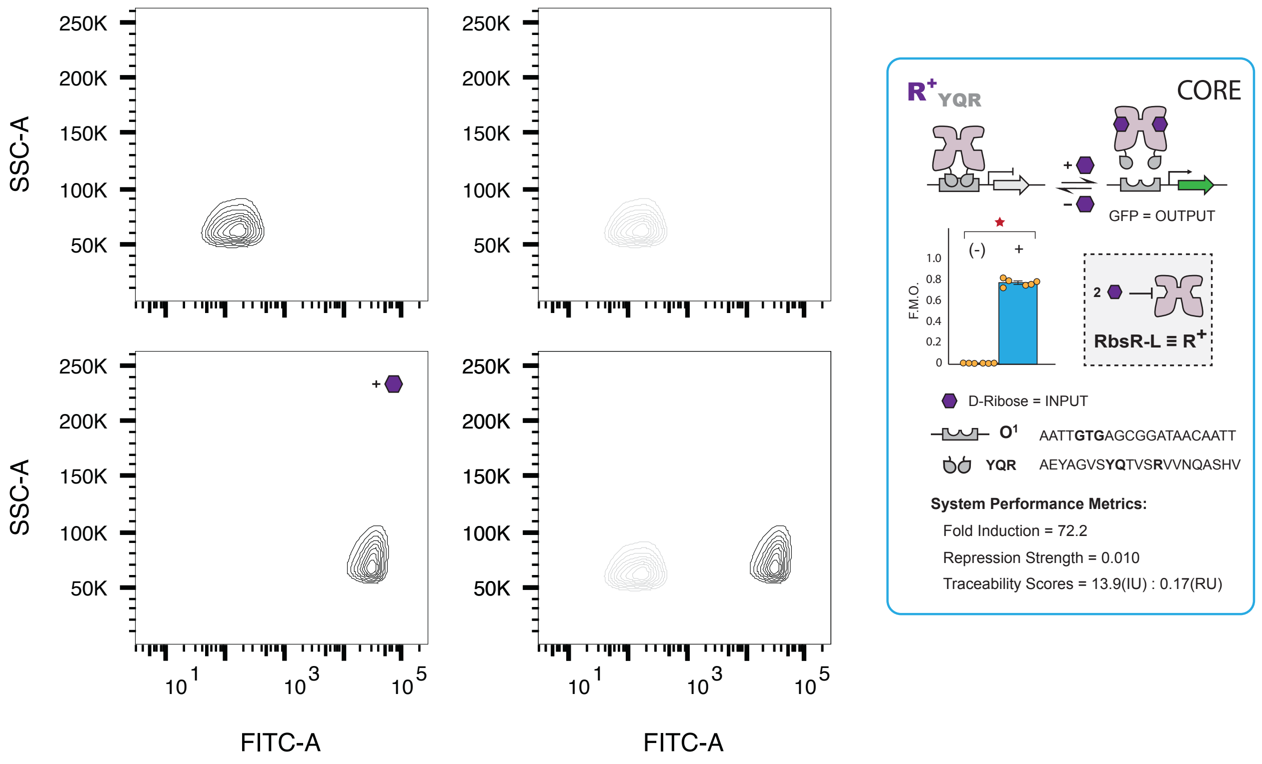
SSC-A
FITC-A
250K
200K
150K
100K
50K
10 1
10 3
10 5
R+ YQR
CORE
GFP = OUTPUT
F.M.O.
(-)
+
1.0
0.8
0.6
0.4
0.2
0
2
RbsR-L ≡ R+
D-Ribose = INPUT
O1 AATTGTGAGCGGATAACAATT
YQR AEYAGVSYQTVSRVVNQASHV
System Performance Metrics:
Fold Induction = 72.2
Repression Strength = 0.010
Traceability Scores = 13.9(IU) : 0.17(RU)

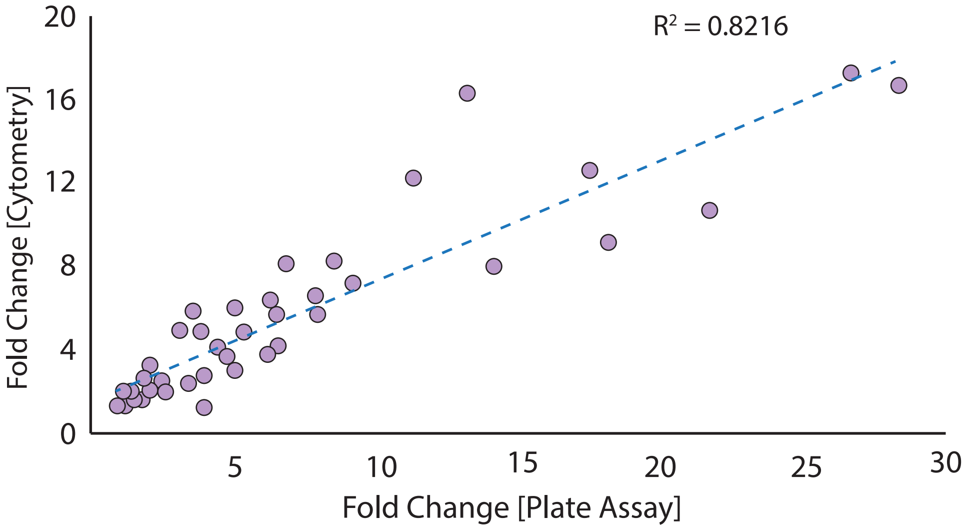

Supplementary Figure 9: Flow cytometry analysis of biological unit operations (BUO). A subset of biological unit operations were selected for flow cytometry analysis, comprising regulatory cores $R^{A(1)}$, $R^{A(2)}$, $R^{A(3)}$, $R^{A(4)}$, $F^{A(1)}$, $F^{A(2)}$ with cognate DBDs for operators O^1, O^{ttg}, and O^{agg}, along with any other single-TF BUO used to construct logical operations in this study. For each BUO, contour plots (with level gradient 10%) are shown for cytometry of cells in the absence and presence of ligand for at least 25,000 gated events. For each set, four contour plots are shown: (*top-left*) BUO without inducer, (*bottom-left*) BUO with inducer, (*top-right*), BUO without inducer, grayed out, to be compared to (*bottom-right*) BUO with inducer, with BUO without inducer also superimposed. Left axis represents side-scatter area (SSC-A) and bottom axis represents FITC-Area (FITC-A), detecting GFP fluorescence (log scale). To the right, the performance card for the BUO is shown, for reference. See **Supplementary Fig. 8** for details on performance cards. On cards, vertical bars display mean (defining the measure of centre) fraction of maximum output (F.M.O.) ±1 S.D. from n = 6 biological replicates, with dots shown for each replicate. Red stars denote statistically-significant differences in expression between the without (*left bar*) and with (*right bar*) ligand states for the operation in a Student's two-tailed t-test (α = 0.001).

Supplementary Figure 10

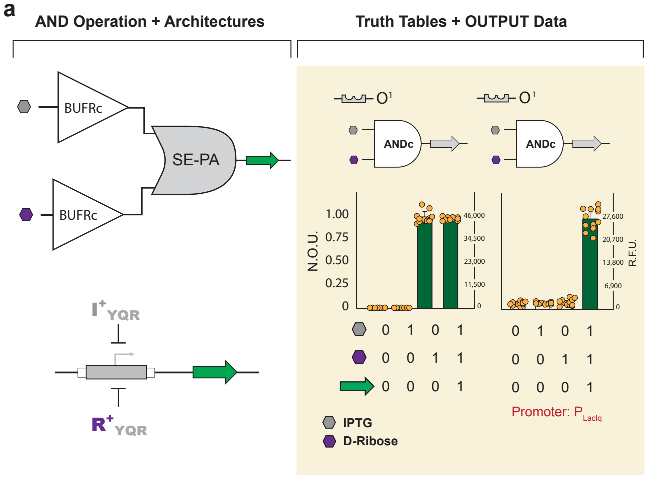

Chassis: 3.32 *E. coli* cells

Transciption Factor Plasmid:

Promoter: P_{LacIq}

Origin of Replication (copy number): p15A (18-22)

Resistance Marker: Chloramphenicol

Promoter

OPcore

OUTPUT

Reporter Plasmid:

Promoter: *trc* promoter

Origin of Replication (copy number): pSC101* (~5)

Resistance Marker: Kanamycin

RBS Sequence: AAAGAGGAGAAA

GFP = OUTPUT

y-axis = **F**raction of **M**aximum **O**utput, **F.M.O.**

Maximum **O**utput = 75,000 r.f.u.

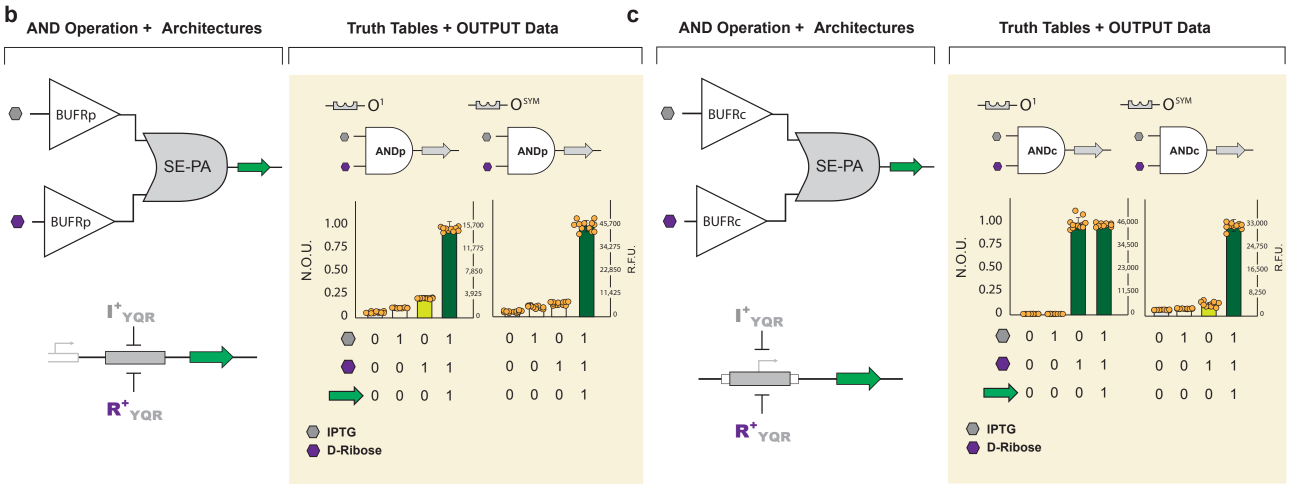

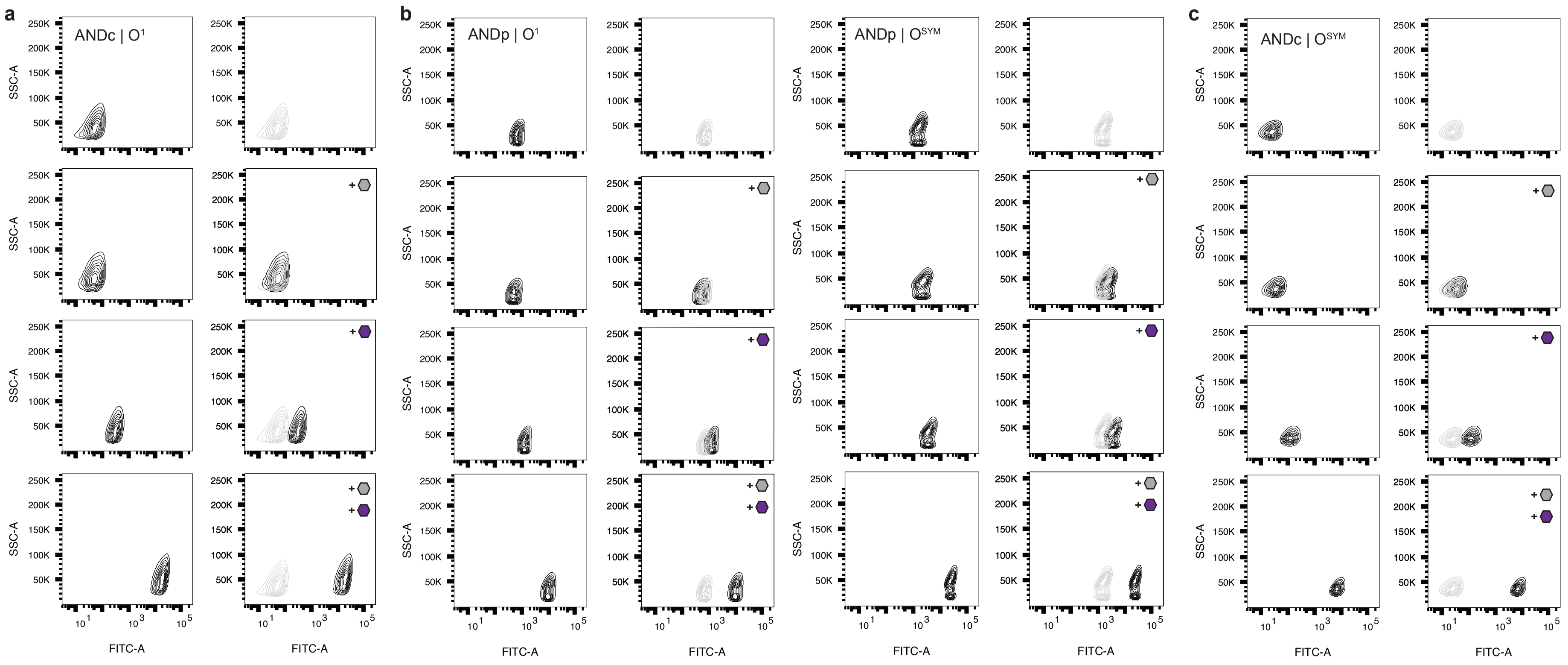
a
ANDc | O1
b
ANDp | O1
ANDp | OSYM
c
ANDc | OSYM
SSC-A
FITC-A
250K
200K
150K
100K
50K
10 1
10 3
10 5

Supplementary Figure 10: Transcriptional AND gates. AND gates may be constructed using combinations of BUFFER operations, here, highlighting the series-parallel (SE-PA) architecture. In plots, vertical bars display means (defining the measure of centre) ±1 S.D., Left axis denotes normalized output units (N.O.U.), the standardized mean fluorescence intensity as a fraction of the maximum observed for that operation and the right axes denotes relative (raw) fluorescence units (R.F.U.). Truth tables are shown, below, corresponding to bars, above, for different ligand conditions. Results shown are from n = 12 biological replicates, with each replicate shown as a dot. Below plots (data generated by microplate assay), is additionally flow cytometry analysis for the BUO in different ligand conditions, shown as contour plots (with level gradient 10%) for at least 25,000 gated events. Left axis represents side-scatter area (SSC-A) and bottom axis represents FITC-Area (FITC-A), detecting GFP fluorescence (log scale). **(a)** ANDc gates were constructed from two BUFRc operations. Here, I^{+}_{YQR} and R^{+}_{YQR} both regulated GFP *via* a core O^1 operator. *Left*, using $O^{1(core)}$ without modification did not result in an AND phenotype for this logical operation. Upon modification of the LacI promoter to the stronger $LacI^Q$ promoter on LacI, however, the AND operation was realized. This was also done by using the O^{SYM} symmetric (higher affinity) operator, see **(c). (b)** ANDp gates were constructed from two BUFRp operations. I^{+}_{YQR} and R^{+}_{YQR} both regulated GFP *via* a proximal O^1 (*left*) or O^{SYM} (*right*) operator. Only in the presence of both ligands, IPTG and D-ribose, was repression from both TFs relieved, allowing transcription. **(c)** ANDc gates were constructed from two BUFRc operations. Here, I^{+}_{YQR} and R^{+}_{YQR} both regulated GFP *via* a core O^1 (*left*) or O^{SYM} (*right*) operator. Results with the O^1 operator did not show AND behavior, see **(a)**, while O^{SYM} allowed realization of the AND operation.

Supplementary Figure 11

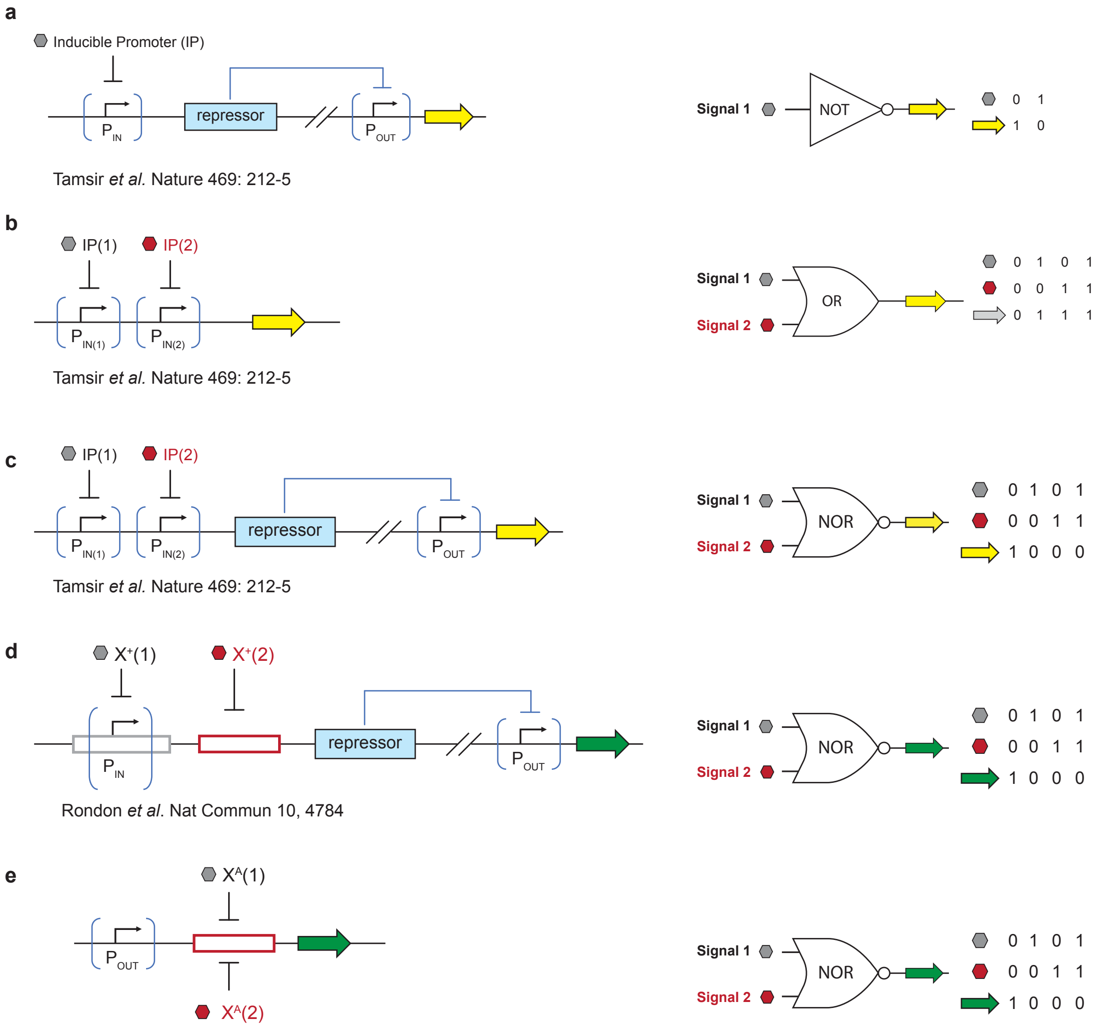

Supplementary Figure 11: Development of the transcriptional NOR operation. **(a-c)** The workflow for the engineering of the first generation of NOR transcriptional logic. **(a)** The NOT operation was constructed using a single inducible promoter, P_{IN} (through IP, an implicit X^+ repressor protein) to regulate a repressor (which acted as a simple inverter). In turn, this repressor interacted with P_{OUT} to regulate OUTPUT expression. Thus, in the presence of the IP ligand, repressor was expressed to inhibit transcription of OUTPUT. **(b)** The OR operation was constructed from two inducible promoters $P_{IN(1)}$ and $P_{IN(2)}$ (each through an implicit X^+ repressor protein). When either of these tandem promoters were induced by IP(1) or IP(2), transcription was permitted, resulting in OUTPUT expression. **(c)** Inversion of the OR operation resulted in the development of the NOR operation. The first generation NOR was constructed using two layers with three promoters. $P_{IN(1)}$ and $P_{IN(2)}$ (implicit repressors) regulate expression of an uninduced repressor, which, in turn, regulates expression of OUTPUT from a third promoter, P_{OUT}. In the presence of any, or both, ligands corresponding to $P_{IN(1)}$ and $P_{IN(2)}$, the repressor is expressed, which inhibits P_{OUT}. **(d)** In the second generation, NOR was constructed using two layers with two promoters. P_{IN} drives expression of a repressor, which is additionally regulated by an operator (red box). Regulation is achieved with X^+ repressors. A third, uninduced repressor then regulates expression of OUTPUT *via* P_{OUT}. When either, or both, ligands are present, a sufficient amount of repressor is produced to diminish gene OUTPUT. **(e)** In the third generation, we have reduced the functionally-complete NOR operation to a single promoter in one layer using engineered anti-repressors. OUTPUT, driven by P_{OUT}, is directly regulated by two anti-repressors using one operator (here, shown in the proximal position). Only in the presence of neither ligand is the OUTPUT not anti-induced by the two X^A transcription factors.

Supplementary Figure 12

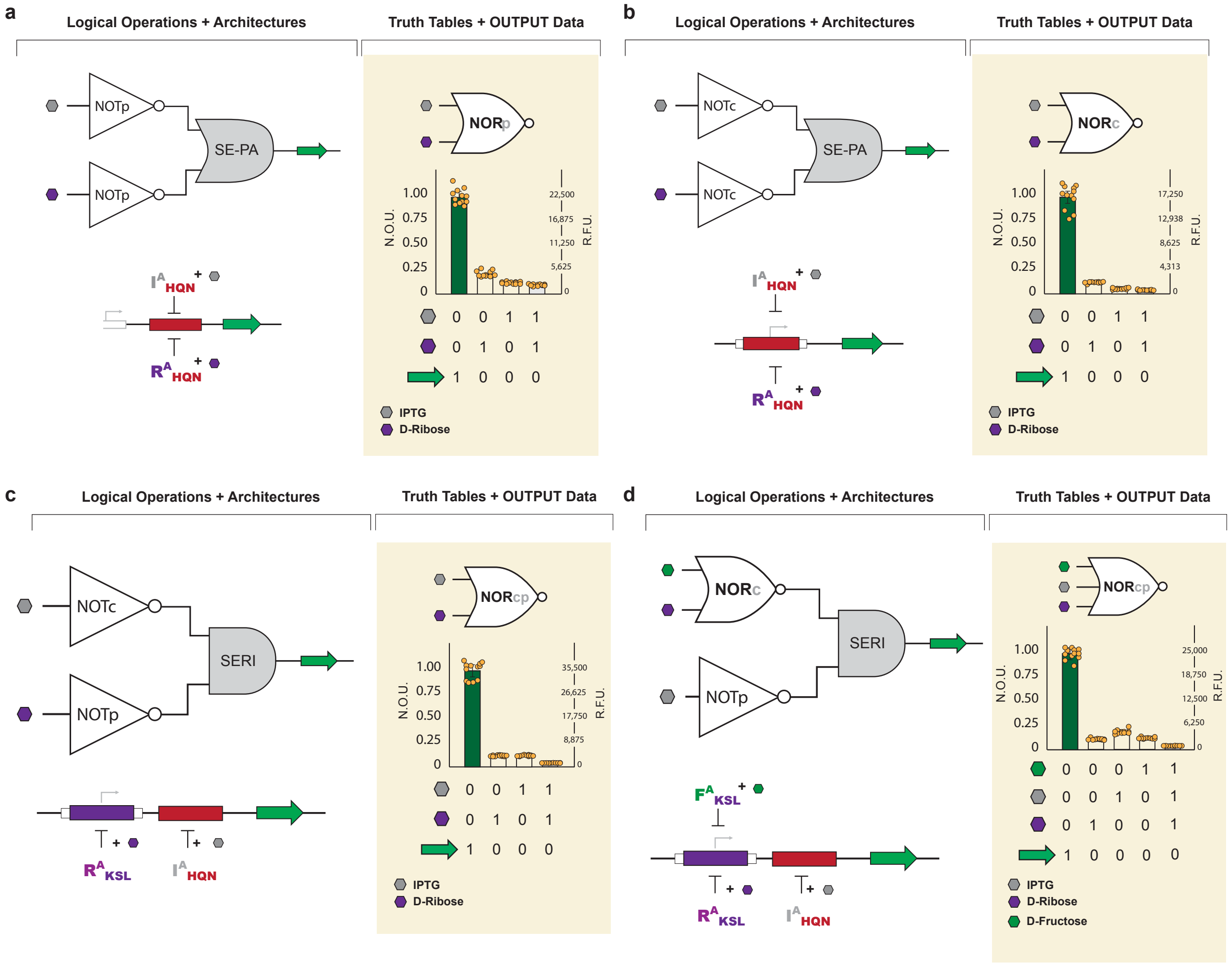

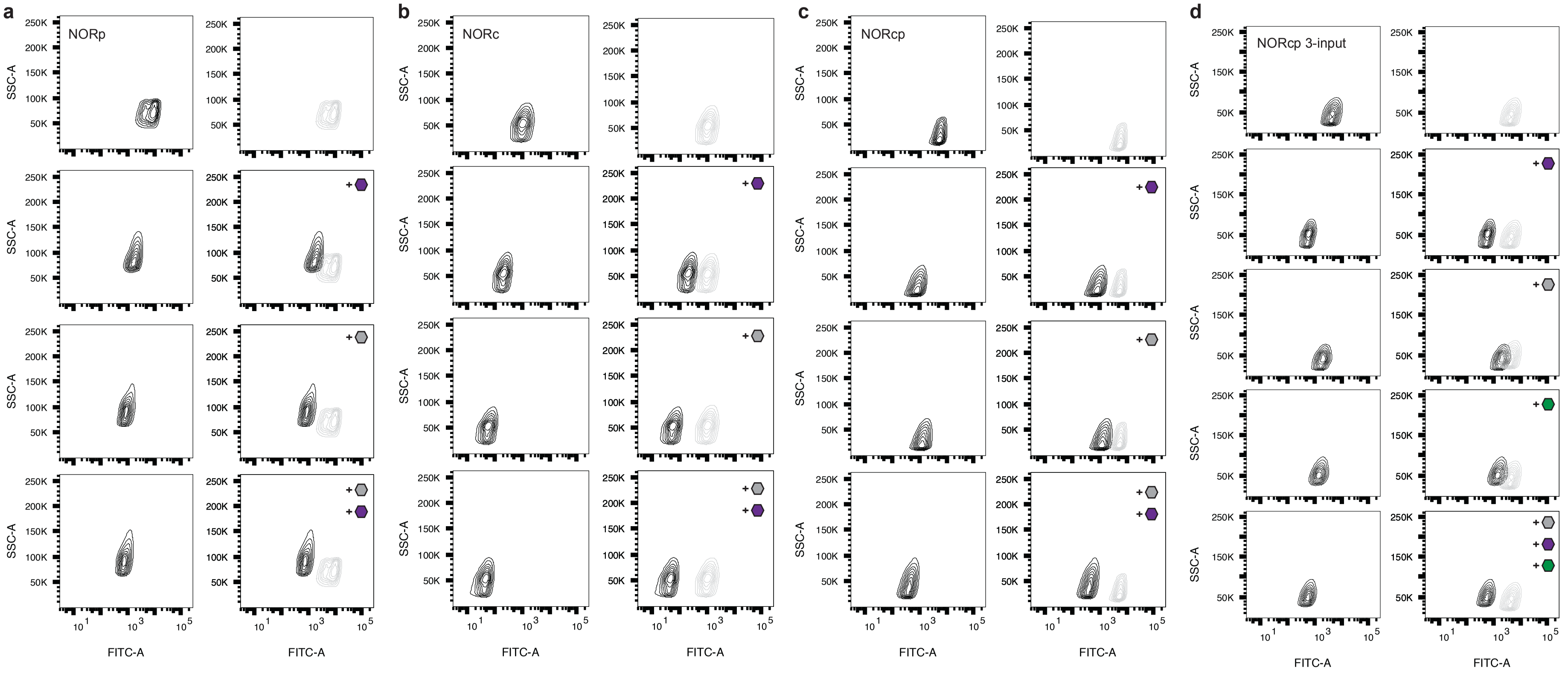

a
NORp
b
NORc
c
NORcp
d
NORcp 3-input
SSC-A
FITC-A

Supplementary Figure 12: Transcriptional NOR logic gates. NOR gates may be constructed using combinations of NOT operations in either series-parallel (SE-PA), **(a-b)** or series (SERI) **(c-d)** genetic architectures. In plots, vertical bars display means (defining the measure of centre) ±1 S.D. Left axes denotes normalized output units (N.O.U.), the standardized mean fluorescence intensity as a fraction of the maximum observed for that operation and the right axis denotes relative (raw) fluorescence units (R.F.U.). Truth tables are shown, below, corresponding to bars, above, for different ligand conditions. Results shown are from n = 12 biological replicates, with each replicate shown as a dot. Below plots (data generated by microplate assay), is additionally flow cytometry analysis for the BUO in different ligand conditions, shown as contour plots (with level gradient 10%) for at least 25,000 gated events. Left axis represents side-scatter area (SSC-A) and bottom axis represents FITC-Area (FITC-A), detecting GFP fluorescence (log scale). **(a)** NORp gates were constructed from two NOTp operations in SE-PA. $R^{A(1)}{}_{HQN}$ and $I^{A(5)}{}_{HQN}$ (from Rondon and Wilson[2]) both regulated GFP *via* a proximal O^{ttg} operator. In the presence of either, or both, ligands, IPTG and D-ribose, transcription was anti-induced, causing diminished gene expression. **(b)** By moving the O^{ttg} operator to the core position, a NORc gate was constructed from two NOTc operations. **(c)** A NORcp gate was constructed from two NOT operations in series – NOTc and NOTp. Here, $R^{A(1)}{}_{KSL}$ regulated a core O^{agg} operator and $I^{A(5)}$ independently regulated a proximal O^{ttg} operator, both upstream of the GFP reporter gene. Only in the presence of neither ligand was gene transcription unabated. **(d)** A three-input NORcp gate was constructed by the addition of the $F^{A(2)}{}_{KSL}$ transcription factor, which additionally regulated the core O^{agg} operator. Now, the presence of any of the three ligands, IPTG, D-ribose, and fructose, was sufficient to inhibit gene expression.

Supplementary Figure 13

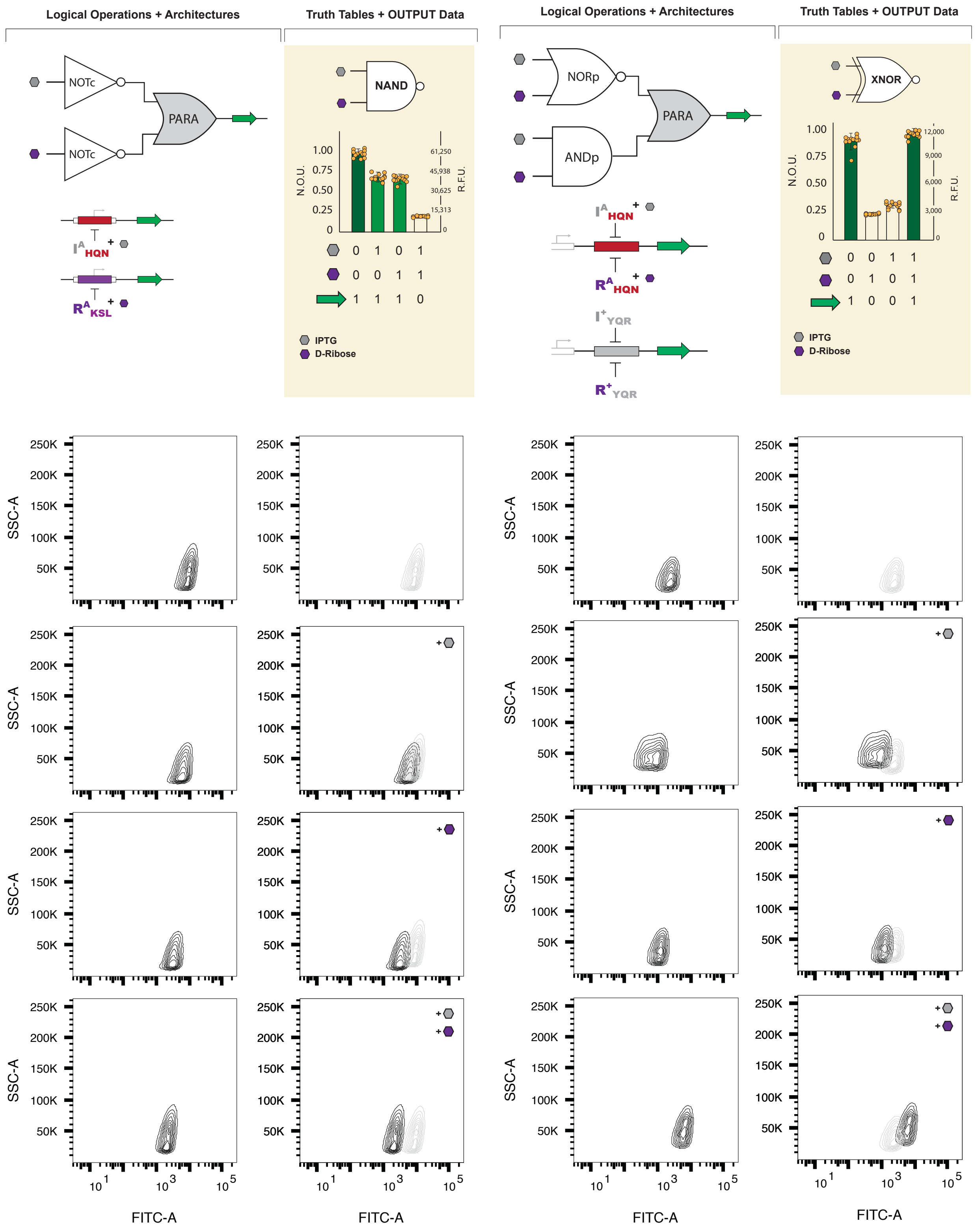

Supplementary Figure 13: Transcriptional NAND and XNOR gates. Combinatorial logic in the parallel (PARA) architecture allows the construction of the NAND and XNOR operations. In plots, vertical bars display means (defining the measure of centre) ±1 S.D. Left axes denotes normalized output units (N.O.U.), the standardized mean fluorescence intensity as a fraction of the maximum observed for that operation and the right axis denotes relative (raw) fluorescence units (R.F.U.). Truth tables are shown, below, corresponding to bars, above, for different ligand conditions. Results shown are from n = 12 biological replicates, with each replicate shown as a dot. Below plots (data generated by microplate assay), is additional flow cytometry analysis for the BUO in different ligand conditions, shown as contour plots (with level gradient 10%) for at least 25,000 gated events. Left axis represents side-scatter area (SSC-A) and bottom axis represents FITC-Area (FITC-A), detecting GFP fluorescence (log scale). **(a)** The NAND operation was achieved with two NOTc operations in PARA. In one channel, $I^{A(9)}_{HQN}$ (from Rondon and Wilson[2]) regulated GFP *via* a core O^{ttg} operator; in a second channel, $R^{A(1)}_{KSL}$ regulated GFP *via* a core O^{agg} operator. In the presence of neither ligand, transcription was fully-permitted in both channels (relative OUTPUT = 1), while in the presence of only one ligand, IPTG or D-ribose, one channel was anti-induced, but, in the other, transcription was fully-permitted (relative OUTPUT ~ 1). Only in the presence of both ligands were both channels anti-induced, leading to full inhibition of gene expression. **(b)** The XNOR operation was constructed with a NORp and ANDp operation in PARA. In one channel, $I^{A(9)}_{HQN}$ and $R^{A(1)}_{HQN}$ simultaneously regulated GFP *via* a proximal O^{ttg} operator in a SE-PA architecture; in the second channel, , I^{+}_{YQR} and R^{+}_{YQR} regulated GFP *via* a proximal O^{SYM} operator (see **Supplementary Fig. 8** for this individual BUO). In the presence of either no ligand present, or both ligands present, one channel fully permitted transcription, while the other was attenuated, leading to relative OUTPUT = 1. In the presence of only one ligand, however, both channels were attenuated, as simultaneously, either I^{+}_{YQR} and $R^{A(1)}_{HQN}$ or R^{+}_{YQR} and $I^{A(9)}_{HQN}$ were interacting with their respective operators, causing relative gene output to be diminished.

Supplementary Figure 14

Regulatory Core Domain (RCD)	DNA-binding Domain (DBD)	Phenotype	Note
RbsR	RbsR	R^+	wild-type RbsR
RbsR	LacI	R^+	R^+_{YQR} / RbsR-L
RbsR E82P	RbsR	R^A	
RbsR E82P	LacI	R^A	$R^{A(3)}_{YQR}$ / RbsR-L E84P
RbsR F86P	RbsR	R^-	
RbsR F86P	LacI	R^A	$R^{A(4)}_{YQR}$ / RbsR-L F88P
RbsR V93D	RbsR	R^S	
RbsR V93D	LacI	R^A	$R^{A(1)}_{YQR}$ / RbsR-L V95D
RbsR V93E	RbsR	R^S	
RbsR V93E	LacI	R^S	$R^S_{YQR(V95E)}$ / RbsR-L V95E
RbsR L94P	RbsR	R^S	
RbsR L94P	LacI	R^A	$R^{A(2)}_{YQR}$ / RbsR-L V96P
FruR	FruR	F^+	wild-type FruR
FruR	LacI	F^+	F^+_{YQR} / FruR-L
FruR L95I	FruR	F^+	
FruR L95I	LacI	F^+ ***	$F^S_{YQR(L95I)}$ / FruR-L L95I
FruR L95K	FruR	F^+	
FruR L95K	LacI	F^+ ***	$F^S_{YQR(L95K)}$ / FruR-L L95K
FruR I96P	FruR	F^S	
FruR I96P	LacI	F^S	$F^S_{YQR(I96P)}$ / FruR-L I96P
FruR I96R	FruR	F^S	
FruR I96R	LacI	F^S	$F^S_{YQR(I96R)}$ / FruR-L I96R
$F^{A(1)}$ (RCD)	FruR	F^+	
$F^{A(1)}$ (RCD)	LacI	F^A	$F^{A(1)}_{YQR}$ / FruR-$L^{A(1)}$
$F^{A(2)}$ (RCD)	FruR	F^+	
$F^{A(2)}$ (RCD)	LacI	F^A	$F^{A(2)}_{YQR}$ / FruR-$L^{A(2)}$

Supplementary Figure 14: Phenotypes of engineered and natural transcription factors. The phenotype for each transcription factor is shown for each pairing of a regulatory core domain (RCD) with a DNA-binding domain (DBD). X^+ corresponds to a repressor, X^S corresponds to a super-repressor, X^A corresponds to a suppressor, and X^- corresponds to a non-functional protein. F or R correspond to the RCD a transcription factor bears, deriving from RbsR (R) or FruR (F) and the ligand with which it was assayed, ribose or fructose, respectively. The stars indicate *weak* F^+ (diminished overall expression, retaining DNA-binding, but a statistically-significant increase in fluorescence upon induction) variants that were used successfully as F^S parents in the directed evolution route toward an F^A. “Note” signifies any alternative names for a variant used in this work, Rondon *et al.*[1], or Shis *et al.* [6].

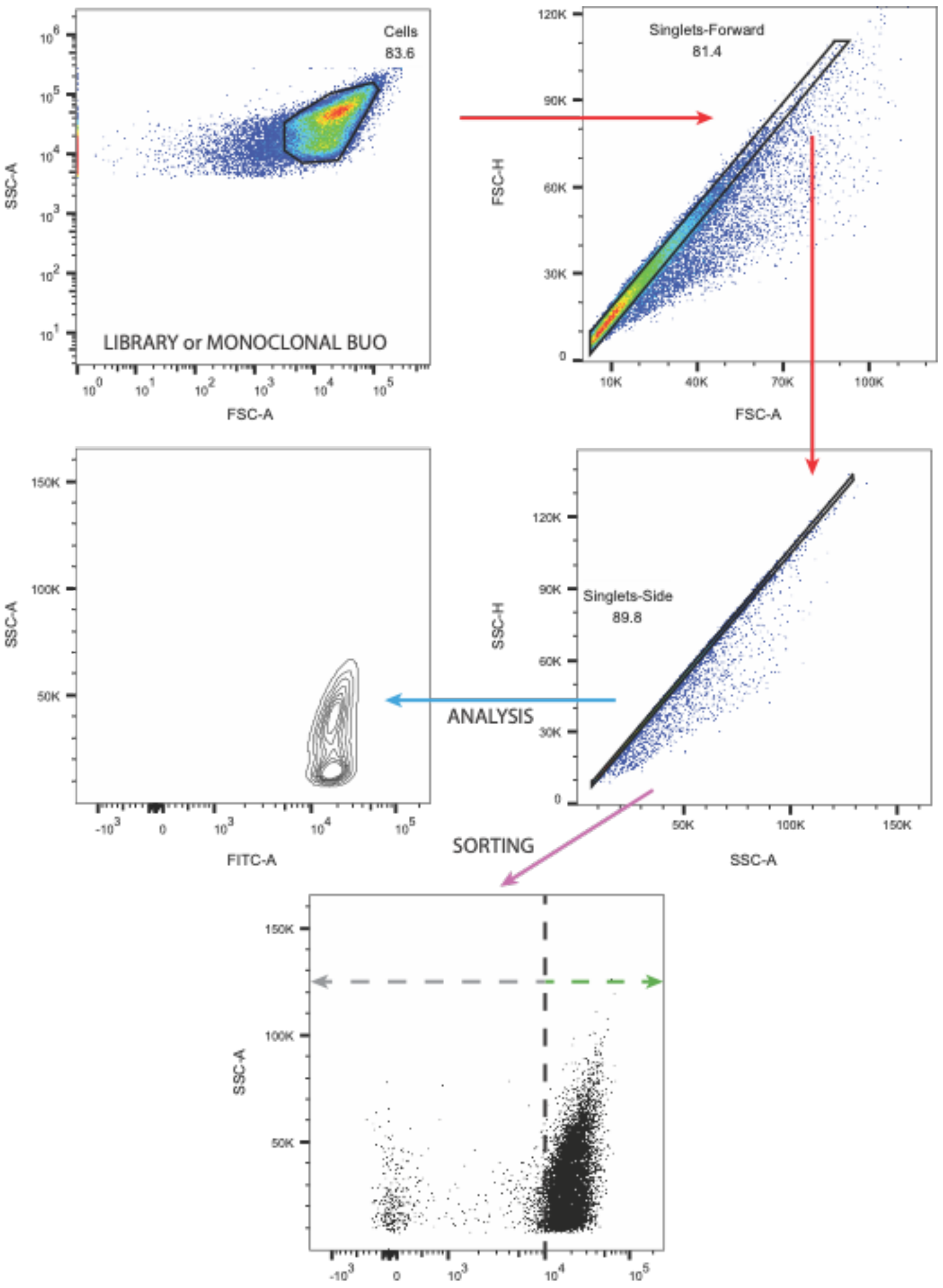

Supplementary Figure 15: Gating and sorting strategy for flow cytometry (analysis and cell sorting). Beginning with a library (for cell sorting) or BUO derived from a single colony (for analysis), gating was first performed to isolate cells for cytometry. *Upper left,* cells were first discriminated with a FSC-A v. SSC-A pseudocolor (or dot) plot, gating those events with a characteristic aspect ratio determined from daily and historic controls. These events were then sequentially discriminated (red arrows) with FSC-A v. FSC-H (*upper right*) and SSC-A v. SSC-H (*middle right*) pseudocolor (or dot) plots, choosing events on the diagonal of each to isolate singlets. For populations for analysis, a contour plot of FITC-A v. SSC-A was generated (contour levels at 10%; following blue arrow, *middle left*). For populations for sorting (magenta arrow), a dot plot of FITC-A v. SSC-A was generated (*bottom center*). In sorting protocols, bins were assigned as > 10^4 FITC-A for high-expression variants and < 10^4 FITC-A for low-expression variants. This cutoff is delineated with a black dashed line, with green and gray arrows indicating the two bins, respectively. See **Methods** for further details on sorting strategy. The above strategy was used on all flow cytometry data presented in **Supplementary Figs. 9, 10, 12** and **13.**

REFERENCES

1. Rondon, R.E., Groseclose, T.M., Short, A.E. & Wilson, C.J. Transcriptional programming using engineered systems of transcription factors and genetic architectures. *Nat Commun* **10**, 4784 (2019).
2. Rondon, R.E. & Wilson, C.J. Engineering a New Class of Anti-LacI Transcription Factors with Alternate DNA Recognition. *ACS Synth Biol* **8**, 307-317 (2019).
3. Richards, D.H., Meyer, S. & Wilson, C.J. Fourteen Ways to Reroute Cooperative Communication in the Lactose Repressor: Engineering Regulatory Proteins with Alternate Repressive Functions. *ACS Synth Biol* **6**, 6-12 (2017).
4. Suckow, J. *et al.* Genetic studies of the Lac repressor. XV: 4000 single amino acid substitutions and analysis of the resulting phenotypes on the basis of the protein structure. *J Mol Biol* **261**, 509-523 (1996).
5. Meyer, S. *et al.* Engineering alternate cooperative-communications in the lactose repressor protein scaffold. *Protein Eng Des Sel* **26**, 433-443 (2013).
6. Shis, D.L., Hussain, F., Meinhardt, S., Swint-Kruse, L. & Bennett, M.R. Modular, multi-input transcriptional logic gating with orthogonal LacI/GalR family chimeras. *ACS Synth Biol* **3**, 645-651 (2014).
7. Ramseier, T.M. *et al.* In vitro binding of the pleiotropic transcriptional regulatory protein, FruR, to the fru, pps, ace, pts and icd operons of Escherichia coli and Salmonella typhimurium. *J Mol Biol* **234**, 28-44 (1993).
