# Supplementary material for: Engineered systems of inducible anti-repressors for the next generation of biological programming
Source: Nat Commun. 2020 Sep 7;11:4440. doi: 10.1038/s41467-020-18302-1 (PMC7477573; doi:10.1038/s41467-020-18302-1)
Supplement: Supplementary file 2 — Reporting Summary [file 41467_2020_18302_MOESM2_ESM.pdf]

nature research

Corresponding author(s): Corey J. Wilson

Last updated by author(s): Aug 4, 2020

Reporting Summary

Nature Research wishes to improve the reproducibility of the work that we publish. This form provides structure for consistency and transparency in reporting. For further information on Nature Research policies, see Authors & Referees and the Editorial Policy Checklist .

Statistics

For all statistical analyses, confirm that the following items are present in the figure legend, table legend, main text, or Methods section.

n/a	Confirmed	
☐	☒	The exact sample size (n) for each experimental group/condition, given as a discrete number and unit of measurement
☐	☒	A statement on whether measurements were taken from distinct samples or whether the same sample was measured repeatedly
☐	☒	The statistical test(s) used AND whether they are one- or two-sided<br>*Only common tests should be described solely by name; describe more complex techniques in the Methods section.*
☒	☐	A description of all covariates tested
☐	☒	A description of any assumptions or corrections, such as tests of normality and adjustment for multiple comparisons
☐	☒	A full description of the statistical parameters including central tendency (e.g. means) or other basic estimates (e.g. regression coefficient) AND variation (e.g. standard deviation) or associated estimates of uncertainty (e.g. confidence intervals)
☐	☒	For null hypothesis testing, the test statistic (e.g. F, t, r) with confidence intervals, effect sizes, degrees of freedom and P value noted<br>*Give P values as exact values whenever suitable.*
☒	☐	For Bayesian analysis, information on the choice of priors and Markov chain Monte Carlo settings
☒	☐	For hierarchical and complex designs, identification of the appropriate level for tests and full reporting of outcomes
☐	☒	Estimates of effect sizes (e.g. Cohen's d, Pearson's r), indicating how they were calculated

Our web collection on statistics for biologists contains articles on many of the points above.

Software and code

Policy information about availability of computer code

Data collection	Clustal Omega (version 1.2.4), EMBOSS Needle (version 6.6.0), BD FACSDiva (8.0.1), Molecular Devices SoftMax Pro (version 7.0.3)
Data analysis	Microsoft Excel (16.0), BD FlowJo (version 10.6.2), ApE Plasmid Editor (version 2.0.51), SnapGene (version 5.0.7)

For manuscripts utilizing custom algorithms or software that are central to the research but not yet described in published literature, software must be made available to editors/reviewers. We strongly encourage code deposition in a community repository (e.g. GitHub). See the Nature Research guidelines for submitting code & software for further information.

Data

Policy information about availability of data

All manuscripts must include a data availability statement. This statement should provide the following information, where applicable:

- Accession codes, unique identifiers, or web links for publicly available datasets
- A list of figures that have associated raw data
- A description of any restrictions on data availability

The authors declare that all data supporting the findings of this study are available within the paper and its supplementary information. The analyzed data and source data will be made available in Supplementary Data Files and Source Data. Any other information can be made available from the corresponding author upon reasonable request. The sequences of the following plasmids are provided in GenBank: Proximal Reporter Plasmids (MN207964 - MN207971), Core Reporter Plasmids (MT127263 - MT127272), anti-FruR Plasmids (MT127340 - MT127357), anti-RbsR Plasmids (MT127280 - MT127308), wild-type FruR Plasmids (MT127333 - MT127339), wild-type RbsR Plasmids (MT127309 - MT127314), F+YQR Plasmid (MN207916), R+YQR Plasmid (MN207958), anti-LacI Plasmids (MT127315 - MT127332), dual-TF Plasmids (MT127275 - MT127279), pLacI (MT127274), pLacNULL Plasmid (MT127273), pXNOR (MT127262), pNORcp (MT127261), and pNAND (MT127260). Protein sequences for wild-type LacI, RbsR, and FruR proteins can be found in the UnitProt database with accession numbers: FruR (#P0ACP1), RbsR (#P0ACQ0), and LacI (#P03023).

Field-specific reporting

Please select the one below that is the best fit for your research. If you are not sure, read the appropriate sections before making your selection.

- [x] Life sciences
- [ ] Behavioural & social sciences
- [ ] Ecological, evolutionary & environmental sciences

For a reference copy of the document with all sections, see nature.com/documents/nr-reporting-summary-flat.pdf

Life sciences study design

All studies must disclose on these points even when the disclosure is negative.

Sample size	Previous work in our lab (Rondon and Wilson, ACS Synth. Biol., 2019; Rondon, et al., Nat. Comm., 2019) utilized a sample size of n = 6 for phenotyping of transcription factor variants, as we have done here. We have demonstrated internally our ability to replicate results in this publication and previous ones with n = 6 biological replicates, including between different days. We chose to increase sample size to n = 12 for genetic logic gates to increase our power in statistical hypothesis testing. Post-hoc power analyses have also been performed to verify a desirable power for phenotyping purposes.
Data exclusions	No data has been excluded from analyses. All data collected is shown on plots and is included in the Source Data file.
Replication	All attempts at replication were successful. Data for anti-repression matrices were gathered over 2 (or more) days (for each condition) and, internally, testing for phenotype and performance shows repeatability from day-to-day (within +/- 1 standard deviation). Logic gate assaying shows similar repeatability with assaying occurring over 2 (or more) days (for each condition) and several colony forming units (cfus) were selected to verify phenotype when assaying (but note, only data for one cfu is reported in publication). In all cases, all cfus exhibited the same phenotype. All experiments were independently repeated over 2 (or more) days. Additionally, all phenotyped single TF interactions (e.g., NOT experiments) exhibited the same phenotype when paired with other TFs to construct logical operations. Additionally, cell cytometry analysis, where appropriate, was measured (for each condition) over 2 days.
Randomization	For site-saturation mutagenesis, resultant colonies following PCR and transformation were chosen/picked at random (sampling 3x for a 95% probability of obtaining all amino acids in the library). Similarly, resultant colonies following FACS were chosen at random for plate assay to verify phenotype. All transformants were included in FACS sorting (streaked from selection plates). In all cloning experiments (to construct genetic logic, reporter, and modified TFs), resultant colonies following PCR and transformation were chosen at random for sequencing, to verify correct construction. For phenotyping (single TF) and logic gate testing (multiple TF), colonies were chosen at random for testing at varying conditions. Colonies were likewise chosen at random from transformations from isolated DNA from site-saturation libraries (following initial screening) to verify phenotype. Phenotypes/performance was verified by testing multiple cfus (over 2 or more days). The same colony reported for microplate assay was chosen for cell cytometry analysis, where appropriate. Transformations were performed only with sequence-verified DNA (not chosen at random), which was essential to ensure proper BUO performance/behavior.
Blinding	Blinding is not applicable in this study. For a given biological unit operation, it was essential for the authors to know the bacteria genotype (bearing which plasmid(s)) for proper testing. Namely, following randomization (per above) of plated colonies of a specific transformation (with specific plasmids), colonies had to then be cultured and assayed in conditions particular to that BUO: antibiotic resistance, ligand(s) to be added, and number of biological replicates to perform. It was required that the authors know which reagents to add to the media to evaluate each BUO (and to then report this). Additionally, all groups were subjected to all conditions evaluated - i.e., a colony of a given genotype was subjected to the control condition (without ligand), as well as with any ligand(s) that were relevant.

Reporting for specific materials, systems and methods

We require information from authors about some types of materials, experimental systems and methods used in many studies. Here, indicate whether each material, system or method listed is relevant to your study. If you are not sure if a list item applies to your research, read the appropriate section before selecting a response.

Materials & experimental systems

n/a	Involved in the study	
x		Antibodies
x		Eukaryotic cell lines
x		Palaeontology
x		Animals and other organisms
x		Human research participants
x		Clinical data

Methods

n/a	Involved in the study	
x		ChIP-seq
	x	Flow cytometry
x		MRI-based neuroimaging

Flow Cytometry

Plots

Confirm that:

- [x] The axis labels state the marker and fluorochrome used (e.g. CD4-FITC).
- [x] The axis scales are clearly visible. Include numbers along axes only for bottom left plot of group (a 'group' is an analysis of identical markers).
- [x] All plots are contour plots with outliers or pseudocolor plots.
- [x] A numerical value for number of cells or percentage (with statistics) is provided.

Methodology

Sample preparation	Transformed 3.32 E. coli cells (bearing desired genetic logic gate architecture) were pre-cultured in LB Broth with antibiotic(s) overnight. The next day, cells were diluted 1:100 in supplemented M9 minimal media with relevant antibiotic(s) and inducer(s), then grown for 20 hr, shaking at 300 rpm and 37 C. Each variant was then diluted to OD600 = 0.2, pelleted by centrifuging at 17,500g for 2 min, then resuspended in PBS supplemented with HEPES, EDTA, and Tween20. This washing step was repeated once. Cells were then finally resuspended in PBS supplemented with HEPES and EDTA (in 1 mL aliquots). Preparations were performed on ice. See Methods for details.
Instrument	BD Biosciences FACSAria Fusion
Software	BD FACSDiva (version 8.0.1), BD FlowJo (version 10.6.2)
Cell population abundance	For analysis experiments, all cells were genotypically/phenotypically identical (as cultures were grown from single cfus). After gating strategies, these populations were 99% or greater pure, based off of expected phenotypes and controls for fluorescence (FITC-A) intensity. Histograms and dot plots were evaluated based off purity, as well as events that did not gate appropriately.<br><br>For sorting experiments, post-sort fractions were evaluated based off of purity on the same day and in a subsequent sort. For a given sort condition (with or without ligand), sorting was performed twice, with the second to evaluate purity of the first sort, after the population had been grown up (enriched). Additionally, on the same day as a sort occurred, a small volume (approximately 5-10 uL) was taken and analyzed for purity (determined if event fell in the previously-sorted gate). Purity sorts on the same day were, on average, 70-90% pure (evaluating from events falling into a previously-sorted gate). Sorts from enriched populations (from a sort on a previous day) were, on average, 60-70% pure (maintaining same gate). Many events falling outside the gate on a subsequent day, however, were within 3 units on log-scale (+/- factor of 3), but no statistics or analyses were performed on this population. We anticipate these are individual cells that are varying within standard error measures of the population.
Gating strategy	Events were gated on forward- and side-scatter and a threshold was set for side-scatter, with doublets discriminated against using standard SSC-A vs. FSC-A, FSC-A vs. FSC-H, and SSC-A vs. SSC-H plots. FSC-A vs. SSC-A gates were selected to capture a representative collection of the total events (approximately >10E3 FSC-A and SSC-A) and FSC-A vs. FSC-H and SSC-A vs. SSC-H plots were used to gate along the "diagonal" line, hierarchically discriminating for our cells' physical characteristics.<br><br>Fluorescence gates were initially determined from controls - cells with lacnull plasmids, plasmids bearing well-characterized super-repressors and antilac transcription factors, and GFPnull plasmids (giving maximum, minimum, and intermediate expression of GFP - defined "positive" and "negative" staining cell populations). Empirically, we determined to gate "high fluorescing" variants at >1E4 and "low fluorescing" variants at <1E4. See Methods for details.

- [x] Tick this box to confirm that a figure exemplifying the gating strategy is provided in the Supplementary Information.
